# Economic impacts of tipping points in the climate system

**DOI:** 10.1073/pnas.2103081118

**Published:** 2021-08-16

**Authors:** Simon Dietz, James Rising, Thomas Stoerk, Gernot Wagner

**Affiliations:** ^a^Department of Geography and Environment, London School of Economics and Political Science, London WC2A 2AE, United Kingdom;; ^b^Grantham Research Institute on Climate Change and the Environment, London School of Economics and Political Science, London WC2A 2AE, United Kingdom;; ^c^College of Earth, Ocean and Environment, University of Delaware, Newark, DE 19716;; ^d^Department of Environmental Studies and Robert F. Wagner Graduate School of Public Service, New York University, New York, NY 10003

**Keywords:** climate tipping points, social cost of carbon, integrated assessment model, climate risk

## Abstract

Climate scientists have long emphasized the importance of climate tipping points like thawing permafrost, ice sheet disintegration, and changes in atmospheric circulation. Yet, save for a few fragmented studies, climate economics has either ignored them or represented them in highly stylized ways. We provide unified estimates of the economic impacts of all eight climate tipping points covered in the economic literature so far using a meta-analytic integrated assessment model (IAM) with a modular structure. The model includes national-level climate damages from rising temperatures and sea levels for 180 countries, calibrated on detailed econometric evidence and simulation modeling. Collectively, climate tipping points increase the social cost of carbon (SCC) by ∼25% in our main specification. The distribution is positively skewed, however. We estimate an ∼10% chance of climate tipping points more than doubling the SCC. Accordingly, climate tipping points increase global economic risk. A spatial analysis shows that they increase economic losses almost everywhere. The tipping points with the largest effects are dissociation of ocean methane hydrates and thawing permafrost. Most of our numbers are probable underestimates, given that some tipping points, tipping point interactions, and impact channels have not been covered in the literature so far; however, our method of structural meta-analysis means that future modeling of climate tipping points can be integrated with relative ease, and we present a reduced-form tipping points damage function that could be incorporated in other IAMs.

## Significance

Tipping points in the climate system are one of the principal reasons for concern about climate change. Climate economists have only recently begun incorporating them in economic models. We synthesize this emerging literature and provide unified, geophysically realistic estimates of the economic impacts of eight climate tipping points with an emphasis on the social cost of carbon, a key policy input.

Climate tipping points are subject to considerable scientific uncertainty in relation to their size, probability, and how they interact with each other (1–4). Their economic impacts are even more uncertain, and consequently, these are often ignored (5, 6) or given a highly stylized treatment that fails to accurately represent geophysical dynamics and is nearly impossible to calibrate (7–9). As a result, tipping points are only weakly reflected in the policy advice economists give on climate change, typically by way of caveats and contextualization, rather than an integral part of the modeling that gives rise to estimates of the social cost of carbon (SCC) and other economic metrics of interest.

The very definition of climate tipping points has attracted significant scholarship (2, 9, 10). We associate them with perhaps the best-known definition of “tipping elements”: “subsystems of the Earth system that are at least subcontinental in scale and can be switched—under certain circumstances—into a qualitatively different state by small perturbations” (2). This is an intentionally broad and flexible definition that admits a variety of geophysical responses, including nonlinear feedbacks and both reversible and irreversible phase changes (9). This flexibility is important for our purposes because economic studies omit or inadequately capture geophysical processes of all these sorts. Adopting a narrower definition (for example, limited to abrupt, discontinuous changes) would lead us to exclude geophysical processes with large economic costs.

## Economic Analysis of Climate Tipping Points

A growing body of research has explored climate tipping points using economic models. We reviewed this literature and identified 52 papers that model the economic consequences of at least one climate tipping point (*SI Appendix*, Table S1). Many of these studies, however, represent climate tipping points in a highly stylized way. Examples include an instantaneous jump in the model’s equilibrium climate sensitivity (11), an arbitrary reduction in global gross domestic product (GDP) (12), and a one-off permanent reduction in global utility (13). While such studies have helped put climate tipping points on the economic research agenda and contributed to understanding qualitative aspects of climate policy in the face of tipping points, such stylized representations are unrealistic from a geophysical point of view and difficult to calibrate quantitatively. Therefore, we also identified those studies that are based on geophysical foundations (i.e., with at least a reduced-form representation of the key underlying geophysical relationship[s] that govern the tipping point). This yielded 21 articles, highlighted in *SI Appendix*, Figs. S1–S3 and Table S1.

The literature presents several challenges to developing a comprehensive synthesis. Each study takes an individual tipping point or a few tipping points and employs a particular integrated assessment model (IAM) with its idiosyncratic structure. In doing so, different studies have imposed different boundary conditions (e.g., greenhouse gas or GHG emissions scenarios), made different choices on common parameters (e.g., those governing the discount rate), and even used different welfare metrics to report their results (e.g., marginal vs. total costs). Furthermore, there are many interactions between tipping points (14), and there is no simple way to capture those interactions using basic methods of literature synthesis.

## A “Meta-Analytic” IAM

Therefore, we developed a method to synthesize this literature: a meta-analytic IAM that includes replicas of each tipping point module in the literature and integrates them into one consistent framework. A standard meta-analysis attempts to combine multiple estimates of the same treatment effect (e.g., multiple trials of the same drug). Here, we study several mutually exclusive “treatment effects” (i.e., tipping points), necessitating a nested, structural model. One could therefore call the approach “structural meta-analysis.” Table 1 lists the tipping point modules replicated in this study, spanning eight tipping points, which are broadly divided into 1) positive carbon-cycle and temperature feedbacks, 2) ice sheet disintegration, and 3) changes in large-scale circulation. Some models of prior studies are “process based,” with each equation corresponding to a geophysical process, at least in reduced form, that can be calibrated on the underlying scientific literature. The tipping processes in these models tend not to be abrupt (e.g., gradual thawing of permafrost). Other models use survival analysis, whereby a tipping event can occur in each period with a probability that increases with temperature. The tipping process in this class of models is abrupt, but the impacts need not be (e.g., the slow rise of sea levels upon triggering disintegration of the West Antarctic ice sheet [WAIS]). A key principle applied here is to exactly replicate the relevant elements of these economic studies. That is, we do not override the modelers’ original choices on structure and parameters. We only augment them using the underlying scientific literature if warranted (e.g., converting a “what if” scenario into a probabilistic event).

**Table 1.** Models synthesized in this study

Tipping point	Papers	IAM	Model of TP	Uncertainty
Permafrost carbon feedback (PCF)	Hope and Schaefer (24)	PAGE09	Process based	MC
	Kessler (25)	DICE	Process based	Deterministic and MC
	Yumashev et al. (23)	PAGE-ICE	Process based	MC
Ocean methane hydrates (OMH)	Ceronsky et al. (50)	FUND	Tipping event	Deterministic and MC
	Whiteman et al. (51)	PAGE09	Tipping event	MC
Arctic sea ice/Surface Albedo Feedback (SAF)	Yumashev et al. (23)	PAGE-ICE	Process based	MC
Amazon dieback (AMAZ)	Cai et al. (14)	DSICE	Tipping event	Survival analysis
GIS disintegration	Nordhaus (19)	DICE	Process based	Deterministic
WAIS disintegration	Diaz and Keller (47)	DICE	Tipping event	Survival analysis
Atlantic Meridional Overturning				
Circulation (AMOC) slowdown	Anthoff et al. (22)	FUND	Tipping event	Deterministic
Indian summer monsoon	Belaia (48) using Schewe			
(ISM) variability	and Levermann (52)	RICE	Process based	Stochastic

MC, Monte Carlo simulation.

The nature of some tipping points places requirements on the specification of our meta-analytic IAM. First, inclusion of thawing permafrost and possible dissociation of ocean methane hydrates makes it important to explicitly model radiative forcing from methane (${CH}_{4}$) emissions. Second, inclusion of disintegration of the Greenland ice sheet (GIS) and the WAIS makes it important to explicitly model sea-level rise (SLR) and corresponding damage. Third, inclusion of tipping points related to changes in the atmospheric circulation, which have heterogeneous effects worldwide, makes it important to disaggregate damages to the national level. To do so, we utilize recent empirical and simulation results on the impacts of temperature and SLR, which arguably constitute the best available evidence at present (15, 16). *Methods* and *SI Appendix*, section 2 have more details on the meta-analytic IAM.

## Results

**Fig. 1.**
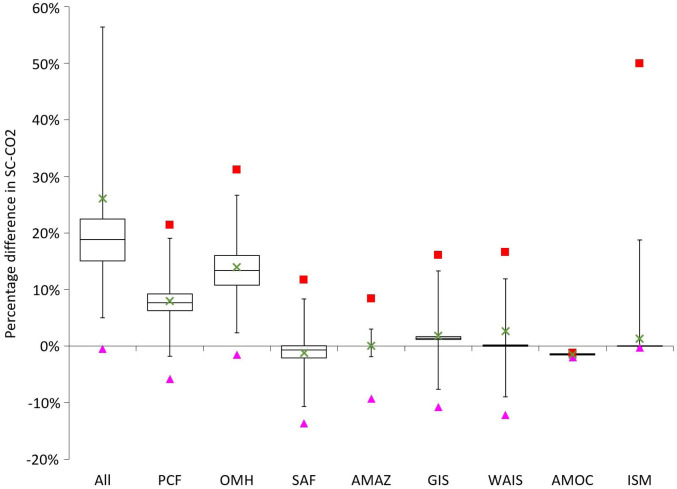
The percentage change in the SCC due to tipping points collectively and individually. Boxes show medians and interquartile ranges, whiskers show 95% CIs, crosses mark the average changes (0.1% trimmed), triangles mark the 0.5 percentiles, and squares mark the 99.5 percentiles. The *y* axis is truncated. Specification comprises RCP4.5-SSP2 emissions and GDP/population growth, Hope and Schaefer PCF, Whiteman et al. beta OMH, and IPSL AMOC hosing. Monte Carlo sample size is 10,000.

Our main economic impact metric is the SCC, the economic cost of emitting one additional ton of ${CO}_{2}$ (i.e., the marginal damage cost). The SCC is perhaps the key welfare measure of climate change in policy discussions, as it can be used to set carbon prices and inform mitigation efforts (17, 18). Table 2 reports the change in the expected SCC due to tipping points in our main specification. These results derive from a Monte Carlo simulation with a sample size of 10,000. Variation comes from many probabilistic parameters, including probabilistic tipping events. Our main specification omits “nonmarket” impacts of climate change, such as those on ecosystems and human health. We include an estimate of these in our sensitivity analysis instead (see below). Combining all eight tipping points increases the expected SCC by 24.5%. As discussed below, this should be seen as a probable underestimate, given the literature we synthesize has yet to cover some tipping points, and misses possible impact channels and interactions even for those it does cover. Fig. 1 shows that the distribution of expected increases in the SCC is positively skewed. The median percentage increase in the SCC from all tipping points combined is 18.8%; the 75th percentile is 22.5%, and the 99.5th percentile is 132.2%.

**Table 2.** The SCC (2020 US dollars) and the percentage change in the SCC due to tipping points collectively and individually

	Expected SCC,	Increase due
TP	US$/${tCO}_{2}$	to TP, %
None	52.03	—
Permafrost carbon	56.41	8.4
Ocean methane hydrates	58.85	13.1
SAF	51.14	−1.7
Amazon	52.07	0.1
GIS	52.97	1.8
WAIS	53.57	2.9
AMOC	51.28	−1.4
Indian summer monsoon	52.70	1.3
All TPs	64.80	24.5
∑ main effects, all TPs	—	24.5
All costly TPs	67.05	28.9
∑ main effects, costly TPs only	—	27.6

The expected SCC is computed over 10,000 Monte Carlo draws with 0.1% trimmed. Specification comprises RCP4.5-SSP2 emissions and GDP/population growth, Hope and Schaefer PCF, Whiteman et al. beta OMH, and IPSL AMOC hosing. TP, tipping point.

The individual tipping points contributing most to the increase in the SCC are dissociation of ocean methane hydrates, which in itself increases the expected SCC by 13.1%, and the permafrost carbon feedback (+8.4%). Disintegration of the WAIS increases the expected SCC by 2.9%. Disintegration of the GIS increases the expected SCC by 1.8%, similar to Nordhaus’ (19) recent estimate on which the GIS module is based. *SI Appendix*, Fig. S12 shows, however, that the WAIS and GIS modules predict lower contributions to SLR from melting of the respective ice sheets than the process-based models synthesized in the Intergovernmental Panel on Climate Change’s Fifth Assessment Report (IPCC AR5) (20). Therefore, the increase in the SCC due to ice sheet disintegration may be underestimated. Variability of the Indian summer monsoon and associated floods and droughts in India is significant enough to register at the global level, increasing the expected SCC by 1.3%. Dieback of the Amazon rainforest leads to a modest 0.1% increase in the expected SCC. This is based on the assumption in the model we replicate that, upon crossing the tipping threshold, dieback releases 50 GtC over 50 y (14), which equates to only about 5 y of ${CO}_{2}$ emissions from fossil fuel and industry at current rates (21). No other costs of Amazon rainforest dieback have yet been included in the literature, even though they could be considerable. Two tipping points reduce the expected SCC. Slowdown of the AMOC reduces the expected SCC by 1.4% by reducing damaging warming in some countries. The sign of the effect we find is consistent with the underlying study we replicate (22), even though damages are modeled differently. The Surface Albedo Feedback (SAF) reduces the expected SCC by 1.7%. Unlike other tipping points, a constant level of SAF is included in standard equilibrium climate sensitivity values. The SAF model we include, introduced by ref. 23, describes the changing capacity for sea ice and land snow to respond to warming. As the area of ice and snow decreases, which increases albedo forcing, further warming produces smaller albedo changes, which reduce the effective equilibrium climate sensitivity. These changes increase temperatures in the short term, but they reduce temperatures over the long term and decrease the SCC, consistent with the underlying study we replicate (*SI Appendix*).

When modeled separately and then summed together, the individual tipping points also increase the expected SCC by 24.5%. Therefore, interactions between tipping points that are embodied in the meta-analytic IAM (*SI Appendix*, section 2.1.9) make no difference to the overall effect. However, this does not mean interactions between tipping points are entirely unimportant. Rather, it is the result of positive interactions being offset by negative interactions. To substantiate this point, Table 2 also reports the increase in the expected SCC due to the six tipping points that cause net economic costs (i.e., minus AMOC slowdown and SAF weakening). This is 28.9% compared with 27.6% when summing the six tipping points together. In this case, positive interactions increase the expected SCC by a further 1.3 percentage points. When AMOC slowdown and SAF weakening are reintroduced, their overall effect in interaction with each other and with the other tipping points is larger than their individual effects.

**Fig. 2.**
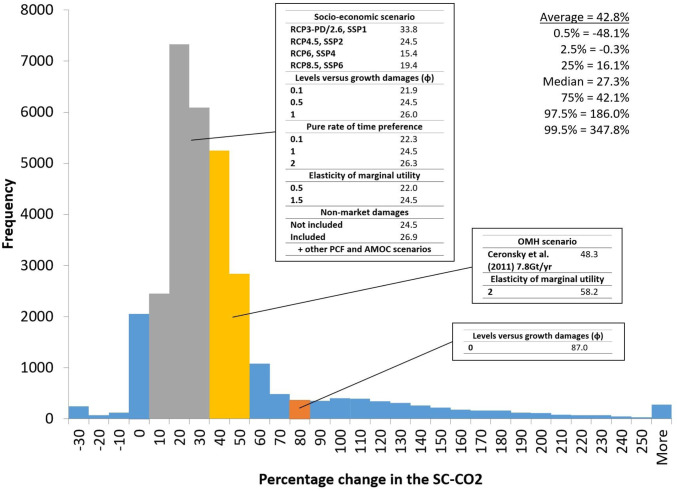
Infographic summarizing uncertainty about the percentage change in the SCC due to tipping points and the sources of that uncertainty. Histogram shows the full distribution of percentage changes in the SCC from a pooled Monte Carlo sample of size 32,000 (*SI Appendix* has further details). Percentage changes reported in the boxes are expected values for one factor at a time variations on the following specification: RCP4.5-SSP2 emissions and GDP/population growth, Hope and Schaefer PCF, Whiteman et al. beta OMH, and IPSL AMOC hosing. Note that the result for the OMH scenario includes all eight tipping points on.

We augment the main specification of the model with extensive uncertainty analysis to explore robustness as well as tail risks. *SI Appendix*, section 3.2 reports a wide range of sensitivity analyses. The results of these are summarized in Fig. 2. The effect of the permafrost carbon feedback is similar across the three available published studies (23–25). The effect of dissociation of ocean methane hydrates in our main scenario is robust to different calibrations of the hazard rate and different durations of the emissions impulse, but it is not robust to different emissions impulse scenarios. Rather, the increase in the expected SCC ranges from 4.1 to 49.2% across scenarios reported in the two available studies, commensurate with the widely varying amounts of ${CH}_{4}$ released in these scenarios, the spread of which reflects uncertainty in the underlying science. All AMOC slowdown scenarios result in a decrease in the expected SCC ranging from −0.7 to −5.7%, the latter in a scenario with a notably large two-thirds slowdown in the circulation. The percentage increase in the expected SCC due to all eight tipping points combined is relatively consistent across different emissions/socioeconomic scenario pairs and across variations in two key economic parameters governing the welfare value of climate damages, namely the pure rate of time preference and the elasticity of marginal utility of consumption. The exception to this is when the elasticity of marginal utility of consumption is set to a relatively high value of two. This implies inter alia relatively high risk aversion. In this case, the increase in the expected SCC is 58.2%, although the median percentage increase is only 22.0% and the 75th percentile increase is only 30.3%. Hence, this result is driven by a small number of runs in the right tail of the distribution and the disproportionate effect they have on the expected SCC under high risk aversion. *SI Appendix*, Fig. S20 and Table S13 report the effect of including a leading estimate of global nonmarket damages from climate change using the nonmarket damage module from the MERGE (Model for Evaluating Regional and Global Effects of GHG reductions policies) IAM (26). The resulting estimates of the SCC are more comprehensive but arguably more uncertain. The effect of all tipping points combined on the expected SCC increases marginally, to 26.9%.

As well as high risk aversion, parametric uncertainty relating to the structure of climate damages can also strongly affect how tipping points increase the SCC. We adopt a flexible specification of climate damages that is able to capture the range of assumptions in the literature about whether climate damages impact the level of economic activity or its growth rate. This is an area of active research in climate economics. In our model, the level of income per capita in the previous year, on which damages in the current year work, is given by

$$\bar{y}\left(i,t-1\right)=\varphi y_{EX}\left(i,t-1\right)+\left(1-\varphi \right)y\left(i,t-1\right),$$ [1]

where $y_{EX}\left(i,t-1\right)$ is counterfactual income per capita in country i in year t−1 taken from an exogenous socioeconomic scenario, y(i,t−1) is actual postdamage income per capita experienced in the previous year, and φ∈[0,1] parameterizes the weight given to each. This specification enables us to explore two different extreme interpretations of the empirical evidence on damages (mainly in relation to temperature), as well as combinations of them. The first interpretation (φ=1) is that damages solely impact the level of income in each year, in effect driving a wedge between what output is feasible given implicit factors of production and productivity and what output is actually achieved. This has been the traditional approach in climate economics (27). The second interpretation (φ=0) is that temperatures entirely impact the growth rate of income by directly impacting the accumulation of factors of production and/or by impacting productivity growth (15, 28–30). Our main specification is an intermediate value of φ=0.5. *SI Appendix*, Fig. S17 and Table S10 show that the expected increase in the SCC due to tipping points is relatively robust to variations in φ across most of its range. However, when φ=0—pure growth damages—both the SCC and the effect of tipping points on the SCC increase dramatically. The SCC is now thousands of dollars, consistent with a previous study that also simulated pure growth damages at the national level (30). Tipping points increase the expected SCC by 87.0%, as the initial effect propagates over time. Large increases are observed across the distribution, as *SI Appendix*, Fig. S17 makes clear.

**Fig. 3.**
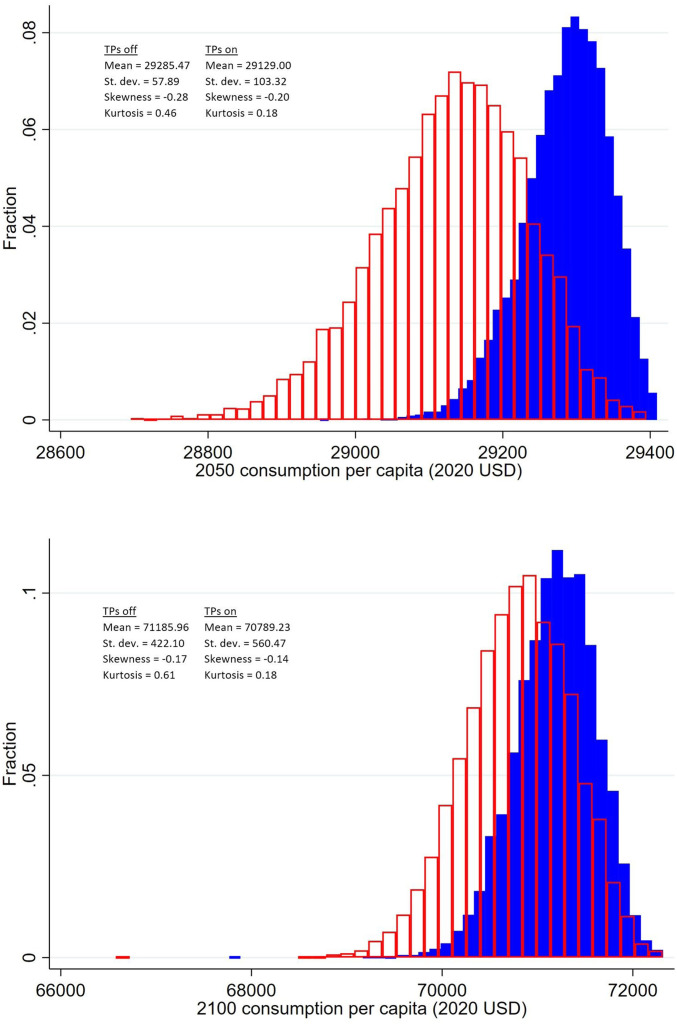
Histograms showing the distribution of world mean consumption per capita in 2050 (*Upper*) and 2100 (*Lower*) both without tipping points (blue) and with tipping points (red). Main specification comprises RCP4.5-SSP2 emissions and GDP/population growth, Hope and Schaefer PCF, Whiteman et al. beta OMH, and IPSL AMOC hosing. Monte Carlo sample size is 10,000. Values reported are in 2020 US dollars. TP, tipping point; USD, US dollars.

Across all parametric and scenario uncertainties, we estimate an expected increase in the SCC of 42.8% due to climate tipping points (*SI Appendix*, section 3.2.9 has details). We estimate a 95% CI of −0.3 to +186.0%. The distribution is positively skewed, and the SCC is at least doubled in roughly 10% of sample runs. This suggests that tipping points have consequences for the overall level of risk borne by the world economy in the future, which has implications for financial markets: for instance, via the equity risk premium (31). This is explored more directly in Fig. 3, which compares the distribution of global mean consumption per capita in 2050 and 2100 with and without tipping points. The eight tipping points not only reduce global mean consumption per capita, but also, they significantly increase the dispersion. Therefore, tipping points increase future consumption risk. The extra dispersion is greater in 2050 than in 2100; tipping points increase the coefficient of variation by 79% in 2050 and 34% in 2100.

**Fig. 4.**
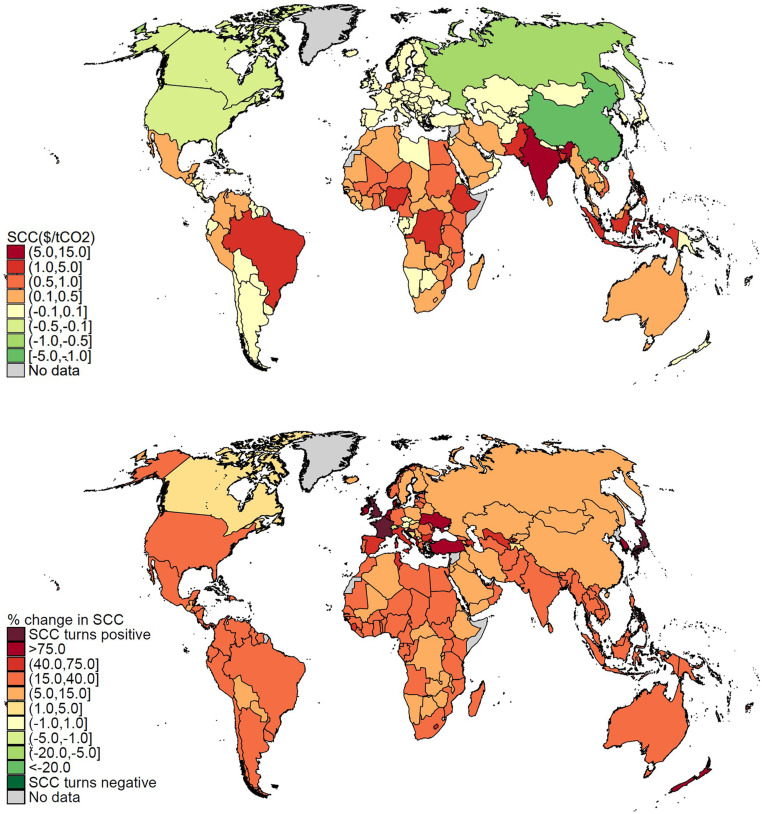
Country-level expected SCC estimates (2020 US dollars) without tipping points (*Upper*) and the percentage change in the expected country-level SCC due to all tipping points (*Lower*). Welfare changes are normalized to global mean consumption per capita. Specification comprises RCP4.5-SSP2 emissions and GDP/population growth, Hope and Schaefer PCF, Whiteman et al. beta OMH, and IPSL AMOC hosing. Monte Carlo sample size is 10,000 with 0.1% trimmed.

In addition to aggregate economic risk, tipping points might affect the distribution of climate impacts. We exploit the country-level resolution of our meta-analytic IAM to analyze this, estimating national SCCs. Fig. 4, *Upper* shows that the SCC is unequally distributed before tipping points are factored in. It tends to be higher in hotter, poorer regions, such as South and Southeast Asia and sub-Saharan Africa. Some colder, high-latitude countries in the Northern Hemisphere see a negative SCC (i.e., net benefits from climate change). This is consistent with econometric evidence on temperature (15, 32); however, note that our results also include damages from SLR (16), increasing relative costs in some countries. Fig. 4, *Lower* shows how the inclusion of tipping points affects each country’s SCC. Almost all countries (98%) see their SCC increase. While the size of the increase varies from country to country, tipping points do not materially alter how climate change affects income inequality. One way to measure this is by computing the Gini coefficient of national SCCs (30). We calculate this to be 0.64 when tipping points are included, compared with 0.66 without tipping points. *SI Appendix*, section 3.5 visualizes this using Lorenz curves. Another way to measure this is the correlation (population weighted) between national GDP per capita (2020; purchasing power parity) and national SCCs. This correlation is −0.326 in the absence of tipping points; more developed countries experience a lower SCC, and the impacts of climate change are thus regressive. Tipping points increase this negative correlation only very slightly to −0.335. *SI Appendix*, section 3.3 plots the effect on national SCCs of each individual tipping point. Disintegration of the GIS and WAIS primarily affects countries with low-lying coastal populations. The permafrost carbon feedback, dissociation of ocean methane hydrates, and the SAF primarily affect temperature. For these tipping points, country-level impacts depend on whether a country is below or above its optimum temperature, and for the permafrost carbon feedback and SAF, there is a clear association with latitude. AMOC slowdown benefits Europe, while parts of central Asia see increased climate damages.

**Fig. 5.**
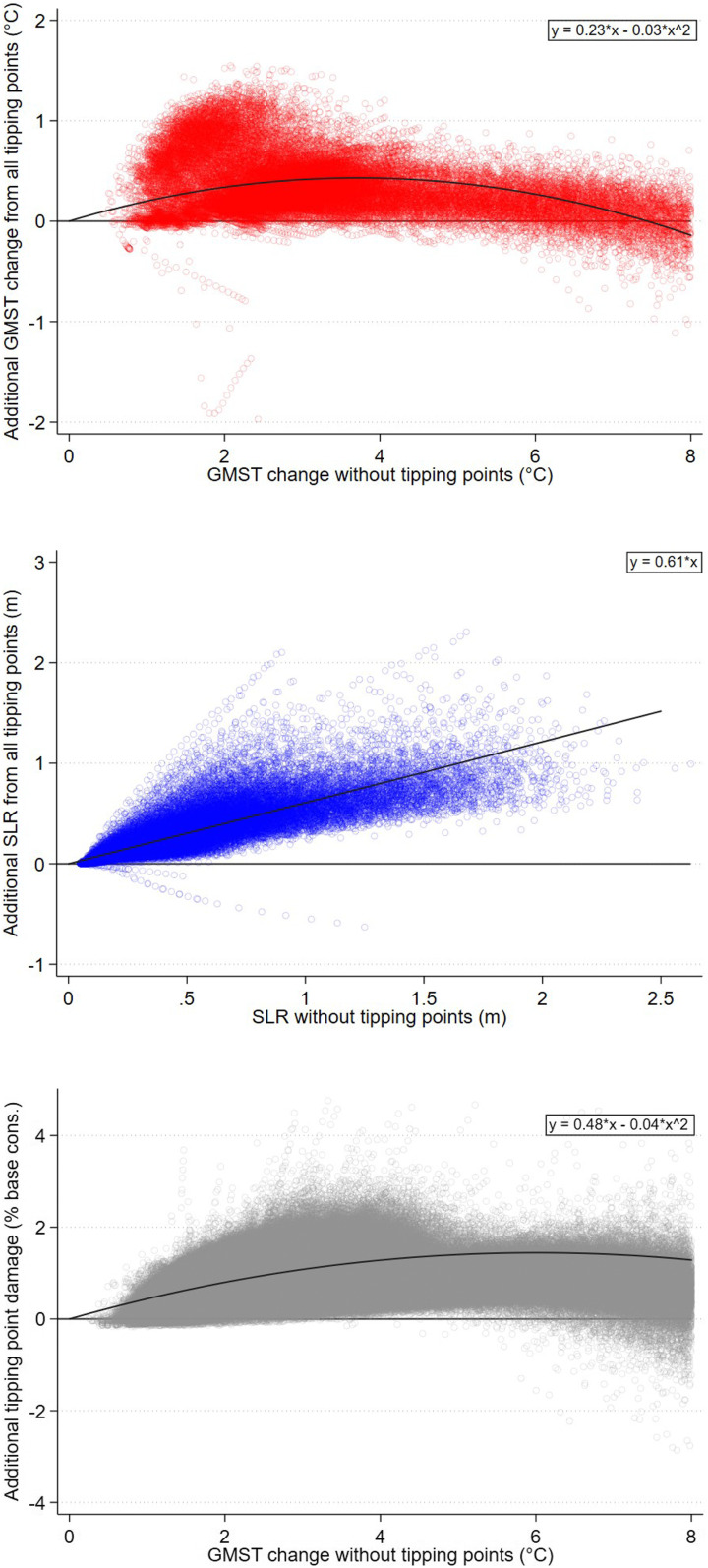
Scatterplots of additional warming (*Top*), SLR (*Middle*), and damages (*Bottom*) from all tipping points combined. Data are sampled on a decadal interval (2020, 2030,...2200) using 1,000 Monte Carlo simulations under each of the RCP4.5 and RCP8.5 emissions scenarios. A nonlinear fit is used for temperature and damages; a linear fit is used for SLR. Incremental damage from tipping points is expressed as the change in world consumption per capita due to tipping points, relative to world consumption per capita without climate damages. Specification comprises Hope and Schaefer PCF, Whiteman et al. beta OMH, and IPSL AMOC hosing. GMST, global mean surface temperature.

Fig. 5 shows the combined effects of the eight tipping points on warming and SLR. These scatterplots are generated by pooling decadal temperature and SLR data in the RCP4.5 and RCP8.5 emissions scenarios, giving full coverage of the range of physical changes possible over the next two centuries. Tipping points increase the temperature response to GHG emissions over most of the range of temperatures attained (Fig. 5, *Top*). Using a second-order polynomial to fit the data, 2°C warming in the absence of tipping points corresponds to 2.3°C warming in the presence of tipping points, for instance. In some model runs, tipping points add as much as 1.5°C additional warming. Beyond c. 7°C warming in the absence of tipping points, the combined effect of tipping points is to reduce the temperature response to GHG emissions. *SI Appendix*, section 3.4 disaggregates the temperature response by tipping point. It shows that the initially greater temperature response is primarily due to dissociation of ocean methane hydrates and to a lesser extent, the permafrost carbon feedback, while the eventually lower temperature response is due entirely to the weakening SAF. Tipping points always increase SLR (Fig. 5, *Middle*). A linear fit of the data implies 61% more SLR at any given level. *SI Appendix*, section 3.4 shows that melting of the GIS and WAIS each contributes roughly half of the total.

In climate economics, a central relationship is the damage function (i.e., the reduced-form relationship mapping global mean temperature increases into contemporaneous welfare losses). Fig. 5, *Bottom* plots the additional consumption losses from the eight tipping points as a function of temperature. This reduced-form climate tipping points damage function could be integrated in other IAMs, which work at this higher level of aggregation. Tipping points reduce global consumption per capita by around 1% upon 3°C warming and by around 1.4% upon 6°C warming, based on a second-order polynomial fit of the data. In some runs, damages exceed 4%. These patterns strongly reflect the underlying physical changes in Fig. 5, *Top* and *Middle*. Dissociation of ocean methane hydrates and the permafrost carbon feedback elevate the temperature response to given greenhouse gas emissions, resulting in higher damages. Although the weakening SAF provides a countervailing effect, additional SLR damages from disintegration of the GIS and WAIS mean that the point at which incremental damages from tipping points turn negative is not reached until c. 10°C. The SCC is calculated by converting these consumption losses into utility losses and then taking the discounted sum from 2020 until the end of the modeling horizon.

## Conclusion

In this paper, we have synthesized an emerging but fragmented literature modeling the economic impacts of climate tipping points. Our aim has been to develop a more quantitative, structured understanding of the whole issue, so that climate tipping points are better reflected in the policy advice economists give on climate change (33). The eight tipping points that have been modeled in climate economics to date affect temperatures or sea levels in diverse ways. Most increase the SCC, especially the carbon-cycle feedbacks associated with the release of GHGs trapped in permafrost and ocean sediments. Two tipping points reduce the SCC; AMOC slowdown puts a brake on damaging warming in some countries, especially large economies in northwestern Europe, while the SAF weakens over time, contributing less radiative forcing than the standard assumption of constant climate sensitivity. As well as increasing climate damages overall, our second key finding is that climate tipping points increase the overall level of risk in the global economy. This increases the expected SCC because risk has a social cost when society is risk averse. As we have seen, under high risk aversion the premium on the expected SCC is large. It also has implications for financial markets, where higher risks typically require higher returns to investors as compensation. Third, we find that climate tipping points increase economic costs almost everywhere, and these additional costs are spread relatively evenly, so that tipping points do not have a significant effect on inequality. Lastly, we provide a straightforward way of augmenting the damage function in IAMs that works with a simple, reduced-form relationship between temperature and economic losses.

## Discussion

Our research is subject to a number of limitations, which help to identify future research needs. First, although we have been able to combine eight different climate tipping points, other climate tipping points have been identified, which have yet to be included in climate–economic IAMs (2). Examples include Boreal forest dieback, variability of the West African monsoon, and the El Niño Southern Oscillation, the last of which we only cover as it affects the Indian summer monsoon. Our modular approach facilitates the inclusion of additional tipping points in future (in principle, all that is required is that they are driven by, and affect, existing variables within the model). Second, our coverage of interactions between tipping points is incomplete. *SI Appendix*, Table S4 summarizes the interactions we do include. Some are hardwired in the structure of our meta-analytic IAM. For example, the permafrost carbon feedback affects all seven other tipping points via global mean temperature. Other interactions not related to global mean temperature are incorporated using estimates from an expert elicitation study (34). This leaves 12 (of 56) interactions that are not modeled. Third, there could be missing climate impacts, even of tipping points that we do include. Perhaps the easiest to envisage are some of the impacts of Amazon rainforest dieback, such as lost biodiversity and ecosystem service flows. Another example is AMOC slowdown, which is likely to lead to impacts that go beyond temperature. These include ocean acidification and a decrease in marine productivity, as well as changed wind and precipitation patterns (35). We have included a nonmarket damage function in our sensitivity analysis, but this responds to changes in global mean temperature and does not reflect forest dieback specifically. Fourth, the tipping point modules we replicate in this study are subject to uncertainties, no more so perhaps than dissociation of ocean methane hydrates. Fifth, our meta-analytic IAM is affected by some well-known controversies and uncertainties, including those in climate science (e.g., equilibrium climate sensitivity) and in economics (e.g., the discount rate). Fortuitously, most of these uncertainties appear not to matter greatly when estimating the effect of tipping points on the SCC. One notable exception, however, is the extent to which climate damages affect the level or growth rate of output and how this is related to countries’ development level. Our economic model includes a standard treatment of utility and welfare, but many recent extensions have been proposed in climate economics, and these often increase the SCC (e.g., refs. 36–39).

## Methods

The meta-analytic IAM is described in complete detail in *SI Appendix*. Its central features can be summarized as follows.

### Anthropogenic Greenhouse Gas Emissions, Growth, and Population Projections.

Greenhouse gas emissions and corresponding baseline projections of GDP and population growth are exogenous and taken from the Representative Concentration Pathway (RCP)/Shared Socio-Economic Pathway (SSP) database (40, 41). We match RCP3-PD/2.6 with SSP1, RCP4.5 with SSP2 and SSP5, RCP6 with SSP4, and RCP8.5 with SSP5. Since we estimate the SCC, it is important that our emissions scenarios extend beyond 2100. Therefore, we use the Extended Concentration Pathways database for emissions (42) and develop a method of extending the corresponding SSPs beyond 2100 (*SI Appendix*). ${CO}_{2}$ and ${CH}_{4}$ emissions are modeled explicitly. Other GHGs and forcing agents are combined into an exogenous vector of residual radiative forcing.

### Atmospheric Chemistry and Warming.

The Finite Amplitude Impulse Response (FAIR) model is used to represent the carbon cycle (43). FAIR extends a model with four boxes (i.e., impulse response functions of different timescales) that was used to emulate the behavior of carbon-cycle models of different complexity, which fed into IPCC AR5 (44). FAIR adds a positive feedback from cumulative ${CO}_{2}$ uptake and temperature to the rate of ${CO}_{2}$ uptake. This chiefly captures saturation of the ocean carbon sink. Radiative forcing from ${CO}_{2}$ at time t is a log function of the ratio of the atmospheric ${CO}_{2}$ concentration at time t and the preindustrial concentration. Radiative forcing from ${CH}_{4}$ is modeled explicitly. After being emitted to the atmosphere, ${CH}_{4}$ decays exponentially with an atmospheric lifetime of 12.4 y (45). Radiative forcing is modeled according to IPCC AR5 (45). Radiative forcing is a square root function of the atmospheric concentration of ${CH}_{4}$ in excess of preindustrial, with codependence on atmospheric $N_{2}$O in the initial model year (2010). Warming is simulated using a two-box model of heat transfer between the atmosphere and upper oceans and the deep oceans, which is calibrated on the WCRP Coupled Model Intercomparison Project Phase 5 (CMIP5) ensemble (46). The inputs are radiative forcing from ${CO}_{2}$, ${CH}_{4}$, and the vector of other GHGs and forcing agents. *SI Appendix*, Fig. S11 compares the temperature projections of our climate module with the corresponding projections of the CMIP5 ensemble and shows that they are in close agreement.

### Country-Level Temperature Damages.

Changes in global mean surface temperature are disaggregated to the national level using nonlinear statistical downscaling. Changes in national mean surface temperature are then fed into nonlinear, country-specific damage functions calibrated on recent empirical evidence (15).

### Country-Level Damages from SLR.

Changes in global mean surface temperature drive global mean SLR via thermal expansion and melting of small ice caps and glaciers (plus additional SLR from the GIS and WAIS tipping modules) (19, 47). *SI Appendix*, Fig. S12 compares our SLR projections with the projections of process-based models synthesized in IPCC AR5 (20). The projections of total SLR are similar, comprising a larger contribution from thermal expansion, small ice caps, and glaciers in our model offset by a smaller contribution from GIS and WAIS disintegration in our model, dictated by the tipping point modules we replicate. Global mean SLR is mapped on damages at the national level using recent high-resolution modeling results (16).

### Flood and Drought Due to the Indian Summer Monsoon.

In India, GDP is additionally affected by variability of the summer monsoon, which determines the occurrence of drought or flood according to the ISM tipping module (48).

### Levels vs. Growth Damages.

We adopt a flexible specification allowing damages from temperature and SLR (and in India, from the summer monsoon) to affect either the short-term level of GDP or long-term growth prospects. In our main specification, we assign weights of 1/2 to both damage channels (φ=0.5) based on the principle of insufficient reason, which accounts for the fact that the empirical evidence on damage channels is only tentative. In our uncertainty quantification, we specify a uniform distribution with end points corresponding to full weight on the pure levels (φ=1) and growth (φ=0) specifications, respectively (49).

### Consumption and Welfare.

National GDP per capita is converted into national consumption per capita using country-specific exogenous savings rates, estimated using World Bank data on savings over the period 2005 to 2015. We specify an isoelastic utility function with an elasticity of marginal utility of consumption of 1.5 in our main specification and a utilitarian social welfare functional with a constant pure rate of time preference of 1% in our main specification. In our uncertainty quantification, the elasticity of marginal utility of consumption is triangular distributed with a minimum of 0.5, mode of 1.5, and maximum of 2, while the pure rate of time preference is triangular distributed with a minimum of 0.1%, mode of 1%, and maximum of 2%.

### Tipping Point Modules.

There are eight tipping modules, corresponding to the tipping points listed in Table 1. Each module replicates the underlying studies listed in column 2 of Table 1. Their roles in the model are as follows.

• The permafrost carbon feedback results in additional ${CO}_{2}$ and ${CH}_{4}$ emissions, which flow back into the ${CO}_{2}$ and ${CH}_{4}$ cycles.
• Dissociation of ocean methane hydrates results in additional ${CH}_{4}$ emissions, which flow back into the ${CH}_{4}$ cycle.
• Arctic sea ice loss (also known as the SAF) results in changes in radiative forcing, which directly affects warming.
• Dieback of the Amazon rainforest releases ${CO}_{2}$, which flows back into the ${CO}_{2}$ cycle.
• Disintegration of the GIS and WAIS increases SLR.
• Slowdown of the AMOC modulates the relationship between global mean surface temperature and national mean surface temperature.
• Variability of the Indian summer monsoon directly affects GDP per capita in India.

### SCC.

To estimate the SCC, we run the model twice with consistent assumptions, the second time with an additional pulse of emissions in the year 2020. The SCC is the scaled difference in welfare between the two runs per ton of ${CO}_{2}$ emissions. Each run typically involves a Monte Carlo simulation with a sample size of 10,000.

## Supplementary Material

Supplementary File

## Data Availability

Simulation model data have been deposited in GitHub (https://github.com/openmodels/META-2021).

## Change History

September 1, 2021: The classifications have been updated.
