# Supplementary material for: Economic impacts of tipping points in the climate system
Source: Proc Natl Acad Sci U S A. 2021 Aug 16;118(34):e2103081118. doi: 10.1073/pnas.2103081118 (PMC8403967; doi:10.1073/pnas.2103081118)
Supplement: Supplementary File [file pnas.2103081118.sapp.pdf]

Economic impacts of tipping points in the climate system: supplementary information

Simon Dietz[1,2*], James Rising[3], Thomas Stoerk[2] & Gernot Wagner[4]

July 13, 2021

1. Department of Geography and Environment, London School of Economics and Political Science, Houghton St., London WC2A 2AE, UK

2. Grantham Research Institute on Climate Change and the Environment, London School of Economics and Political Science, Houghton St., London WC2A 2AE, UK

3. College of Earth, Ocean and Environment, University of Delaware, Newark, DE 19716, USA

4. Department of Environmental Studies, New York University and NYU Wagner, 285 Mercer St., New York, NY 10003, USA

*Corresponding author; e-mail s.dietz@lse.ac.uk

Contents

1 Literature review **4**

2 Model description **7**

2.1 Tipping point modules 10

2.1.1 Permafrost carbon feedback 10

2.1.2 Ocean methane hydrates 14

2.1.3 Amazon rainforest dieback 17

2.1.4 Greenland Ice Sheet 18

2.1.5 West Antarctic Ice Sheet 20

2.1.6 Arctic sea-ice loss/surface albedo feedback 21

2.1.7 Slowdown of the Atlantic Meridional Overturning Circulation 25

2.1.8 Weakening of the Indian Summer Monsoon 27

2.1.9 Tipping point interactions 30

2.2 Climate module 33

2.2.1 Emissions 33

2.2.2 CO_2 and CH_4 cycles 34

2.2.3 Radiative forcing and temperature 36

2.3 Damages/economic module 37

2.3.1 Sea level rise 37

2.3.2 National temperature 38

2.3.3 Damages and national income per capita 39

2.3.4 Utility and welfare 41

2.3.5 Computing the social cost of carbon 42

2.3.6 Non-market damages 44

2.4 Supporting analysis 46

2.4.1 Extending the SSP scenarios beyond 2100 46

2.4.2 Calibration of country-specific damage functions 48

3 Supplementary results **51**

3.1 Climate model comparison 51

3.2 Sensitivity analysis on SCC 54

3.2.1 Permafrost carbon feedback 54

3.2.2 Ocean methane hydrates 55

3.2.3 Slowdown of the Atlantic Meridional Overturning Circulation 57

3.2.4 Emissions/socio-economic scenario 59

3.2.5 Levels versus growth damages 61

3.2.6 Pure rate of time preference 63

3.2.7 Elasticity of marginal utility of consumption 65

3.2.8 Non-market damages 66

3.2.9 Estimating a confidence interval for the effect of tipping points on the SCC 68

3.2.10 Savings rates 69

3.3 Spatial impacts of tipping points 72

3.4 Effect of tipping points on inequality 77

3.5 Effect of tipping points on warming and sea level rise 78

3.6 Analysis of Monte Carlo sample size and trimming the SCC distribution . . 86

1 Literature review

We sought to collect all the papers published in climate change economics (either in monographs, edited volumes, journals, or as working papers) that fulfil two criteria: (i) model a tipping point in the climate system and (ii) evaluate the economic consequences of that tipping point (as opposed to the purely environmental/geophysical consequences). We associate climate tipping points with the well-known definition of 'tipping elements' as "subsystems of the Earth system that are at least subcontinental in scale and can be switched – under certain circumstances – into a qualitatively different state by small perturbations" (1). This broad search yielded 52 articles, listed in Table 1. The cut-off date for our literature search was 2019.

We then reduced the list by adding a further criterion: (iii) the presence of geophysical foundations. This yielded 21 articles with what we regard as plausible geophysical dynamics. This third criterion is admittedly the most subjective to apply, which is reflected in an additional 4 articles that do contain a geophysical component, albeit still stylized in some key way (marked 'stylized/geophysical' in the table). Our selection criterion for geophysical realism is best illustrated by two examples – both from the same scholar – that together characterise the spectrum (see the simple schematic below). On the *ad hoc* end of the spectrum are arbitrary changes to aggregated damage functions in Integrated Assessment Models (IAMs), which map the increase in global mean temperature into welfare-equivalent losses in global GDP per capita. For example, in *The Climate Casino*, Nordhaus suggested including tipping points by assuming a "stylized tipping-point damage function", according to which damages increase sharply at warming of 3.5°C and become prohibitive beyond 4.5°C (2). In Nordhaus' own words: "These assumptions are at the outer limit of what seems plausible and have no solid basis in empirical estimates of damages" (p213) .

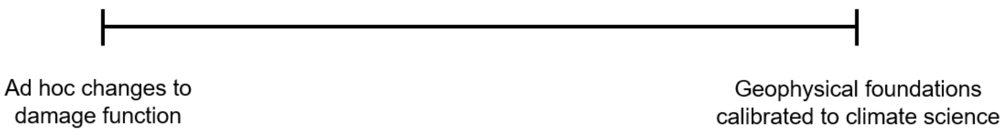

Table 1: Climate-economic tipping points studies included in present analysis

Paper	Integrated assessment model	Tipping point (TP)	TP module
Anthoff, Estrada, Tol (*AER P&P*, 2016)(6)	FUND version 4.0	AMOC	Geophysical
Azar, Lindgren (*Climatic Change*, 2003)(7)	DICE	Inspired by AMOC and WAIS collapse	Stylized
Bahn et al. (*Energy Policy*, 2011)(8)	MERGE5	AMOC	Geophysical
Baranzini, Chesney, Morriset (*Energy Policy*, 2003)(9)	Cline (1992)	WAIS collapse and AMOC	Stylized
Belaia (Dissertation, 2017)(10)	RICE-ISM-AD	ISM	Geophysical
Belaia, Funke, Glanemann (*ERE*, 2017)(11)	DICE-CJL	AMOC	Geophysical
Berger, Emmerling, Tavoni (*Mgt Sci*, 2016)(12)	DICE adapted	AMOC	Stylized
Bickel (*Env. Systems & Decisions*, 2013)(13)	DICE 2007	Not specified	Stylized
Cai et al. (*PNAS*, 2015)(14)	DSICE (based on DICE07)	Not specified	Stylized
Cai, Lenton, Lontzek (*NCC*, 2016)(15)	DSICE (based on DICE-2013R)	5 TPs: AMOC, GIS, WAIS, AMAZ, ENSO	Stylized/geophysical
Cai, Lontzek (*JPE*, 2019)(16)	DSICE (based on DICE07)	AMOC, GIS, WAIS, AMAZ, ENSO	Stylized
Cai, Brock, Xepepadeas (Working paper, 2016)(17)	extends DSICE model of Cai et al. 2015	AMOC	Stylized/geophysical
Ceronsky, Anthoff, Hepburn, Tol (Working paper, 2011)(18)	FUND version 3.6	AMOC, OMH	Geophysical
Chao (*Risk Analysis*, 1995)(19)	unique to this paper	Inspired by WAIS collapse inter alia	Stylized
Diaz and Keller (*AER P&P*, 2016)(20)	DICE - WAIS	Potential WAIS collapse	Geophysical
Dumas and Ha-Duong (Book chapter, 2005)(21)	DIAM 2.3	Inspired by AMOC	Stylized
Engstrom, Gars (*ERE*, 2016)(22)	Golosov et al. (2014)	3 TPs: damages, CO_2 removal; PCF	Stylized
Gjerde, Grepperud and Kverndokk (*REE*, 1999)(23)	from Kverndokk (1994)	Inspired by WAIS collapse, AMOC, PCF	Stylized
González-Eguino et al. (*Earth's Future*, 2017)(24)	DICE 2013R	SAF-inspired	Geophysical
Guillerminet, Tol (*Climatic Change*, 2008)(25)	n/a	WAIS collapse	Stylized
Heutel, Moreno-Cruz, Shayegh (*JEBO*, 2016)(26)	DICE 2007	3 TPs: climate feedback, carbon sink, economic loss (pre- and post-climate policy)	Stylized
Hope, Schaefer (*NCC*, 2016)(27)	PAGE09	PCF	Geophysical
Keller et al. (*Climatic Change*, 2000)(28)	DICE 1994	AMOC	Geophysical
Keller, Bolker, Bradford (*JEEM*, 2004)(29)	DICE94	AMOC	Geophysical
Kessler (*Climate Change Economics*, 2017)(30)	DICE-2013R	PCF	Geophysical
Lamperti et al. (*Ecological Economics*, 2018)(31)	Dystopian Schumpeter meeting Keynes (DSK)	Not applicable	Stylized
Lempert, Sanstad, Schlesinger (*Energy Economics*, 2006)(32)	DICE94	AMOC	Stylized
Lemoine, Traeger (*AEJ:Pol*, 2014)(33)	4-stated DICE (based on DICE07)	Jump in ECS, drop in CO_2 removal	Stylized
Lemoine, Traeger (*NCC*, 2016)(34)	4-stated DICE (based on DICE07)	Jump in equilibrium climate sensitivity; fall in CO_2 removal; damages	Stylized
Lemoine, Traeger (*JEBO*, 2016)(35)	4-stated DICE (based on DICE07)	Jump in ECS, drop in CO_2 removal	Stylized
Link and Tol (*Port Econ J*, 2004)(36)	FUND version 2.8	AMOC	Geophysical
Link and Tol (*Climatic Change*, 2011)(37)	FUND version 2.8n	AMOC	Geophysical
Lontzek, Narita, Wilms (*ERE*, 2016)(38)	n/a	Tropical and boreal forest dieback	Geophysical
Lontzek et al. (*NCC*, 2015)(39)	"DSICE" (based on DICE07)	AMOC, GIS, WAIS, AMAZ, ENSO	Stylized/geophysical
McInerney, Lempert, Keller (*Climatic Change*, 2012)(40)	DICE-07	AMOC	Stylized
Naevdal (*JEDC*, 2006)(41)	n/a	WAIS	Stylized
Naevdal, Oppenheimer (*REE*, 2007)(42)	n/a	AMOC	Stylized
Nicholls, Tol, Vafeidis (*Climatic Change*, 2008)(43)	FUND version 2.8n	WAIS	Geophysical
Nordhaus (Book chapter, 1994)(44)	DICE-94	Inspired by WAIS, AMOC; PCF, etc.	Stylized
Nordhaus (*PNAS*, 2019)(3)	DICE16R2-GIS	GIS	Geophysical
Nordin (Dissertation, 2014)(45)	DICE2013	GIS, WAIS, AMAZ, PCF, OMH	Stylizedl
Peck, Teisberg (*Climatic Change*, 1995)(46)	CETA-R	None specified	Stylized
Pycroft, Vergano, Hope (*Global Environmental Change*, 2014)(47)	PAGE09	Extreme sea-level rise from GIS and WAIS	Stylized/geophysical
Schlesinger et al. (Book chapter, 2006)(48)	DICE99	AMOC	Geophysical
Shayegh, Thomas (*Climatic Change*, 2015)(49)	DICE 2007	Climate sensitivity	Stylized
Sims, Finoff (*JAERE*, 2017)(50)	n/a	Ice sheet collapse, special case	Stylized
van der Ploeg (*EER*, 2014)(51)	n/a	OMH	Stylized
van der Ploeg, de Zeeuw (*JEEA*, 2017)(52)	n/a	None specified	Stylized
Whiteman, Hope and Wadhams (*Nature*, 2013)(53)	PAGE09	OMH (Arctic)	Geophysical
Wirths, Rathmann, Michaelis (*EEPS*, 2018)(54)	DICE 2013R with PCF	PCF	Geophysical
Yohe (*Global Environmental Change*, 1996)(55)	CONN	Change in equilibrium climate sensitivity	Stylized
Yohe, Schlesinger, Andronova (*Integrated Assessment Journal*, 2006)(56)	DICE99 adding a simple ATHC model	AMOC	Geophysical
Yumashev, et al. (*Nature Comms*, 2019)(57)	PAGE-ICE	PCF, SAF	Geophysical

Notes: PCF - permafrost carbon feedback; OMH - dissociation of ocean methane hydrates / clathrates; SAF - surface albedo feedback / arctic sea ice; AMAZ - Amazon rainforest dieback; GIS - Greenland ice sheet disintegration; WAIS - West Antarctic ice sheet disintegration; AMOC - Atlantic meridional overturning circulation slowdown; ISM - Indian summer monsoon variability; ECS - equilibrium climate sensitivity.

At the *geophysical* end of the spectrum, Nordhaus has recently incorporated a simple, tractable model of disintegration of the Greenland Ice Sheet (GIS) in his DICE IAM (3). The GIS module is calibrated on results from the underlying literature on ice-sheet dynamics, principally (4). Damages are calibrated on a detailed study of the relationship between sea level rise, coastal defence costs and the costs of coastal flooding and permanent inundation (5). The result is a more realistic, yet tractable climate-economy IAM.

Figure 1 shows that economic studies into climate tipping points date back to at least the mid-1990s, with the first paper incorporating geophysical realism appearing around the turn of the millennium (28).

Figure 1: Count of papers identified in the literature review by year of publication

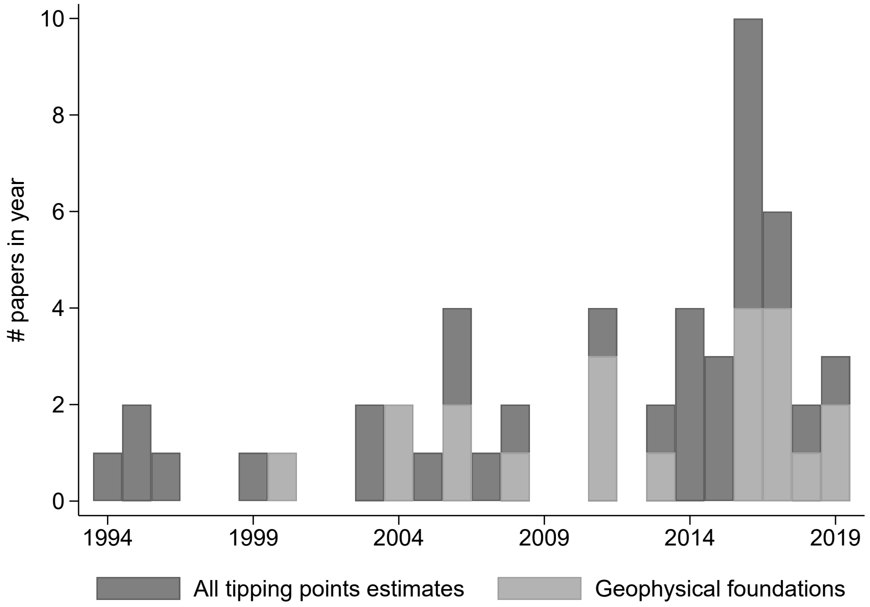

Figure 2 shows that, while a variety of IAMs have been deployed, the clear majority of those studies aiming for geophysical realism have been based on a version of the three most popular IAMs: DICE, FUND, and PAGE. DICE has been the overwhelming favourite.

Figure 2: Count of papers identified in the literature review, grouped by the IAM used

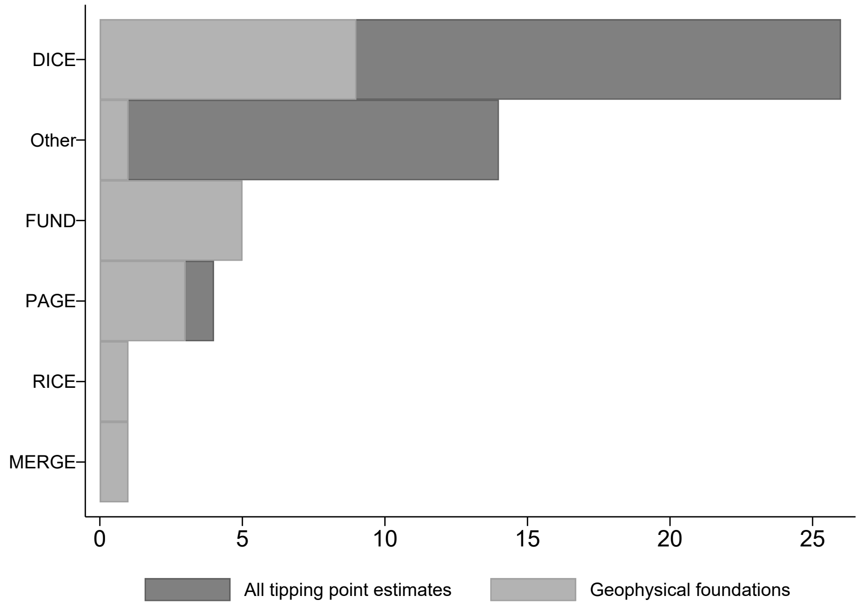

Figure 3 shows the interdisciplinary nature of this literature, which includes publications in journals in general-interest economics, multi-disciplinary science, as well as environmental economics and environmental studies.

Figure 3: Count of papers identified in the literature review grouped by the journal/outlet in which they were published. List of acroynms: NCC = Nature Climate Change; ERE = Environmental and Resource Economics; REE = Resource and Energy Economics; PNAS = Proceedings of the National Academy of Sciences; JEBO = Journal of Economic Behavior and Organization; GEC = Global Environmental Change; AER P&P = American Economic Review, Papers and Proceedings; JPE = Journal of Political Economy; JEEM = Journal of Environmental Economics and Management; JEEA = Journal of the European Economic Association; JEDC = Journal of Economic Dynamics and Control; JAERE = Journal of the Association of Environmental and Resource Economists; EER = European Economic Review; AEJ = American Economic Journal.

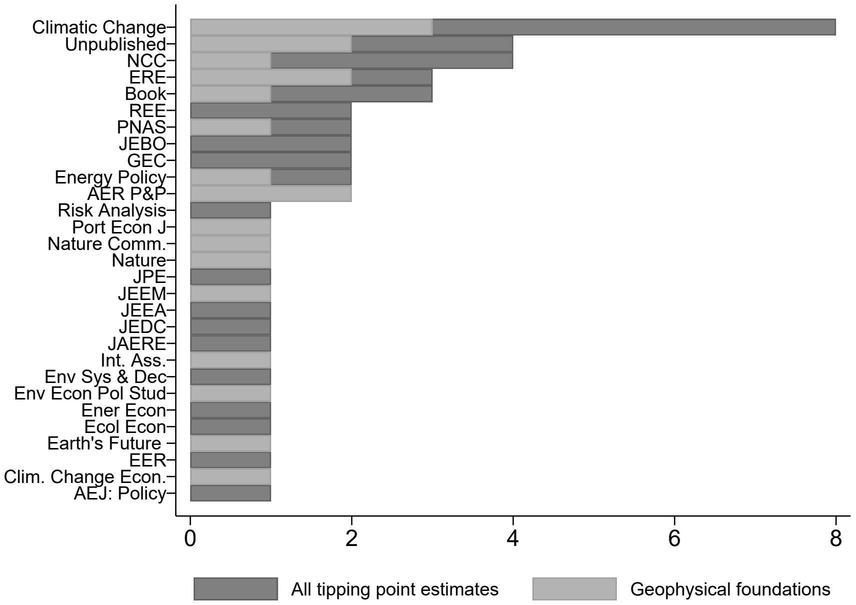

2 Model description

This section presents a detailed description of the META (Model for Economic Tipping point Analysis) model, which is publicly available at https://github.com/openmodels/META-2021. Figures 4 and 5 provide an overview of the model structure. Figure 4 provides a schematic diagram of the climate module. The inputs to the climate module are exogenous greenhouse

gas emissions; the output is the change in global mean surface temperature (GMST). Three tipping points provide positive feedbacks from the increase in GMST to greenhouse gas emissions (the permafrost carbon feedback, dissociation of ocean methane hydrates, and Amazon rainforest dieback), while one provides a positive feedback from the increase in GMST to radiative forcing (Arctic sea-ice loss/surface albedo feedback).

Figure 5 provides a schematic diagram of the damages/economic module. The input to the damages/economic module is the change in GMST from the climate module. The output is discounted utility/social welfare. Slowdown of the Atlantic Meridional Overturning Circulation modulates the relationship between global and national mean temperature change. Disintegration of the Greenland and West Antarctic Ice Sheets increases sea level rise. Variability of the Indian Summer Monsoon directly impacts GDP in India due to droughts and floods.

Figure 4: Schematic diagram of the climate module. Blue boxes indicate variables; yellow boxes indicate tipping point modules; orange boxes indicate other modules.

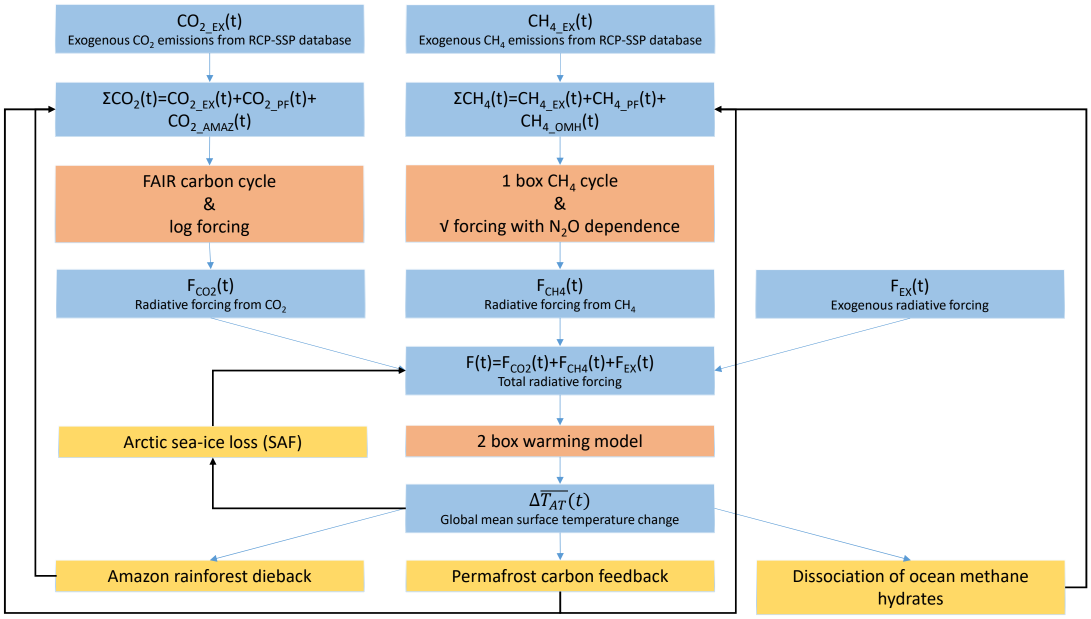

Figure 5: Schematic diagram of the damages/economic module. Blue boxes indicate variables; yellow boxes indicate tipping point modules; orange boxes indicate other modules.

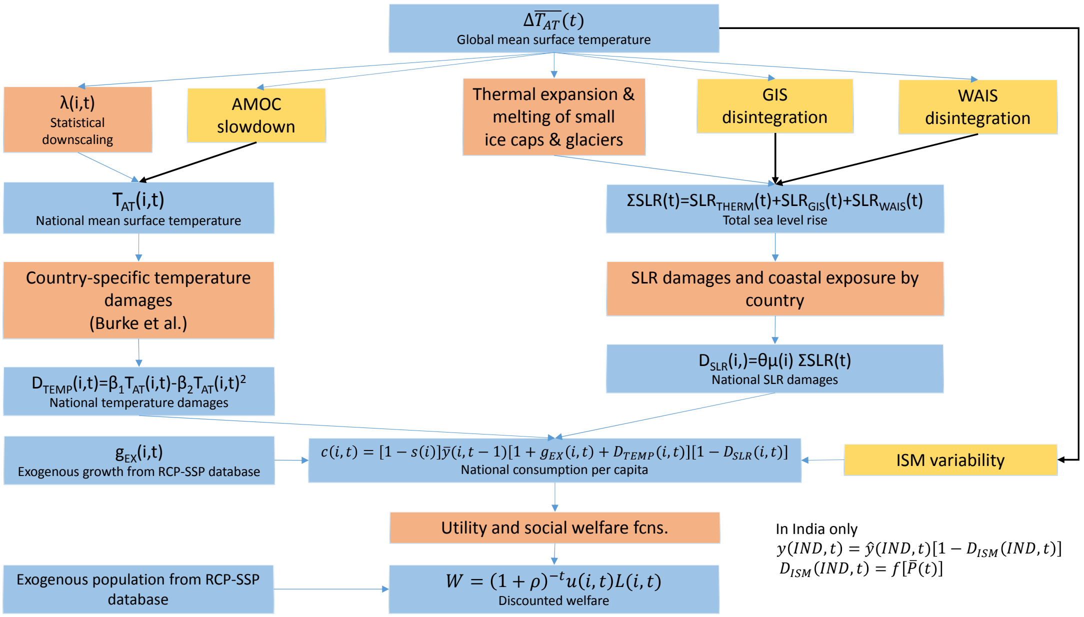

2.1 Tipping point modules

2.1.1 Permafrost carbon feedback

Our model of the permafrost carbon feedback (PCF) is taken from Kessler (30). This is a tractable model that mimics in reduced form the physical-science literature quantifying permafrost carbon release by simulating two stages: (i) permafrost thaw as a function of rising temperatures and (ii) decomposition of thawed permafrost, leading to the release of CO_2 or CH_4. Kessler built the model for incorporation in DICE and, although we don't use DICE, the level of abstraction from the underlying physical processes is well suited to our approach. Despite the level of abstraction, however, the model retains enough structure to be directly calibrated on estimates reported in the underlying literature.

In the first stage, near-surface permafrost thaw is a linear function of warming relative

to time zero:

$$\mathrm{PF}_{\mathrm{extent}}(t) = 1 - \beta_{\mathrm{PF}} \left[\Delta\overline{T_{\mathrm{AT}}}(t) - \Delta\overline{T_{\mathrm{AT}}}(0) \right], \tag{1}$$

where $\mathrm{PF}_{\mathrm{extent}}(t) \equiv \mathrm{PF}_{\mathrm{area}}(t)/\mathrm{PF}_{\mathrm{area}}(0)$, i.e. $\mathrm{PF}_{\mathrm{extent}}(t)$ is the area of permafrost remaining at time t relative to time zero, $\Delta\overline{T_{\mathrm{AT}}}$ is the global mean surface air temperature relative to pre-industrial, and β_{PF} is a coefficient representing the sensitivity of permafrost thaw to temperature, which Kessler calibrated by regressing estimates of thaw on temperature from the literature. $t = 0$ in our model is the year 2010.

The amount of carbon in freshly thawed permafrost at time t, C_{thawedPF}, is then the product of the total stock of carbon locked in the near-surface northern circumpolar permafrost region, C_{PF}, and the area of permafrost freshly thawed:

$$C_{\mathrm{thawedPF}}(t) = -C_{\mathrm{PF}} \left[\mathrm{PF}_{\mathrm{extent}}(t) - \mathrm{PF}_{\mathrm{extent}}(t-1) \right]. \tag{2}$$

Once thawed, the principal way in which carbon is released to the atmosphere is microbial decomposition and this happens slowly. Some of the carbon is released as CO_2 and some as CH_4. Kessler's model divides the stock of thawed carbon into a passive reservoir that releases no carbon and an active reservoir that decomposes exponentially and releases CO_2 and CH_4 in fixed proportion. Therefore cumulative CO_2 emissions to the atmosphere from thawed permafrost, $\mathrm{CCum}_{\mathrm{PF}}$, are given by

$$\mathrm{CCum}_{\mathrm{PF}}(t) = \sum_{s=0}^{t} C_{\mathrm{thawedPF}}(s) \left(1 - \mathrm{propPassive}\right) \left(1 - \exp^{\frac{-t-s}{\tau}}\right), \tag{3}$$

where propPassive is the proportion of thawed permafrost in the passive reservoir and τ is the e-folding time of permafrost decomposition in the active reservoir, which is multiple decades (see below). The fluxes of CO_2 and CH_4 are respectively given by

Table 2: PCF model parameter values

	Kessler main spec.	Lower/upper bounds	Fit of Hope and Schaefer (2016) (27)	Fit of Yumashev et al. (2019) (57)
β	0.172	0/1	0.066	0.085
C_{PF} (GtC)	1035	885/1185	1160	1066
propPassive	0.40	0.29/0.51	0.37	0.41
τ (years)	70	0/200	31	66

$$\mathrm{CO}_{2\_\mathrm{PF}}(t) = (1 - \mathrm{propCH}_4)\left[\mathrm{CCum}_{\mathrm{PF}}(t) - \mathrm{CCum}_{\mathrm{PF}}(t-1)\right], \quad (4)$$

$$\mathrm{CH}_{4\_\mathrm{PF}}(t) = (\mathrm{propCH}_4)\left[\mathrm{CCum}_{\mathrm{PF}}(t) - \mathrm{CCum}_{\mathrm{PF}}(t-1)\right], \quad (5)$$

where propCH_4 is the share of CH_4 emissions in total carbon emissions.

We can directly reproduce the permafrost carbon emissions estimated by (30) just by imputing her reported parameter values for β_{PF}, C_{PF}, propPassive, τ and propCH_4 into Equations (1)-(5). In addition, we use this model to fit the results of the two other papers contributed to the IAM literature on the PCF, namely Hope and Schaefer (27) and Yumashev et al. (57). (27) coupled the PAGE09 IAM to the SiBCASA model of the PCF. (57) developed a new version of the PAGE IAM called PAGE-ICE, which includes a representation of the PCF calibrated both on SiBCASA and another PCF model called JULES. We first obtain estimates of permafrost CO_2 emissions from each paper as a function of temperature, and then minimise the sum of squared residuals between these papers' estimates and estimates from Kessler's model, using four of the free parameters in Kessler's model, i.e. β_{PF}, C_{PF}, propPassive, and τ, each parameter restricted to lie within physically plausible bounds. Table 2 reports the various parameter values. Figure 6 shows the fit to cumulative CO_2 emissions from (27) and (57). CH_4 emissions for these two papers are obtained simply by using the fitted parameters in combination with the fixed value of propCH_4 from (30).

Figure 6: Fit of cumulative permafrost CO_2 emissions from Hope and Schaefer (2016) (27), top panel, and Yumashev et al. (2019) (57), bottom panel

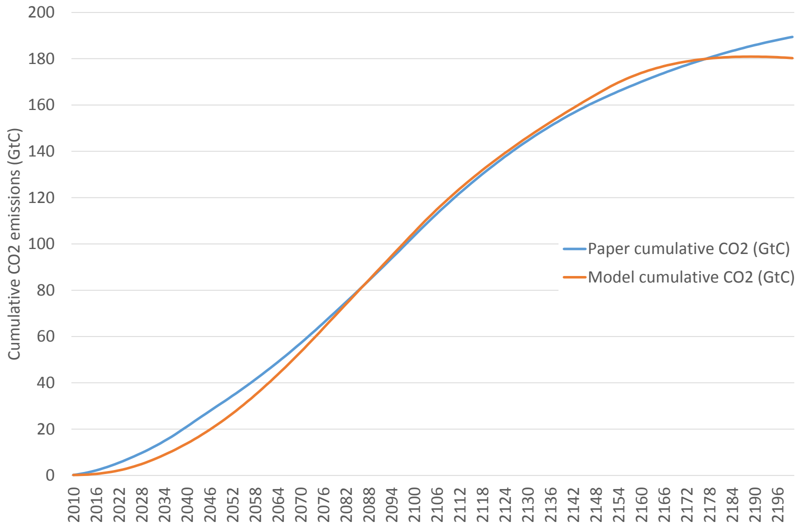

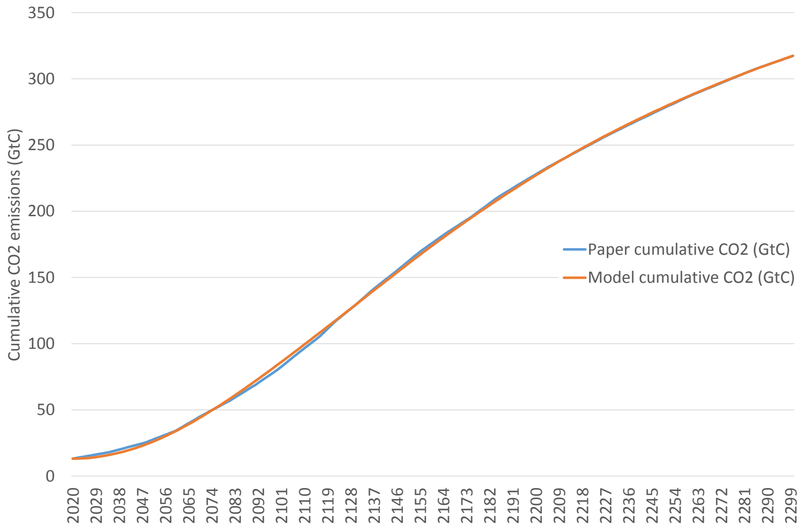

2.1.2 Ocean methane hydrates

There have been two studies of the economic cost of destabilization of ocean methane clathrates/hydrates. The first is Whiteman et al. (53), who implemented what-if scenarios in PAGE09, releasing a pulse of CH_4 emissions of fixed size and duration into the model at a given point in time. These scenarios were based on the work of (58) on hydrates locked within subsea permafrost on the East Siberian Arctic shelf. (53) implemented alternative scenarios. Most of their scenarios involved injecting 50GtCH_4 in total over periods of 10 to 30 years, starting at different times from 2015 to 2035.[1] The other study is Ceronsky et al. (59). They implemented three what-if scenarios, in which pulses of CH_4 emissions from the reservoir of CH_4 distributed globally on continental shelves and slopes were released in the FUND IAM. These emissions pulses all commence in 2050 and comprise permanent flows of 0.2GtCH_4 per year, 1.784GtCH_4/yr and 7.8GtCH_4/yr respectively.

In order to incorporate these studies in our analysis, their what-if scenarios need to be assigned probabilities. To do this, we use the framework of survival analysis, treating each emissions pulse as a hazard event and assigning it a hazard rate, i.e. the conditional probability that the event will occur in a particular year, given the temperature in that year and that the event has not occurred previously. This is both convenient and conforms with the way some of the other studies we synthesise treat tipping points, e.g. on Amazon rainforest dieback (60) and disintegration of the West Antarctic Ice Sheet (61) (see below). Once triggered, each CH_4 emissions pulse of given size lasts its pre-specified amount of time. In general, we can write the flow of CH_4 emissions from dissociation of ocean methane hydrates at time t, $CH_{4\_OMH}(t)$, as

$$CH_{4\_OMH}(t) = \left(\frac{\overline{CH_{4\_OMH}}}{\Delta_{OMH}}\right) I_{OMH}(t) \iff \sum_{s=0}^{t-1} CH_{4\_OMH}(s) < \overline{CH_{4\_OMH}}, \tag{6}$$

$$CH_{4\_OMH}(t) = 0 \iff \sum_{s=0}^{t-1} CH_{4\_OMH}(s) = \overline{CH_{4\_OMH}}, \tag{7}$$

[1] They also injected a smaller pulse of 25GtCH_4 between 2015 and 2025 in one scenario.

where $\overline{\text{CH}_{4\_\text{OMH}}}$ is the pre-specified total amount of methane released, e.g. 50Gt in the case of the main specification of (53), and Δ_{OMH} is the duration of the release, e.g. 10 years. Applying this formalism to (59), $\overline{\text{CH}_{4\_\text{OMH}}}/\Delta_{\text{OMH}} \in \{0.2, 1.784, 7.8\}$ and total CH_4 released from ocean CH_4 hydrates is bounded only by the product of $\overline{\text{CH}_{4\_\text{OMH}}}/\Delta_{\text{OMH}}$ and the model horizon, i.e. the inequality constraint in Equations (6) and (7) does not bind. $I_{\text{OMH}}(t)$ is an indicator function taking a value of zero before the hazard event is triggered and one thereafter. In general, its transition function is

$$I_{\text{OMH}}(t) = f\left[I_{\text{OMH}}(t-1), \Delta\overline{T_{\text{AT}}}(t), \varepsilon(t)\right], \tag{8}$$

where $\varepsilon(t)$ is an i.i.d. random shock. That is, in each period the value of I_{OMH} depends on its own value in the previous period, the current atmospheric temperature, and the random shock. Specifically, the probability transition matrix for $I_{\text{OMH}}(t)$ is

$$\begin{bmatrix} 1 - p_{\text{OMH}}(t) & p_{\text{OMH}}(t) \\ 0 & 1 \end{bmatrix}, \tag{9}$$

where $p_{\text{OMH}}(t)$ is the probability that the CH_4 emissions pulse is triggered in year t. This is given by

$$p_{\text{OMH}}(t) = 1 - \exp\left[-b_{\text{OMH}}\Delta\overline{T_{\text{AT}}}(t)\right], \tag{10}$$

where b_{OMH} is the hazard rate.

In order to calibrate the hazard rate, we use the study of Archer et al. (62), which presents a global model of CH_4 hydrates on continental shelves and slopes and the release of CH_4 as temperatures rise. Their study shows the sensitive dependence of ocean CH_4 release on a critical bubble volume fraction threshold. That is, when ocean CH_4 hydrates melt, it is uncertain whether the CH_4 escapes the ocean sediment into the ocean.[2] Colder

[2]There is further uncertainty about whether the CH_4 that reaches the ocean bottom eventually escapes into the atmosphere (it depends on aerobic oxidation of CH_4 by bacteria in the water column), however this uncertainty is thought to be smaller.

temperatures closer to the sea floor and chemical reactions (anaerobic oxidation by bacteria and archaea) both effectively trap the CH_4 from escaping. The more CH_4 is in bubbles, however, the more likely it is to escape. In the model of (62), the bubble volume upon melting of the hydrates must exceed the critical bubble volume fraction in order for the CH_4 to be released. Calibrating the hazard rate on (62) means that we re-interpret (53) in the context of the global reservoir of CH_4 hydrates on continental shelves and slopes, rather than the reservoir of CH_4 locked in subsea permafrost in the Arctic region. This is justified, since other research suggests a large release of CH_4 from the Arctic subsea permafrost within the next two centuries is extremely unlikely (63).[3]

According to (62), cumulative CH_4 released in very long-run equilibrium upon 1°C warming varies hugely from about 10GtCH_4 to 541GtCH_4 for critical bubble fractions of 10% and 1% respectively.[4] Upon 3°C warming the range increases to about 32-1084Gt. Moreover (62) report that there is next to no empirical evidence on the critical bubble fraction. In the absence of such evidence, we try three alternative specifications of the probability distribution of equilibrium cumulative CH_4 release as a function of the critical bubble fraction (Table 3). The uniform distribution is an application of the principle of insufficient reason. The triangular and especially the beta distribution are more conservative in the sense of assigning more probability mass to higher critical bubble fractions and in turn lower equilibrium CH_4 releases.

Irrespective of the critical bubble fraction, CH_4 released from melting ocean hydrates is thought to take a very long time to reach the atmosphere, much longer than permafrost carbon. Therefore, in order to convert the equilibrium CH_4 release into a transient release, we conservatively assume a release rate of just 0.2%, implying an e-folding time of 500 years and approximately 3,000 years for equilibrium to be reached (also see 62).

To give an example of how we then calibrate the hazard rate b_{OMH}, we use a middle

[3] Indeed, the scenarios in (53) were criticised at the time of publication for being unrealistic in the context of Arctic subsea processes; see *Nature* volume 300, p529.

[4] Based on digitising Figure 7 in their paper.

Table 3: Calibration of OMH hazard rate, b_{OMH}. Triangular distribution assumes modal critical bubble fraction of 10%, supports of 1% and zero CH_4 release. Beta distribution assigns cumulative probabilities of 0.67, 0.9, 0.95, 0.99 and 1 to critical bubble fractions of 10%, 7.5%, 5%, 2.5% and 1% respectively.

		uniform	triangular	beta
Whiteman et al. (53) 50GtCH_4 by 2035	p_{OMH}	95.3%	90.2%	24.4%
	b_{OMH}	1.290	0.977	0.118
Whiteman et al. (53) 50GtCH_4 by 2025	p_{OMH}	86.4%	8.9%	12.0%
	b_{OMH}	1.457	0.068	0.093
Whiteman et al. (53) 50GtCH_4 by 2045	p_{OMH}	97.7%	97.8%	33.0%
	b_{OMH}	1.691	1.712	0.178
Ceronsky et al. (59) 0.2GtCH_4/yr 2050-2200	p_{OMH}	100%	100%	67.1%
	b_{OMH}	2.577	3.987	0.365
Ceronsky et al. (59) 1.784GtCH_4/yr 2050-2200	p_{OMH}	99.7%	100%	52.4%
	b_{OMH}	1.858	2.550	0.244
Ceronsky et al. (59) 7.8GtCH_4/yr 2050-2200	p_{OMH}	98.5%	99.2%	39.1%
	b_{OMH}	1.374	1.581	0.1634

scenario from (53) of a cumulative release of 50GtCH_4 over 20 years from 2015 to 2035. According to the mid-range RCP4.5 scenario of the Intergovernmental Panel on Climate Change (IPCC), fed into our climate module excluding tipping points, GMST in 2035 will be about 1.6°C above pre-industrial. Using the approach just described to represent the modelling results of (62), we estimate a 24.4% probability of a 50GtCH_4 cumulative release by 2035 assuming a beta distribution. This gives $b_{\text{OMH}} = 0.118$. We follow the same procedure to assign hazard rates using the uniform and triangular distributions, and apply it to different durations of emissions pulse investigated by (53), as well as the scenarios in (59). Table 3 reports all the estimated hazard rates. We prefer the beta distributions except in sensitivity analysis, as they are more conservative.

2.1.3 Amazon rainforest dieback

Dieback of the Amazon rainforest was included in the study of Cai et al. (60) as a carbon-cycle feedback. This is the study we incorporate in our analysis. Naturally a wide range of other

economically important consequences of Amazon rainforest dieback are thereby excluded, including those on biodiversity and ecosystems. These have yet to be incorporated in any economic modelling study, to the best of our knowledge.

As mentioned above, (60) model tipping points through survival analysis. In the case of Amazon rainforest dieback, 50GtC is released over 50 years upon triggering the hazard event. Using parallel formalism to ocean methane hydrates, CO_2 emissions from Amazon rainforest dieback at time t, $CO_{2\_AMAZ}(t)$, are given by

$$CO_{2\_AMAZ}(t) = \left(\frac{\overline{CO_{2\_AMAZ}}}{\Delta_{AMAZ}}\right) I_{AMAZ}(t) \iff \sum_{s=0}^{t-1} CO_{2\_AMAZ}(s) < \overline{CO_{2\_AMAZ}}, \quad (11)$$

$$CO_{2\_AMAZ}(t) = 0 \iff \sum_{s=0}^{t-1} CO_{2\_AMAZ}(s) = \overline{CO_{2\_AMAZ}}, \quad (12)$$

where $\overline{CO_{2_AMAZ}} = 50$GtC and $\Delta_{AMAZ} = 50$ years. The probability of the indicator function $I_{AMAZ}(t)$ transitioning from zero to one is

$$p_{AMAZ}(t) = 1 - \exp\left[-b_{AMAZ}\Delta\overline{T_{AT}}(t) - 1\right], \quad (13)$$

where the hazard rate $b_{AMAZ} = 0.00163$ in (60) is taken from the expert elicitation study of (64).

2.1.4 Greenland Ice Sheet

Our model of disintegration of the Greenland Ice Sheet (GIS) is based on (3), which follows an approach conceptually similar to Kessler's (30) PCF model by building a simple, reduced-form process model of GIS disintegration for incorporation in DICE.[5] The GIS model is calibrated on results from the underlying literature modelling ice-sheet dynamics. At the heart of the GIS model is the very long-run equilibrium relationship between atmospheric

[5]The resulting model is called DICE-GIS and builds on DICE-2016R2.

temperature and the volume of the GIS. Assuming this is reversible, (3) specified

$$\overline{\Delta T^{*}_{\mathrm{GIS}}}(t) = \Delta \overline{T_{\mathrm{GIS\_MAX}}} \left[1 - V_{\mathrm{GIS}}(t)\right], \tag{14}$$

where $\Delta \overline{T^{*}_{\mathrm{GIS}}}(t)$ is defined as the atmospheric temperature increase relative to initial temperature that is associated with a particular degree of melting of the GIS in equilibrium and $V_{\mathrm{GIS}}(t) \in [0,1]$ is the volume of the GIS expressed as a fraction of the initial volume.[6] In Nordhaus' main specification, Eq. (14) was calibrated on paleoclimatic data from (65), which gives $\Delta \overline{T_{\mathrm{GIS\_MAX}}} = 3.4$ and implies that the GIS is fully melted in equilibrium when the global mean surface temperature is 3.4°C above pre-industrial. If Robinson et al. (4) is used for calibration instead, $\Delta \overline{T_{\mathrm{GIS\_MAX}}} = 1.8$.[7] An alternative, cubic specification of the equilibrium temperature-volume relationship allows for hysteretic behaviour. Fitted on (65), this is given by

$$\Delta \overline{T^{*}_{\mathrm{GIS}}}(t) = \Delta \overline{T_{\mathrm{GIS\_MAX}}} - 20.51 V_{\mathrm{GIS}}(t) + 51.9 \left[V_{\mathrm{GIS}}(t)\right]^{2} - 34.79 \left[V_{\mathrm{GIS}}(t)\right]^{3}. \tag{15}$$

Nordhaus (3) showed that the change in specification makes little difference on the optimal emissions path, which involves relatively limited warming, but can make a difference on high-emissions scenarios.

The difference equation for $V_{\mathrm{GIS}}(t)$, i.e. the GIS melt rate, can be written as

$$\begin{aligned} V_{\mathrm{GIS}}(t) - V_{\mathrm{GIS}}(t-1) \;=\; & \beta_{GIS} \mathrm{sgn}\left[\Delta \overline{T_{\mathrm{AT}}}(t-1) - \Delta \overline{T^{*}_{\mathrm{GIS}}}(t-1)\right] \times \\ & \times \left[\Delta \overline{T_{\mathrm{AT}}}(t-1) - \Delta \overline{T^{*}_{\mathrm{GIS}}}(t-1)\right]^{2} V_{\mathrm{GIS}}(t-1)^{0.2}, \end{aligned} \tag{16}$$

where $\beta_{GIS} = -0.0000106$ based on regression analysis of estimates from (4).[8] The basic idea embodied in Eq. (16) is that melting of the GIS depends on the difference between the

[6](3) also reports runs in which $T^{*}_{\mathrm{GIS}}(t) = T_{\mathrm{GIS\_MAX}} \left[1 - V_{\mathrm{GIS}}(t)\right]^{0.5}$ and finds the results are very similar.

[7]Noting that the melt rate coefficient β_{GIS} below also needs to be recalibrated to -0.0000088 to fit (4).

[8]This corresponds with Nordhaus' (3) reported value per five years divided by 5 to bring it into line with our annual time step, then divided by 100 given that we define $V_{\mathrm{GIS}}(t)$ as a fraction.

actual atmospheric temperature and the equilibrium GIS temperature, as well as the volume of the GIS at the time.

Sea level rises linearly in response to GIS melt,

$$SLR_{\mathrm{GIS}}(t) = 7\left[1 - V_{\mathrm{GIS}}(t)\right], \tag{17}$$

where SLR_{GIS} is defined relative to the year 2000. This implies that complete disintegration of the GIS would increase global mean sea level by 7 metres.

2.1.5 West Antarctic Ice Sheet

Disintegration of the West Antarctic Ice Sheet (WAIS) was modelled by Diaz and Keller (61). Like Nordhaus (3), they built a simple model of WAIS melting for incorporation in DICE. Unlike Nordhaus, who focused on a best estimate around which selected sensitivity analysis was performed, (61) used the framework of survival analysis. In particular, global mean sea level rise from WAIS melting, $SLR_{\mathrm{WAIS}}(t)$, is given by

$$SLR_{\mathrm{WAIS}}(t) = \sum_{s=0}^{t} r_{\mathrm{WAIS}} I_{\mathrm{WAIS}}(s), \tag{18}$$

where r_{WAIS} is an exogenous parameter determining the annual contribution to global mean sea level upon triggering disintegration of the ice sheet, assumed lognormally distributed with a mean of 3.3mm/yr and a standard deviation of 1.65mm/yr. This implies it takes on average 1000 years for the WAIS to disintegrate completely after its tipping point is crossed. $I_{\mathrm{WAIS}}(t)$ is the indicator function for WAIS disintegration, whose probability of transitioning from zero to one, conditional on having been zero in year $t-1$, is

$$p_{\mathrm{WAIS}}(t) \quad = \quad \min\left\{ b_{\mathrm{WAIS}} \left[\Delta\overline{T_{\mathrm{AT}}}(t)\right]^2, 1 \right\}. \tag{19}$$

The hazard rate $b_{\mathrm{WAIS}} = 0.0043$ is also based on the expert elicitation exercise of (64).

2.1.6 Arctic sea-ice loss/surface albedo feedback

Changes in global ice and snow cover also affect the surface albedo feedback (SAF), increasing net radiative forcing. While these effects are implicitly captured in the equilibrium climate sensitivity (ECS) parameter in simple climate models, i.e. the steady-state increase in temperature in response to a doubling of the atmospheric CO_2 concentration, doing so assumes that the marginal forcing from an increase in temperature is constant across temperatures. However, as the area of ice and snow diminishes, the marginal response for further increases in temperature decreases. This SAF dynamic has been modelled by Yumashev et al. (57) using PAGE-ICE and we replicate their model here.

(57) use a quadratic fit of the SAF observed across the CMIP5 models, shown in the top panel of Figure 7. This falling SAF curve describes the weakening feedback loop between changes in temperature and changes in albedo. For low levels of warming, the SAF is greater than the constant value represented in the ECS; as sea-ice and land snow diminish, the feedback effect drops. When sea ice and land snow are absent, the SAF effect is zero. The total radiative forcing due to albedo, however, always increases with temperature, and reaches its maximum when sea ice and land snow are absent.

Total SAF forcing is the integral of the SAF feedback effect across the change in temperature, reaching $2.67\,\mathrm{Wm}^{-2}$ at warming of 10°C. The ECS follows a non-linear curve calculated as a function of the ECS in the last period, and accounting for the different level of feedback compared to a constant level. As a consequence, adding the SAF to the base climate model can result in lower warming eventually.

The calculations for the SAF correction are shown below. The principle of the SAF model is to correct temperatures calculated under the process used in PAGE-ICE, so we first reproduce this temperature calculation. Global PAGE-ICE atmospheric temperature is

Figure 7: Variation in surface albedo feedback (SAF) effects as a function of GMST. **Top:** SAF as a function of temperature, in terms of marginal increases in forcing per degree Kelvin. **Middle:** adjusted value of the ECS when SAF forcing is removed. **Bottom:** cumulative forcing from the SAF, as a function of temperature, in Wm^{-2}.

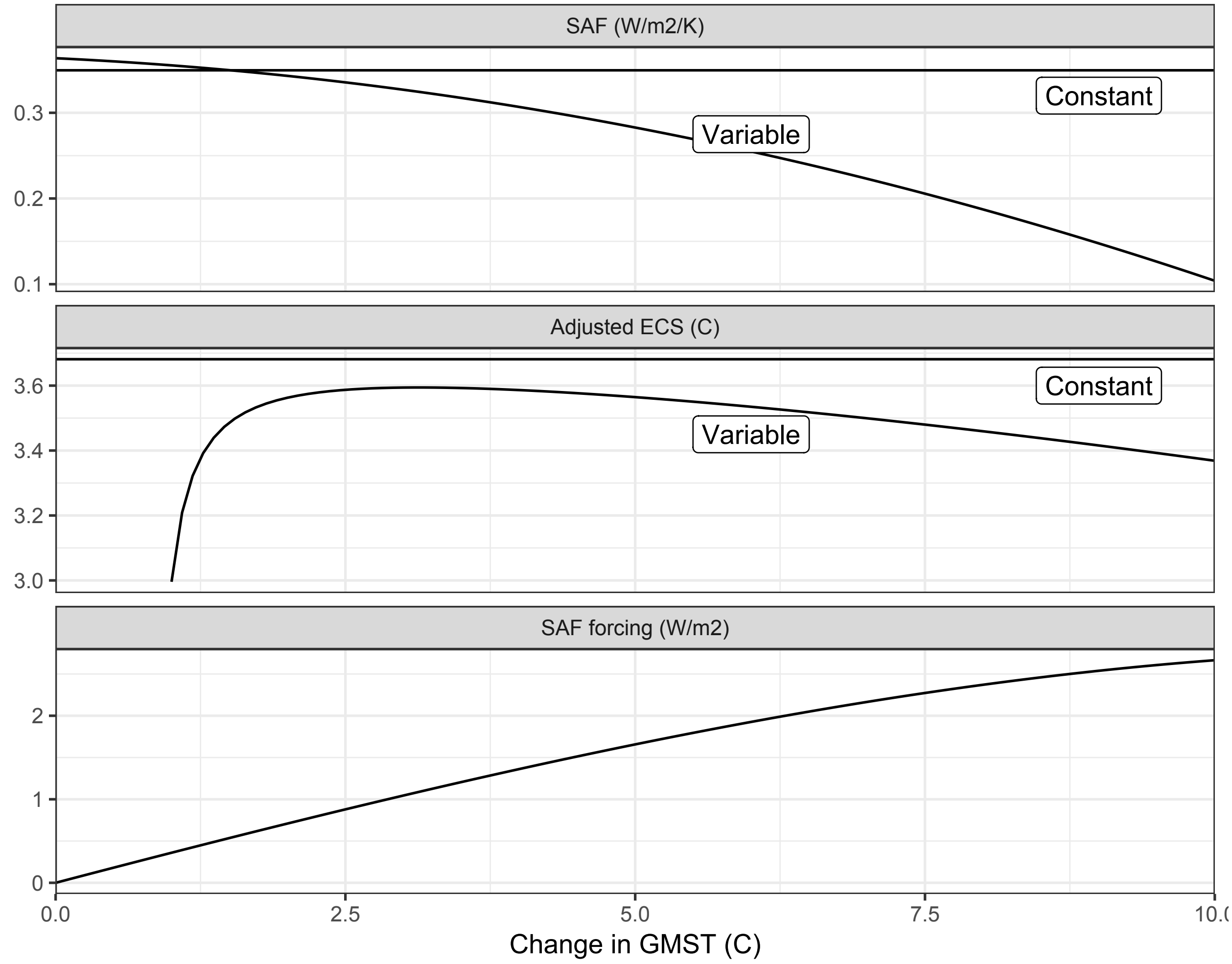

calculated as

$$\begin{aligned}\Delta\overline{T_{ATM-PAGE1}}(t) = &\Delta\overline{T_{ATM-PAGE1}}(t-1) \\ &+ \left(A(t-1) - \text{FRT}B(t-1) - \Delta\overline{T_{ATM-PAGE1}}(t-1)\right)\left(1 - e^{-1/\text{FRT}}\right) \\ &+ B(t-1)\end{aligned}$$

where

$$A(t-1) = \frac{\text{ECS}}{F_{sl}\ln 2}F(t-1)$$
$$B(t-1) = \frac{\text{ECS}}{F_{sl}\ln 2}(F(t-1) - F(t-2))$$

$F(t)$ is the anthropogenic forcing in our model

F_{sl} is the forcing slope, 5.5 W/m2

FRT is the warming half-life, from a triangular distribution from 10 to 55 with mode of 20

The surface albedo feedback is then calculated using a quadratic approximation, where SAF decreases more rapidly as temperature increases. The equations are described as an integral over this quadratic:

$$\text{SAF}(t) = \frac{C(\Delta\overline{T_{ATM-PAGE1}}(t)) - \text{FSAF}_0}{\Delta\overline{T_{ATM-PAGE1}}(t) - \Delta\overline{T_{ATM-PAGE1}}(2010)}$$

where

$C(\Delta T) = \beta_2 \Delta T^3/3 + \beta_1 \Delta T^2/2 + \beta_0 \Delta T + \gamma \Delta T \delta$

β_2 is the T^2 coefficient for the SAF quadratic (W/m^2/K^3)

β_1 is the T^1 coefficient for the SAF quadratic (W/m^2/K^2)

β_0 is the T^0 coefficient for the SAF quadratic (W/m^2/K)

γ is the standard deviation of the SAF quadratic (W/m^2/K)

δ is the nonlinearity of SAF, drawn from a symmetric triangular distribution from -1 to 1

FSAF_0 is the base year SAF forcing (W/m^2)

The adjustment to the SAF forcing is given by a two-segment correction

$$\Delta\text{FSAF}(t) = -\,\text{SAF}(t)\Delta\overline{T_{ATM-PAGE2}}(t-1) + \begin{cases} C(\Delta\overline{T_{ATM-PAGE2}}(t-1)) & \text{if } \Delta\overline{T_{ATM-PAGE2}}(t-1) < 10 \\ D(\Delta\overline{T_{ATM-PAGE2}}(t-1)) & \text{if } \Delta\overline{T_{ATM-PAGE2}}(t-1) \geq 10 \end{cases}$$

where

$$D(\Delta T) = \psi + \alpha(\Delta T - 10) + \sigma(\Delta T - 10)\delta$$

$\Delta\overline{T_{ATM-PAGE2}}(t)$ is defined below.

ψ is the integration constant for SAF forcing at the segment switch point

α is the linear SAF segment mean

σ is the linear SAF segment standard deviation

Also using $SAF(t)$, the adjusted ECS and FRT values are calculated as

$$\text{ECS}' = \text{ECS}\left(1 - \frac{\text{ECS}\left(\text{SAF}(t) - \overline{SAF}\right)}{F_{sl}\ln 2}\right)^{-1}$$

$$\text{FRT}' = \text{FRT}\left(1 - \frac{\text{ECS}\left(\text{SAF}(t) - \overline{SAF}\right)}{F_{sl}\ln 2}\right)^{-1}$$

where $\overline{SAF}$ is the constant approximation to the SAF (0.34959 W/m^2/C).

Then $\Delta\overline{T_{ATM-PAGE2}}(t)$, the adjusted temperature time-series, is calculated identically to $\Delta\overline{T_{ATM-PAGE1}}(t)$, but using ECS′, FRT′, and with the additional forcing ΔFSAF(t). The temperature adjustment produced by the SAF model, $\Delta\overline{T_{ATM-PAGE2}}(t) - \Delta\overline{T_{ATM-PAGE1}}(t)$, is then added to the main temperature in the model.

2.1.7 Slowdown of the Atlantic Meridional Overturning Circulation

Weakening of the Atlantic Meridional Overturning Circulation (AMOC) or thermohaline circulation,[9] whether partial or full, has inspired a number of numerical modelling studies in climate economics (6; 66; 11; 59; 29; 32; 36; 67; 48). The majority of these take a stylised approach. Of those aiming for realism, we choose to incorporate the results of Anthoff et al. (6) in our model, because of their unique focus on the effects of AMOC slowdown at the national level. This is arguably central to the economic evaluation of AMOC slowdown, because its physical effects would vary significantly across the world, from a reduction in regional temperature of several degrees, all else being equal, to an increase in regional temperature of a few tenths of a degree (see 6, fig. 1). The basic logic is that the ocean circulation redistributes heat, rather than creating or destroying it, and countries vary in their exposure to this heat redistribution, as well as the effects of global warming more broadly, depending on their physical location. AMOC slowdown is expected to have physical effects other than temperature change, for instance effects on precipitation and regional sea levels (68), but these have yet to be incorporated in economic studies.

[9] We use these two terms interchangeably.

(6) implement four what-if scenarios known in the context of AMOC slowdown as 'hosing experiments'. In these experiments, a large exogenous pulse of freshwater is added to the representation of the North Atlantic in General Circulation Models – hence the term hosing – and the consequences for the AMOC are simulated. The four scenarios result in an AMOC slowdown of 7%, 24%, 27% and 67% respectively. This slowdown is assumed to be reached in the year 2085, after being phased in linearly from a 2050 starting point. As is by now familiar, we convert these what-if scenarios into hazard events and assign them probabilities. The national temperature delta arising from AMOC slowdown is hence given by

$$\begin{aligned} \Delta T_{\mathrm{AT\_AMOC}}(i,t) &= \Delta T_{\mathrm{AT\_AMOC}}(i,t-1) + \left(\frac{\overline{\Delta T_{\mathrm{AT\_AMOC}}(i)}}{\Delta_{\mathrm{AMOC}}}\right) I_{\mathrm{AMOC}}(t) \\ &\iff \sum_{s=0}^{t-1} \Delta T_{\mathrm{AT\_AMOC}}(i,s) < \overline{\Delta T_{\mathrm{AT\_AMOC}}(i)}, \qquad (20) \\ \Delta T_{\mathrm{AT\_AMOC}}(i,t) &= \overline{\Delta T_{\mathrm{AT\_AMOC}}(i)} \\ &\iff \sum_{s=0}^{t-1} \Delta T_{\mathrm{AT\_AMOC}}(i,s) = \overline{\Delta T_{\mathrm{AT\_AMOC}}(i)}, \qquad (21) \end{aligned}$$

where $\overline{\Delta T_{\mathrm{AT\_AMOC}}(i)}$ is the permanent difference in national annual average temperature as a result of AMOC slowdown in country i. The data points corresponding to $\overline{\Delta T_{\mathrm{AT\_AMOC}}(i)}$ were kindly provided by Anthoff and colleagues for all countries they covered. Δ_{AMOC} is the time taken for AMOC slowdown to phase in, i.e. 35 years. $I_{\mathrm{AMOC}}(t)$ is the indicator function, whose transition probability from zero to one is

$$p_{\mathrm{AMOC}}(t) = 1 - \exp\left[-b_{\mathrm{AMOC}} \Delta \overline{T_{\mathrm{AT}}}(t)\right], \qquad (22)$$

conditional on $I_{\mathrm{AMOC}}(t-1) = 0$.

To calibrate the hazard rate for each of the four scenarios in (6), we compile likelihoods as a function of global mean temperature increase for distinct AMOC shutdown events ranging from a weakening of 11% to a full shutdown. We obtain these from the IPCC *Fifth Assessment Report* (69), its *Special Report on Global Warming of 1.5°C* (70), and (71). Given the

limited measurements of AMOC intensity, these numbers reflect a combination of model-based estimates and expert judgement. We proceed in two steps: (i) we take the convex combination of the AMOC shutdown events from the literature that most closely resembles the what-if scenario at hand. To obtain a hazard rate b_{AMOC}, we then (ii) calibrate Equation (22) by minimizing the sum of squared differences to the likelihoods obtained in step (i). We estimate $b_{\text{AMOC}} = 1.6$ for a 7% slowdown, 0.611 for a 24% slowdown, 0.54 for 27% and 0.135 for 67%.

2.1.8 Weakening of the Indian Summer Monsoon

The first integrated assessment of the Indian Summer Monsoon (ISM) and its response to climate change has recently been carried out by Belaia (10). This is based on coupling a version of Nordhaus' regionally disaggregated RICE IAM (72) to a model of the ISM (73). The ISM is driven by greater heating of the land surface relative to the ocean in summer, which creates a pressure gradient that drives moist ocean air over the Indian subcontinent, where it rises and condenses. However, ISM rainfall displays important year-to-year variation and the ISM has the potential to abruptly change regime from wet to dry and *vice versa*. Schewe and Levermann's model generates these dynamics by incorporating reduced-form representations of two competing feedback processes. The first is the so-called moisture advection feedback, a positive feedback whereby monsoon rains release latent heat, which strengthens the monsoon circulation and brings more rainfall in turn. The second is the dry-subsidence effect, a negative feedback whereby high pressure reduces rainfall, the decreased rainfall leads to less latent heat being released, which in turn sustains the dry phase. High pressure also deflects winds away from the monsoon region. In Belaia's model (10), rainfall depends on both climate change, through multiple channels, and regional emissions of sulphur dioxide, which reflect incoming solar radiation, reduce heating over the Indian subcontinent and weaken the ISM.

The key output of the ISM model that feeds into damages to India (see below) is average

rainfall over the Indian subcontinent over the summer monsoon season:

$$\overline{P}(t) = \frac{1}{136} \sum_{d=1}^{136} P(d,t), \tag{23}$$

where $P(d,t)$ is rainfall on day d of year t and there are 136 days in each monsoon season.[10] Each day is either wet or dry, depending on

$$P(d,t) \quad = \quad \begin{cases} P_{\text{wet}}(t), & Pr(d,t) < p(d,t), \\ P_{\text{dry}} & Pr(d,t) \geq p(d,t), \end{cases} \tag{24}$$

where $Pr(d,t) = U(0,1)$, capturing random variation in day-to-day weather. There is no rainfall on a dry day, whereas rainfall on a wet day is an increasing function of atmospheric temperature, since a warmer atmosphere can hold more water:

$$P_{\text{wet}}(t) = p'' \left[\Delta\overline{T_{\text{AT}}}(t) - \Delta\overline{T_{\text{AT}}}(0)\right] + P_{\text{wet}}(0). \tag{25}$$

The initial value of P_{wet} is 9mm per day and it increases by 0.42mm/day/°C of global warming.

The probability of a wet day during the first δ days of the season – the onset – is

$$p_{\text{init}}(t) \quad = \quad \begin{cases} p_{\text{init},1}(t), & A_{\text{pl}}(t) < A_{\text{pl,crit}}(t), \\ 1 - p_{\text{m}}, & A_{\text{pl}}(t) \geq A_{\text{pl,crit}}(t), \end{cases} \tag{26}$$

where $p_{\text{m}} = 0.82$ is the maximum probability of a wet day.[11] The formulation in Eq. (26) makes rainfall during the onset of the season a function of albedo $A_{\text{pl}}(t)$, in particular its relation to a critical albedo value $A_{\text{pl,crit}}(t)$. If the actual albedo exceeds the critical value,

[10] For computational reasons, we use a four-day time step, so $P(d,t)$ changes at most once every four days and there are 136 days in the season, compared with 135 in (10).

[11] By bounding the probability of a wet day during the onset of the monsoon season, the system does not become irrevocably locked into either a wet or dry state.

the probability of a wet day is at its minimum. The critical albedo value is increasing in the atmospheric concentration of CO_2,

$$A_{\mathrm{pl,crit}}(t) = \alpha_{\mathrm{pl},1} \ln \left[\sum_{i=0}^{3} S_i(t) + \underline{S} \right] + \alpha_{\mathrm{pl},2}. \tag{27}$$

$\sum_{i=0}^{3} S_i(t) + \underline{S}$ gives the atmospheric CO_2 concentration and its derivation is explained in the following section. The actual albedo is given by

$$A_{\mathrm{pl}}(t) = A_{\mathrm{pl}}(0) + 2T_{\mathrm{pl}}^2 (1 - A_{\mathrm{s}})^2 \beta_{\mathrm{pl}} \alpha_{\mathrm{pl},3} B_{\mathrm{SO4}}(t), \tag{28}$$

where T_{pl} is the fraction of light transmitted by the aerosol layer, A_{s} is the present value of the surface albedo, β_{pl} and $\alpha_{\mathrm{pl},3}$ are coefficients representing the backscatter fraction and mass scattering efficiency respectively and $B_{\mathrm{SO4}}(t)$ is the regional sulphate burden over the Indian peninsula. This last quantity depends on SO_2 emissions in the region:

$$B_{\mathrm{SO4}}(t) = \mathrm{SO}_2(t) H_{\mathrm{SO2}} V / \Omega. \tag{29}$$

Emissions of SO_2 are exogenous and sourced from the Representative Concentration Pathway (RCP) database (74). The emissions scenarios we use are discussed in greater detail below. The RCP database only disaggregates SO_2 emissions to the level of the Asian continent/region, so we downscale to the Indian level by assuming a constant ratio of Indian/Asian emissions, estimated based on 2010 data (75). The parameter H_{SO2} is the fractional sulphate yield, V is the atmospheric lifetime of sulphate and Ω is the land area. Thus the dependence of rainfall on albedo in the model ultimately captures the local cooling effect of SO_2 emissions in the region, which weakens the ISM.

Assuming the actual planetary albedo does not exceed the critical value, the probability

of a wet day during the first δ days of the season is

$$p_{\text{init},1}(t) = p' \left[m_{\text{NINO3.4}}(t) - m_0\right] + p_0, \tag{30}$$

where $m_{\text{NINO3.4}}$ is the strength of the Walker circulation, i.e. the Pacific Ocean atmospheric circulation, in May. The subscript NINO indicates that the strength of this circulation depends on whether there is an El Niño or not. El Niño suppresses the ISM. The parameters p', m_0 and p_0 are used to calibrate the response of $p_{\text{init},1}(t)$ to $m_{\text{NINO3.4}}$. The strength of the Walker circulation in May is in turn given by

$$m_{\text{NINO3.4}}(t) = m' \left[\Delta\overline{T_{\text{AT}}}(t) - \Delta\overline{T_{\text{AT}}}(0)\right] + m_{\text{NINO3.4}}(0). \tag{31}$$

The probability of a wet day after the first δ days of the season is

$$p(d,t) = \frac{1/\delta \sum_{i=d-\delta}^{d-1} P(i,t) - P_{\text{dry}}}{P_{\text{wet}}(t) - P_{\text{dry}}}, \tag{32}$$

where $\delta = 16$ days.[12] The probability of a wet day depends positively on how wet the previous δ days were, a representation of the moisture advection and dry-subsidence feedbacks.

2.1.9 Tipping point interactions

Tipping points can interact with each other in multiple ways (60; 64). Some of these interactions are hardwired into the structure of our model. For example, the PCF increases GMST, which affects all seven remaining tipping points in our study, because all of them depend on temperature. However, the structure of our model can only capture a limited subset of all the possible interactions between tipping points. To increase the number of interactions, we use the expert elicitation study of Kriegler et al. (64), which attempted to quantify how the triggering of one tipping point can cause the hazard rates of other tipping points to change,

[12]With a four-day time step, we set the memory period $\delta = 16$ days, rather than 17 days as in (10).

with a focus on mechanisms other than temperature.

We apply a hierarchical Bayesian analysis to obtain best estimates of the hazard rate changes provided by the experts in (64). The hazard rate changes – the interactions – are represented by a range for expert i from lower bound u_i to upper bound n_i. Each change/interaction is a multiplier on the base hazard rate, so a value of 1 means no change. We posit a true, expert-specific hazard rate change, θ_i, and further assume that these true values are drawn from a normal distribution with unknown mean and variance. This allows the expert opinions to be partially pooled to inform the hyperparameters of the normal distribution:

$$\theta_i \sim \mathcal{N}(\mu, \tau)$$
$$\theta_i \sim \mathcal{U}(u_i, n_i)$$

We treat cases where experts were uncertain about the lower bound of the hazard rate change as having a lower bound of 0, and cases where they were uncertain about the upper bound as an upper bound of 10. Figure 8 presents the results.

Figure 8: The posterior distribution of μ, the mean of the hyperdistribution, for each interaction. The error bars in each plot show the 95% credible interval on μ for the given interaction. The light grey lines show each expert's upper and lower bounds (dots are used if the upper bound equals the lower bound). Abbreviations are as follows: Atlantic Meridional Overturning Circulation (AMOC), melt of the Greenland Ice Sheet (GIS), disintegration of the West Antarctic Ice Sheet (WAIS), and dieback of the Amazon rainforest (AMAZ).

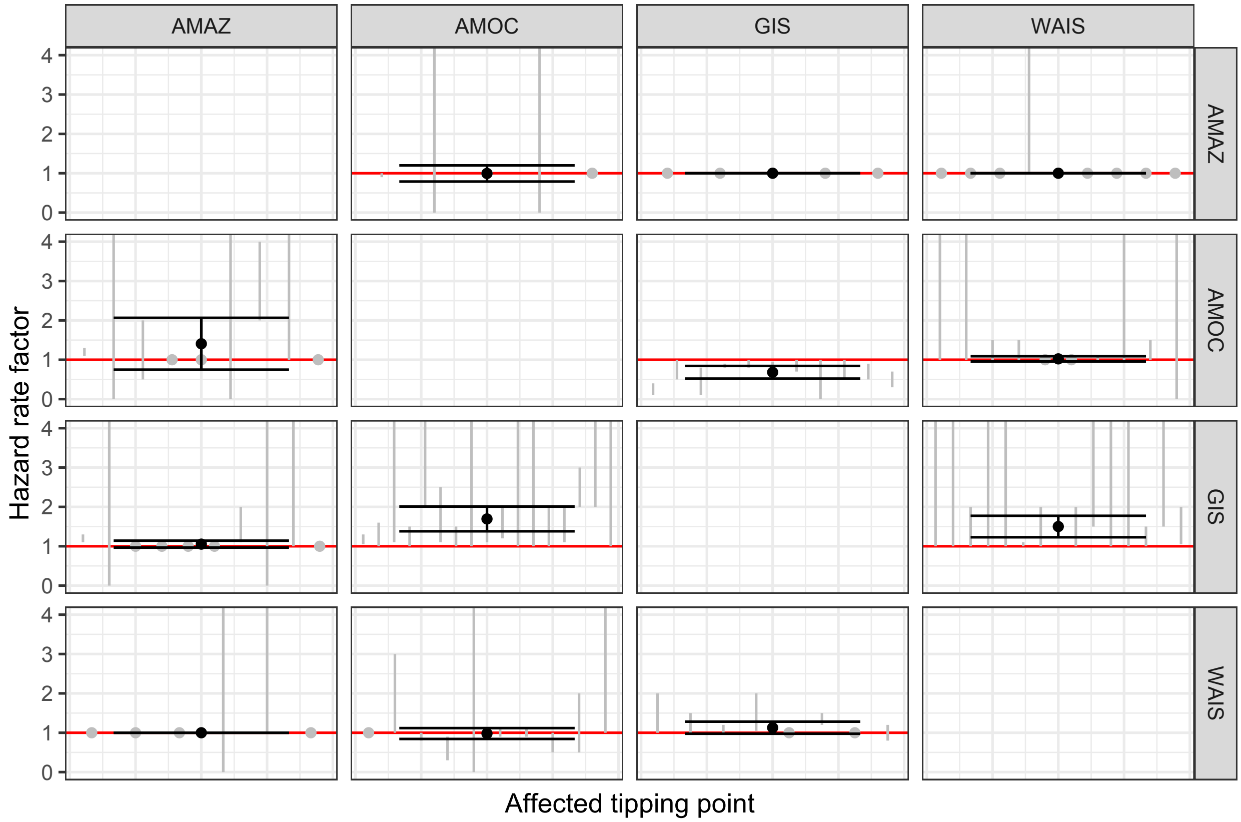

The set of tipping point interactions included in our study is the union of the set of interactions hardwired in our model and the set of interactions quantified by (64). To aid understanding of how many interactions are thereby included, as well as the direction of each interaction, Table 4 provides a matrix.

Table 4: Interactions between tipping points included in this study. Each cell indicates the qualitative effect of the row tipping point on the column tipping point. Where the row tipping point can increase or decrease the intensity/likelihood of the column tipping point, depending on time or state, we write +/-. Parentheses indicate the interaction is calibrated on the expert elicitation study by (64). The absence of parenthesis indicates the interaction is hardwired in the model structure. Zeros indicate an interaction that is included, but that has a statistical zero effect according to (64). No int. means the interaction is not included at all. n.b. ISM affects other tipping points via ENSO, implicit in the expert estimates of the relevant hazard rate changes.

	PCF	OMH	SAF	AMAZ	GIS	WAIS	AMOC	ISM
PCF		+	+	+	+	+	+	+/-
OMH	+		+	+	+	+	+	+/-
SAF	+/-	+/-		+/-	+/-	+/-	+/-	+/-
AMAZ	+	+	+		+ (0)	+ (0)	+ (+/-)	+/- (+/-)
GIS	no int.	no int.	no int.	(+/-)		(+)	(+)	(0)
WAIS	no int.	no int.	no int.	(0)	(+/-)		(+/-)	(0)
AMOC	no int.	no int.	no int.	(+/-)	(-)	(+/-)		(0)
ISM	no int.	no int.	no int.	(+)	(0)	(0)	(+/-)	

2.2 Climate module

2.2.1 Emissions

The principal inputs to the climate model are global emissions of CO_2 and CH_4. There are two sources of these. The first is anthropogenic emissions from burning fossil fuels, from industrial processes, land use and land-use change, and waste disposal. Anthropogenic emissions are exogenous and sourced from the RCP database (74),[13] giving four emissions scenarios estimated in order to match prescribed paths for radiative forcing.[14] Estimating the SCC requires a time horizon of several hundred years be considered, due to the long atmospheric lifetime of CO_2. Therefore we use the RCPs extended to 2300 by (76). Other anthropogenic

[13]http://www.iiasa.ac.at/web-apps/tnt/RcpDb

[14]RCP2.6 peaks at ~3 Wm^{-2} before 2100 and then declines; RCP4.5 reaches ~4.5 Wm^{-2} at stabilisation after 2100; RCP6 reaches ~6 Wm^{-2} at stabilisation after 2100; RCP8.5/baseline exceeds 8.5 Wm^{-2} in 2100.

and natural sources of radiative forcing, both positive and negative, are aggregated into an exogenous residual radiative forcing series.[15] Projections of these sources are also taken from the extended RCP database. The second source of emissions is the carbon-cycle feedbacks described in the previous section, i.e. permafrost melting, dissociation of ocean methane hydrates, and Amazon rainforest dieback.

2.2.2 CO_2 and CH_4 cycles

CO_2 emissions are fed into the FAIR model of the carbon cycle (77). FAIR builds on the model of (78), which was designed to emulate a diverse set of carbon-cycle models of different complexity for an inter-comparison project. FAIR adds to this a reduced-form representation of positive carbon-cycle feedbacks, whereby the rate of CO_2 uptake by ocean and terrestrial carbon sinks is decreasing in cumulative CO_2 uptake by those sinks, and in temperature. The most important of these feedbacks is saturation of the ocean carbon sink.

In the model, the atmospheric stock of carbon is partitioned into four boxes, each of which decays at a different rate:

$$S_i(t) = a_i \sum CO_2(t-1) + \frac{(1-\delta_i)}{\alpha(t)} S_i(t-1),\ i \in \{0,1,2,3\}, \tag{33}$$

where $S_i(t)$ is the stock of carbon in box i and $\sum CO_2(t) = CO_{2\_EX}(t) + CO_{2\_PF}(t) + CO_{2\_AMAZ}(t)$. $CO_{2\_EX}(t)$ stands for exogenous, anthropogenic emissions from the RCPs. The coefficients a_i determine the fraction of emissions entering each box; $\sum_{i=0}^{3} a_i = 1$. To emulate the behaviour of the representative carbon-cycle model in (78), i.e. the multi-model average, it so happens that the allocation between the four boxes is of the order of 25% each (more precisely, 22-28%). The coefficients δ_i are the decay rates, which range from approximately zero, loosely corresponding to the time taken for CO_2 to be sequestered by

[15] This is the sum of forcing from: (i) N_2O; (ii) flourinated gases controlled under the Kyoto Protocol; (iii) ozone-depleting substances controlled under the Montreal Protocol; (iv) total direct aerosol forcing; (v) the cloud albedo effect; (vi) stratospheric and tropospheric ozone forcing; (vii) stratospheric water vapour from methane oxidisation; (viii) land-use albedo; (ix) black carbon on snow.

geological sedimentation, to around 23%, corresponding to rapid removal of atmospheric CO_2 by the biosphere and upper oceans (77).

The coefficients $\alpha(t)$ represent the positive carbon-cycle feedbacks, slowing down the rate of removal of atmospheric CO_2 from each box. In turn, $\alpha(t)$ is a function of the integrated CO_2 impulse response function in FAIR over 100 years ($iIRF_{100}$). In other words, $iIRF_{100}$ is the average airborne fraction of the CO_2 impulse over a period of time (in this case 100 years), multiplied by that period of time. In FAIR, $iIRF_{100}$ is modelled in reduced form as a linear function of temperature and cumulative emissions absorbed by carbon sinks:

$$iIRF_{100}(t) = r_{\text{pre}} + r_T \Delta \overline{T_{\text{AT}}}(t) + r_C \left[\sum_{s=\text{pre}}^{t} \sum \text{CO}_2(t) - \sum_{i=0}^{3} (S_i(s) - \underline{S}) \right], \tag{34}$$

where $r_{\text{pre}} = 34.4$ years is the estimated pre-industrial value of $iIRF_{100}$,[16] $r_T = 4.165$ years/°C, $r_C = 0.019$ years/GtC, and $\underline{S}$ is the pre-industrial concentration of atmospheric CO_2, 278ppm. $iIRF_{100}$ can take a maximum value of 96.6, otherwise the model becomes unstable (77).[17]

The relationship between $\alpha(t)$ and $iIRF_{100}(t)$ has no analytical solution. We estimate $\alpha(t)$ by fitting an exponential function,[18] which gives

$$\alpha(t) = \chi_1 \exp[\chi_2 iIRF_{100}(t)] \tag{35}$$

where $\chi_1 = 0.0107$ and $\chi_2 = 0.0866$.

CH_4 has a much shorter atmospheric residence time than CO_2 and its decay can be adequately represented by a simple one-box model:

$$M(t) = \underline{M} + (1 - \varrho)(M(t-1) - \underline{M}) + \sum \text{CH}_4(t-1), \tag{36}$$

[16] We use a constant of 34.4 instead of 32.4 as per the original FAIR model (77) in order to obtain a better fit under current and future conditions. A value of 32.4 better fits decay under pre-industrial conditions (77, table 2).

[17] If $iIRF_{100} > 100$, the atmospheric concentration of CO_2 grows without bound in response to an emissions impulse.

[18] With thanks to Frank Venmans.

where $M(t)$ is the atmospheric CH_4 concentration, $\underline{M}$ is the pre-industrial atmospheric CH_4 concentration and $\sum CH_4(t) = CH_{4\_EX}(t) + CH_{4\_PF}(t) + CH_{4\_OMH}(t)$. We assume CH_4 has an atmospheric lifetime of 12.4 years (79), implying a decay rate $\varrho = 8.1\%$.

2.2.3 Radiative forcing and temperature

We specify Arrhenius' logarithmic relationship between radiative forcing and atmospheric CO_2:

$$F_{CO_2}(t) = F_{2\times CO_2}\left(\log_2 \frac{\sum_{i=0}^{3} S_i(t) + \underline{S}}{\underline{S}}\right), \tag{37}$$

where $F_{2\times CO_2}$ is the radiative forcing resulting from a doubling of atmospheric CO_2.

For radiative forcing from atmospheric CH_4, we use IPCC's simplified expression (79):

$$F_{CH_4}(t) = \alpha\left[\sqrt{M(t)} - \sqrt{\underline{M}}\right] - \left[f\left[M(t), N(0)\right] - f\left[\underline{M}, N(0)\right]\right], \tag{38}$$

There is significant overlap between some of the infrared absorption bands of CH_4 and nitrous oxide (80), which means that radiative forcing from atmospheric CH_4 is not independent of the atmospheric concentration of nitrous oxide. That is why atmospheric nitrous oxide appears in (38), both via the initial concentration $N(0)$ and

$$f\left[M(t), N(t)\right] = 0.47\ln\left[1 + 2.01\text{E}^{-5}\left[M(t)N(t)\right]^{0.75} + 5.31\text{E}^{-5}M(t)\left[M(t)N(t)\right]^{1.52}\right]. \tag{39}$$

Overall radiative forcing is the sum of the contributions from atmospheric CO_2 and CH_4, as well as the residual forcing from other greenhouse gases and drivers:

$$F(t) = F_{CO_2}(t) + F_{CH_4}(t) + F_{EX}(t). \tag{40}$$

From forcing, the increase in GMST is governed by a model comprising two heat boxes, one for the atmosphere, land surface and upper oceans $\Delta\overline{T_{AT}}$ and one for the lower/deep oceans $\Delta\overline{T_{LO}}$. This is the same model structure used by Nordhaus in DICE and it has

separately been shown to emulate well the temperature response to emissions of a wide range of General Circulation Models (81), albeit with a different parameterisation than Nordhaus. The equations of motion for $\Delta\overline{T_{\text{AT}}}$ and $\Delta\overline{T_{\text{LO}}}$ are

$$\begin{aligned}\Delta\overline{T_{\text{AT}}}(t) &= \Delta\overline{T_{\text{AT}}}(t-1) + \\ &\quad \frac{1}{C_{UP}}\left[F(t) - \frac{F_{2\times\text{CO}_2}}{\varsigma}\Delta\overline{T_{\text{AT}}}(t-1) - \gamma\left[\Delta\overline{T_{\text{AT}}}(t-1) - \Delta\overline{T_{\text{LO}}}(t-1)\right]\right], \quad (41)\\ \Delta\overline{T_{\text{LO}}}(t) &= \Delta\overline{T_{\text{LO}}}(t-1) + \frac{\gamma}{C_{LO}}\left[\Delta\overline{T_{\text{AT}}}(t-1) - \Delta\overline{T_{\text{LO}}}(t-1)\right], \quad (42)\end{aligned}$$

where C_{UP} and C_{LO} are the effective heat capacities of the upper and lower oceans per unit area, respectively, ς is the equilibrium climate sensitivity or ECS and γ is a coefficient of heat exchange between the upper and lower oceans.

Figure 11 in the Supplementary Results section compares the RCP temperature projections of our climate module with the corresponding projections of the CMIP5 ensemble and shows that they are in close agreement.

2.3 Damages/economic module

2.3.1 Sea level rise

Sea level rise comprises a contribution from thermal expansion and melt from glaciers and small ice caps, $SLR_{\text{THERM}}(t)$, as well as a contribution from disintegration of the GIS and WAIS:

$$\sum SLR(t) = SLR_{\text{THERM}}(t) + SLR_{\text{GIS}}(t) + SLR_{\text{WAIS}}(t). \quad (43)$$

Sea level rise is defined relative to the year 2000 and $\sum SLR(0) = 0.04\text{m}$ (82). To model the contribution from thermal expansion and melt from glaciers and small ice caps, we follow (61) in specifying SLR as a linear function of warming:

$$SLR_{\text{THERM}}(t) = (r_{\text{TE}} + r_{\text{GSIC}})\,\Delta\overline{T_{\text{AT}}}(t) + SLR_{\text{THERM}}(t-1), \quad (44)$$

where $r_{\mathrm{TE}} = 0.00078$ and $r_{\mathrm{GSIC}} = 0.00081$ parameterise the rates of SLR from thermal expansion and melt from glaciers and small ice caps respectively. Sea level rise from thermal expansion is parameterised such that 1°C warming results in a very long-term equilibrium increase of 0.5m (i.e. over the course of approximately 1000 years).

2.3.2 National temperature

We want to implement climate damages at the national level, both to make best use of available empirical damage estimates, which are now at the national level, and to accurately model the impacts of AMOC slowdown in particular, which vary from country to country. We use the damage estimates of (83). In order to use these, we need to convert the increase in GMST relative to pre-industrial into the *level* of national mean surface temperature. We do this by means of statistical downscaling, before subsequently adding the effect on the level of national mean surface temperature of AMOC slowdown. For country i, statistical downscaling involves estimating the ratio of national mean surface temperature to global mean surface temperature $\lambda(i,t)$ using the following equation:

$$\lambda(i,t) = \alpha(i) + \beta(i) \ln \left[\overline{T_{\mathrm{AT}}}(0) - \overline{T_{\mathrm{AT}}}(\mathrm{pre}) + \Delta\overline{T_{\mathrm{AT}}}(t) - \Delta\overline{T_{\mathrm{AT}}}(0)\right], \tag{45}$$

where $\overline{T_{\mathrm{AT}}}(0)$ is the level of global mean surface temperature at $t = 0$, i.e. 2010, and $\overline{T_{\mathrm{AT}}}(\mathrm{pre})$ is an estimate of the pre-industrial GMST. The estimating equation therefore follows the logic that each country's mean temperature converges to a long-term equilibrium difference with respect to the global mean. The country-level coefficients $\alpha(i)$ and $\beta(i)$ are estimated by OLS according to the expression above, pooling data for RCP 4.5 and 8.5.

National mean surface temperature is estimated by applying the coefficients $\lambda(i,t)$ to the level of GMST at time t and adding the change in national mean surface temperature due to AMOC slowdown:

$$T_{\mathrm{AT}}(i,t) = \lambda(i,t)\left[\overline{T_{\mathrm{AT}}}(0) + \Delta\overline{T_{\mathrm{AT}}}(t) - \Delta\overline{T_{\mathrm{AT}}}(0)\right] + \Delta T_{\mathrm{AT\_AMOC}}(i,t) \tag{46}$$

2.3.3 Damages and national income per capita

Income growth depends on exogenous drivers, as well as damages from changing temperatures and from sea level rise (and from the summer monsoon in India, only). Post-damage income per capita in country i, $y(i,t)$, grows according to

$$y(i,t) = \overline{y}(i,t-1)\left[1 + g_{\mathrm{EX}}(i,t) + D_{\mathrm{TEMP}}(i,t)\right]\left[1 - D_{\mathrm{SLR}}(i,t)\right], \tag{47}$$

where $g_{\mathrm{EX}}(i,t)$ is an exogenous, country- and time-specific growth rate that is taken from the Shared Socio-Economic Pathway (SSP) database (84).[19] The SSPs were designed as a flexible accompaniment to the RCP emissions scenarios.[20] The SSP scenarios are only defined until 2100. To extend these scenarios until 2300, we follow a procedure described in Section 2.4.1. $D_{\mathrm{TEMP}}(i,t)$ are temperature damages and $D_{\mathrm{SLR}}(i,t)$ are SLR damages.

The level of income per capita in the previous year, on which damages in the current year work,

$$\overline{y}(i,t-1) = \varphi y_{\mathrm{EX}}(i,t-1) + (1-\varphi)\, y(i,t-1), \tag{48}$$

where $y_{\mathrm{EX}}(i,t-1)$ is counterfactual income per capita, also taken from the SSP database, $y(i,t-1)$ is the *actual* post-damage income per capita experienced, and φ parameterises the weight given to each. This specification enables us to explore two different interpretations of the empirical evidence on temperature damages. The first interpretation is that temperatures impact the level of income in each year, in effect driving a wedge between what output is feasible given implicit factors of production and productivity, and what output is actually achieved. This has been the traditional approach in climate economics, e.g. in Nordhaus' DICE model. The production possibilities frontier is assumed to evolve exogenously. Such 'levels' damages correspond with $\varphi = 1$. The second interpretation is that temperatures

[19] https://tntcat.iiasa.ac.at/SspDb

[20] Different SSPs can be matched with different RCPs, though not all combinations are plausible, and some combinations might be regarded as most likely. We match SSP1 (sometimes called the 'Sustainability' scenario) with RCP3-PD/2.6. SSP2 ('Middle of the Road') is matched with RCP4.5, SSP4 ('Inequality') is matched with RCP6 and SSP5 ('Fossil-fueled development') is matched with both RCP4.5 and RCP8.5.

impact the growth rate of income by directly impacting the accumulation of factors of production and/or by impacting productivity growth (85). Such 'growth' damages correspond with $\varphi = 0$. Our main specification is an intermediate value of $\varphi = 0.5$.

Temperature damages themselves are given by

$$D_{\text{TEMP}}(i,t) = \beta_{1i}\left[T_{\text{AT}}(i,t) - T_{\text{AT}}(i,1990)\right] + \beta_{2i}\left[T_{\text{AT}}(i,t) - T_{\text{AT}}(i,1990)\right]^2, \qquad (49)$$

where the coefficients β_{1i} and β_{2i} are calibrated on the empirical results of (83). The procedure is described in Section 2.4.2.

SLR damages are given by

$$D_{\text{SLR}}(i,t) = \theta(i)\sum SLR(t), \qquad (50)$$

where $\theta(i)$ parameterises the cost to country i per unit SLR. Like (3), we obtain SLR damages from Diaz's CIAM model (5), but we preserve the country resolution. We run CIAM to obtain estimates of national coastal damage/adaptation costs as a function of SLR in two scenarios, (i) no adaptation and (ii) optimal adaptation. We treat each country's adaptation decisions as uncertain and obtain a symmetrical triangular distribution for each $\theta(i)$ with a minimum corresponding to costs in (i) and a maximum corresponding to costs in (ii). We use costs/SLR in 2050 for the calibration, a simple approach facilitated by the fact that the relationship between the two is approximately linear over the 21st century (5).

In India, there is an additional damage multiplier $D_{\text{ISM}}(IND,t)$, so that national income per capita is given by

$$\begin{aligned} y(IND,t) &= \overline{y}(IND,t-1)\left[1 + g_{\text{EX}}(IND,t) + D_{\text{TEMP}}(IND,t)\right] \times \\ &\quad \times \left[1 - D_{\text{SLR}}(IND,t)\right]\left[1 - D_{\text{ISM}}(IND,t)\right]. \end{aligned} \qquad (51)$$

Following (10), the ISM damage multiplier is given by

$$D_{\text{ISM}}(t) \quad = \quad \begin{cases} D_{\text{drought}}, & \overline{P}(t) \leq \overline{P}_{\text{drought}}, \\ 0, & \overline{P}_{\text{drought}} < \overline{P}(t) < \overline{P}_{\text{flood}}, \\ D_{\text{flood}}, & \overline{P}(t) \geq \overline{P}_{\text{flood}}. \end{cases} \tag{52}$$

This structure implies that only extremely wet monsoon seasons and extremely dry monsoon seasons affect income in India, with the measure of precipitation being average rainfall for the monsoon season $\overline{P}(t)$ from Eq. (23). The drought threshold $\overline{P}_{\text{drought}} = 2.8667\text{mm/day}$, while the equivalent flood threshold $\overline{P}_{\text{flood}} = 7.6667\text{mm/day}$. Drought-related damages $D_{\text{drought}} = 3.5\%$ of GDP, while flood-related damages $D_{\text{flood}} = 0.85\%$. All these parameter values are taken from (10).

2.3.4 Utility and welfare

Post-damage national income per capita is first converted into consumption per capita using a country-specific but time-invariant savings rate,

$$c(i,t) = [1 - s(i)]\, y(i,t), \tag{53}$$

where the country savings rates $s(i)$ are calibrated on observed national savings rates averaged over the period 2005-2015, using World Bank data. Savings data are missing for many countries, in which case we impute the global average, also obtained from the World Bank. This specification assumes savings are exogenous and do not respond to changing income prospects. Fully endogenous savings are computationally infeasible in a model with this much complexity and detail. The limitations of assuming constant/exogenous savings have been discussed in the literature, e.g. (86). Small to moderate climate damages do not appear to shift savings rates measurably. Large damages can do so, however. In Section 3, we report a sensitivity analysis, in which we shift all countries' savings rates up and down by a fixed

amount.

Consumption is converted into utility using a standard, constant-elasticity-of-substitution representation,

$$u(i,t) = \frac{c(i,t)^{1-\eta}}{1-\eta}, \tag{54}$$

where η is the elasticity of marginal utility of consumption.

To compute overall welfare, we specify a discounted classical/total utilitarian social welfare functional. We begin by calculating welfare for each country i:

$$W(i) = \sum_{t=2020}^{T} (1+\rho)^{-t} u(i,t) L(i,t), \tag{55}$$

where ρ is the utility discount rate, a.k.a. the pure rate of time preference. Discounted, population-adjusted current period utility is then summed over the whole modelling horizon to obtain total welfare. Population data are exogenous and taken from the SSP scenarios.

Global welfare follows naturally as the sum of welfare across all countries i:

$$W = \sum_i W(i) \tag{56}$$

2.3.5 Computing the social cost of carbon

The social cost of carbon along a particular scenario of emissions, income and population is the difference in welfare caused by a marginal emission of CO_2, normalised by the marginal welfare value of a unit of consumption in the base year:

$$\text{SCC}(t) = \frac{\partial W/\partial E(t)}{\partial W/\partial c(t)}. \tag{57}$$

To calculate the numerator, we run the model twice with identical assumptions, the second time with an additional pulse of emissions. Let θ_m represent a vector of parameter values from the model as described in the preceding sections. These are in most cases random draws

from a distribution, including individual tipping event realisations. Then we calculate

$$\left[\frac{\partial W}{\partial E(t)}\right]_m = \frac{W[E(t) + \Delta_E(t), \theta_m] - W[E(t), \theta_m]}{\Delta_E(t)}, \tag{58}$$

where Δ_E is the emissions pulse. We focus on an emissions pulse in 2020.

The denominator of (57), $\partial W/\partial c(t)$, depends on the consumption level of the normalising agent. We define this as the global average individual, i.e. global mean consumption per capita:

$$\bar{c}(t, \theta_m) = \frac{\sum_i c(i, t, \theta_m) L(i, t)}{\sum_i L(i, t)}. \tag{59}$$

Note that this is also uncertain and depends on the vector of random parameters. Differentiating the utility function, we then have

$$\left[\frac{\partial W}{\partial c(t)}\right]_m = \bar{c}(i, t, \theta_m)^{-\eta}. \tag{60}$$

We focus on a base year of 2020.

We then calculate the negative of the ratio of equations (58) and (60) for each draw of random parameters m and take expectations over all draws.

Below in Section 3.2 we present a range of sensitivity analyses. To implement these, we take the relevant subset of model parameters and vary either their fixed values or distributions, holding all other parameters or parameter distributions constant. We also look at different emissions/socio-economic scenarios. For these sensitivity analyses, all the model parameters and parameter distributions are held constant – only the exogenous emissions/socio-economic variables are varied.

The numeraire in the model is year 2010 US dollars, corresponding to the year in which GDP is initialised. We inflate our reported SCC values to year 2020 US dollars using a factor of 1.2, based on data from (87).[21]

[21]The inflation factor is 1.2 whether one uses the Consumer Price Index or the GDP deflator.

2.3.6 Non-market damages

The above damages from temperature, SLR and the ISM can be regarded as 'market' damages. They do not include estimates of the welfare cost of climate change outside markets, for example damages to ecosystems that can be priced at people's willingness to pay (WTP) to preserve those ecosystems' existence. 'Non-market' damages are more uncertain than their market counterparts, but in many IAMs they occupy a substantial share of total welfare damages from climate change (e.g. 88), so we add an estimate of them as a sensitivity check.

We use the non-market damage module of the MERGE IAM (89). The MERGE model places particular emphasis on the representation of non-market damages, with a willingness-to-pay (WTP) measure that depends on both income and temperature. While the parameters of the MERGE non-market damages are speculative, its use of an S-shaped elasticity of income seems like a sensible starting point. The WTP to avoid warming as a function of income is shown in figure 9.

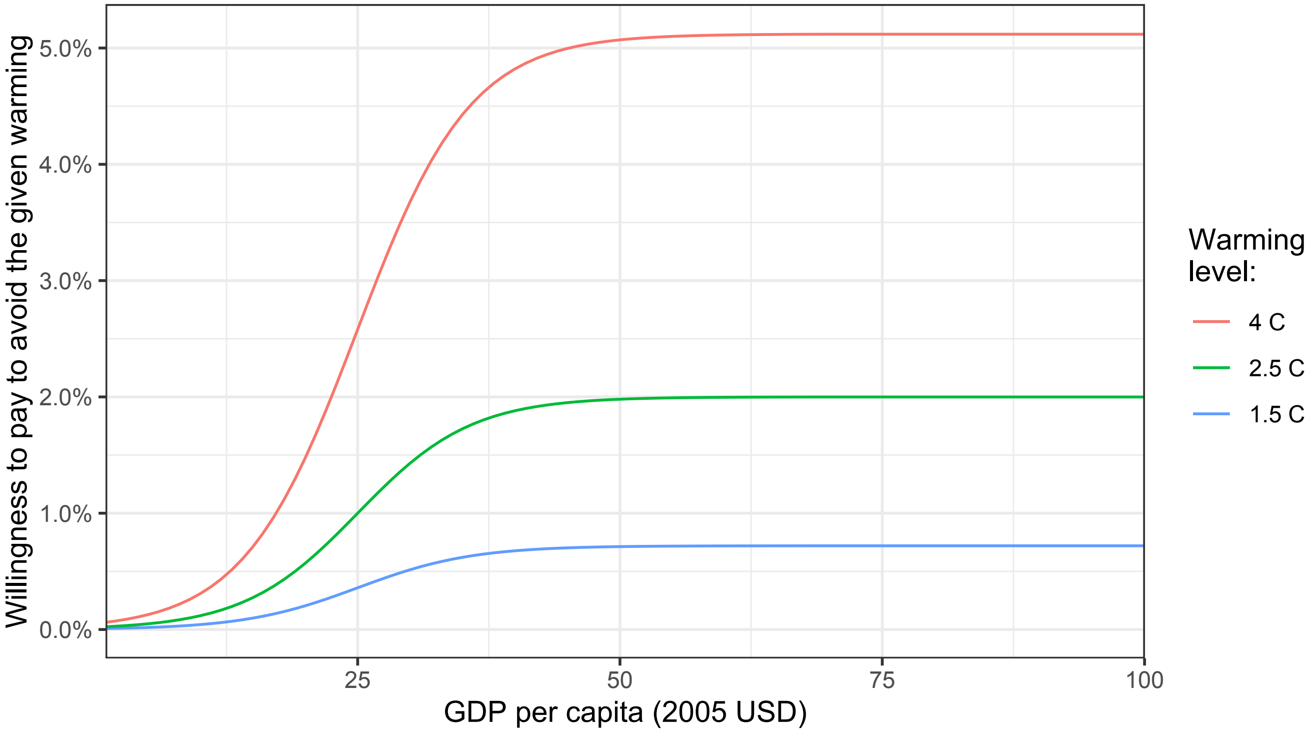

Figure 9: Willingness to pay to avoid 1.5, 2.5, and 4 C, as a function of income, as described by the MERGE model.

We calculate this WTP measure at a national level. The non-market damage multiplier, or economic loss function, is

$$D_{\text{NM}}(i,t) = \left[1 - \left(\overline{T_{\text{AT}}}(t)/\Delta T_{\text{cat}}\right)^2\right]^{h(i,t)}. \tag{61}$$

This is a hockey-stick function embodying the assumption that non-market damages can increase rapidly as temperatures become more extreme. ΔT_{cat} is a catastrophic warming parameter set to 17.68°C, which people are assumed to be willing to avoid at any cost[22]. $h(i,t)$ is the hockey-stick parameter, which depends on country income per capita:

$$h(i,t) = \min\left[\frac{\log\left[1 - D_{\text{ref}}/1+100\exp\left[-WTP_{\text{ref}}\cdot y(i,t)\right]\right]}{\log\left[1 - \left(\Delta T_{\text{ref}}/\Delta T_{\text{cat}}\right)^2\right]}, 1\right], \tag{62}$$

where

$$\begin{aligned} WTP_{\text{ref}} &= 0.184206807 \quad \text{WTP 1\% of GDP to avoid reference warming at \$25k/capita} \\ D_{\text{ref}} &= 0.02 \quad \text{WTP loss at reference warming} \\ \Delta T_{\text{ref}} &= 2.5\,\text{C} \quad \text{WTP reference warming} \end{aligned}$$

As income increases above $25k/capita, the WTP to avoid 2.5 C warming increases from 2%.

The non-market damage multiplier is applied to country-level utility:

$$u(i,t) = u\left(D_{\text{NM}}(i,t)c(i,t)\right)$$

for utility function $u(\cdot)$ as specified above.

[22]The catastrophic warming temperature is derived from the assumption that economic losses rise quadratically, and are calibrated to a loss of 2% at 2.5C warming.

2.4 Supporting analysis

2.4.1 Extending the SSP scenarios beyond 2100

To estimate post-2100 income and population along the SSP scenarios, we fit a model to the available pre-2100 SSP scenario data and use the fitted model to extrapolate. The same model is applied to both income and population and is defined in terms of growth rates. The model postulates that changes in pre-2100 income and population growth rates are explained by a rate of convergence and a rate of decay.

The model is as follows:

$$\text{Growth}(i,t) = (1-\beta-\delta)\text{Growth}(i,t-1) + \delta\text{MeanGrowth}(t-1), \tag{63}$$

where δ is the rate of convergence, β is the decay rate and

$$\text{MeanGrowth}(t-1) = \sum_i \frac{\text{Population}(i,2015)}{\sum_j \text{Population}(j,2015)}\text{Growth}(i,t-1). \tag{64}$$

Below, we write this as $\text{Growth}(\cdot,t-1)\cdot w$, where w is the vector of global population shares for each country.

SSP data are not available in every year, so fitting Eq. (63) requires a model with dynamics. We use a two-step approach, fitting the model using Stan, a computational Bayes system. The first step uses the available data directly, fitting

$$\text{Growth}(i,s) \sim \mathcal{N}\left([1-\Delta t(\beta+\delta)]\text{Growth}(i,s-1) + \Delta t\delta\text{MeanGrowth}(s-1), \sigma_i\right), \tag{65}$$

where s is a time step, Δt is the number of years between time steps, and country i has uncertainty σ_i. We apply a prior that both β and δ are between 0 and 0.5.

Next, we fit the full model, using the results of the simplified model to improve the Bayesian model convergence. In this case, for a given Markov chain Monte Carlo draw of β

and δ, we calculate the entire time series:

$$\widehat{\text{Growth}}(i,t) \sim \mathcal{N}\left((1-\beta-\delta)\widehat{\text{Growth}}(i,t-1) + \delta\left[\widehat{\text{Growth}}(\cdot,t-1)\cdot w.\right], \sigma_i\right) \tag{66}$$

starting with $\widehat{\text{Growth}}(i, 2015)$ as reported in the SSP dataset.

The probability evaluation is over both the performance of the fit and the priors:

$$\text{Growth}(i,s) \sim \mathcal{N}\left(\widehat{\text{Growth}}(i,t(s)), \sigma_i\right)$$
$$\beta \sim \mathcal{N}(\mu_\beta, \sigma_\beta)$$
$$\delta \sim \mathcal{N}(\mu_\delta, \sigma_\delta)$$
$$\log \sigma_i \sim \mathcal{N}(\mu_{\sigma,i}, \sigma_{\sigma,i})$$

where μ is the mean estimate of the corresponding parameter and σ is the standard deviation across its uncertainty. The prior for σ_i is defined as a log-normal, centered on the mean of the estimates of $\log \sigma_i$. The estimates for each SSP are shown in Table 5.

Table 5: Estimated convergence and decay rates for extrapolation of growth of GDP per capita and population in the SSP socio-economic scenarios beyond 2100

SSP	Variable	δ	β
1	GDP per capita	0.006205028	0.005930520
1	Population	0.008967453	0.005215835
2	GDP per capita	0.004190444	0.007228942
2	Population	0.001276993	0.011064426
3	GDP per capita	0.006273030	0.009597363
3	Population	0.001064697	0.007688331
4	GDP per capita	0.006895296	0.009651277
4	Population	0.001867587	0.003461600
5	GDP per capita	0.007766807	0.003843256
5	Population	0.003470952	0.004305310

2.4.2 Calibration of country-specific damage functions

We develop damage functions based on the econometric estimates of Burke et al. (83) (pooled global estimates without lags). Since the independent variable used in the estimation process of (83) is annual average temperature, a few steps are necessary to develop functions indexed to climatic average temperature, as produced by our model. These steps follow the basic procedure described in (90):

1. Using the replication materials provided for (83), estimate their standard model coefficients, along with their associated variance-covariance matrix.

2. Generate annual average temperatures for each country, as projected by a range of bias-corrected, statistically-downscaled GCMs. Temperature averages are population weighted, according to gridded population data from (91). We use the set of downscaled models from (92), supplemented by surrogate pattern-scaled models representing the tails of the GMST probability distribution as described in (90).

3. Estimate growth impacts for each country, year, GCM, and RCP. We take 3000 random Monte Carlo draws from the uncertainty in the coefficients, using a multivariate normal distribution with the variance-covariance matrix estimated above. The growth impacts are reported as changes in the log of country-specific GDP per capita, and we do not accumulate growth impacts over time.

4. Compute the difference between the estimated, un-normalized growth rate in each year and the average of the estimated growth rates from 1981-2000. We call this $\Delta \log y(i, r, t)$, where r stands for the RCP.

5. Calculate the average changes in growth rates across all GCMs and Monte Carlo runs, for each RCP, year and country, and the standard deviation across growth rates. We weight GCMs as done in (90).

6. Calculate the climatic temperature $T_{\text{AT}}(i,r,t)$ as the weighted average across all GCMs of its area-weighted average temperature. The GCMs are weighted as above.

7. For each country, pool the results for all years and RCPs. Use these data to estimate the following regression model:

$$\begin{aligned}\Delta \log y(i,r,t) &= \alpha + \beta_1 \left[T_{\text{AT}}(i,r,t) - T_{\text{AT}}(i,r,1990)\right] \\ &\quad + \beta_2 \left[T_{\text{AT}}(i,r,t) - T_{\text{AT}}(i,r,1990)\right]^2 + \epsilon(i,r,t)\end{aligned}$$

   where $T_{\text{AT}}(i,r,1990)$ is the average climatic temperature from 1981 to 2000. The regression observations are weighed by inverse variance.

Figure 10 compares the marginal effect of temperature on growth rates using this method with the marginal effect obtained by applying the coefficients from (83) directly. On average, these values are lower, due to Jensen's inequality, but the mismatch between population-weighted and area-weighted temperatures can result in marginal effects above the untransformed rate.

Figure 10: Comparison between the marginal effect of temperature on growth rates for each country (red) and using the coefficients from (83) directly (blue).

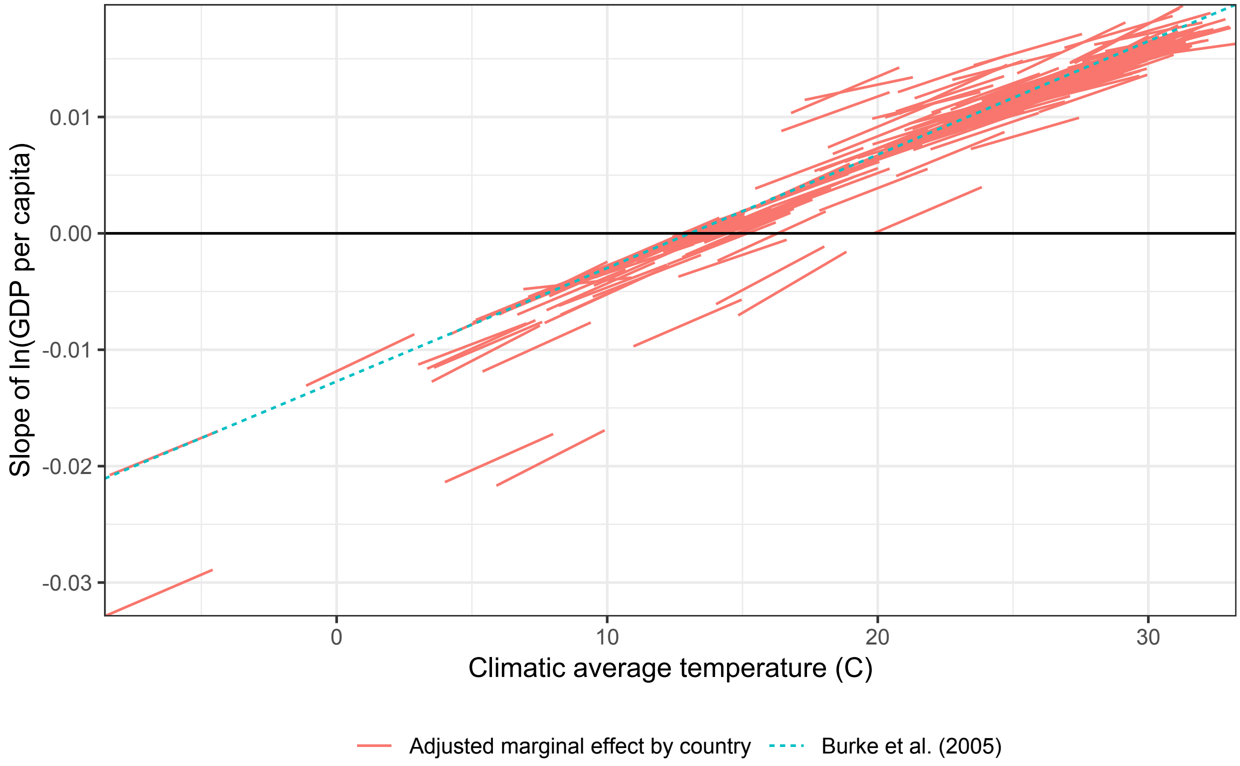

3 Supplementary results

3.1 Climate model comparison

Figure 11 compares the 21st century GMST increase projected by our climate model – tipping point modules turned off – with the corresponding multi-model mean projection from the CMIP5 ensemble (69), for consistent forcing from the RCP scenarios. We set our climate model parameters to their central estimates.

The projections are almost identical for RCP3-PD/2.6. For the remaining three higher-emissions scenarios, our model projects slightly higher temperatures, though in all cases our model projection is well within the 90% confidence interval of CMIP5 models (see 69, figure TS.15).

Figure 11: Comparison between temperature projections of our climate model and the multi-model mean projection from the CMIP5 ensemble as reported in (69).

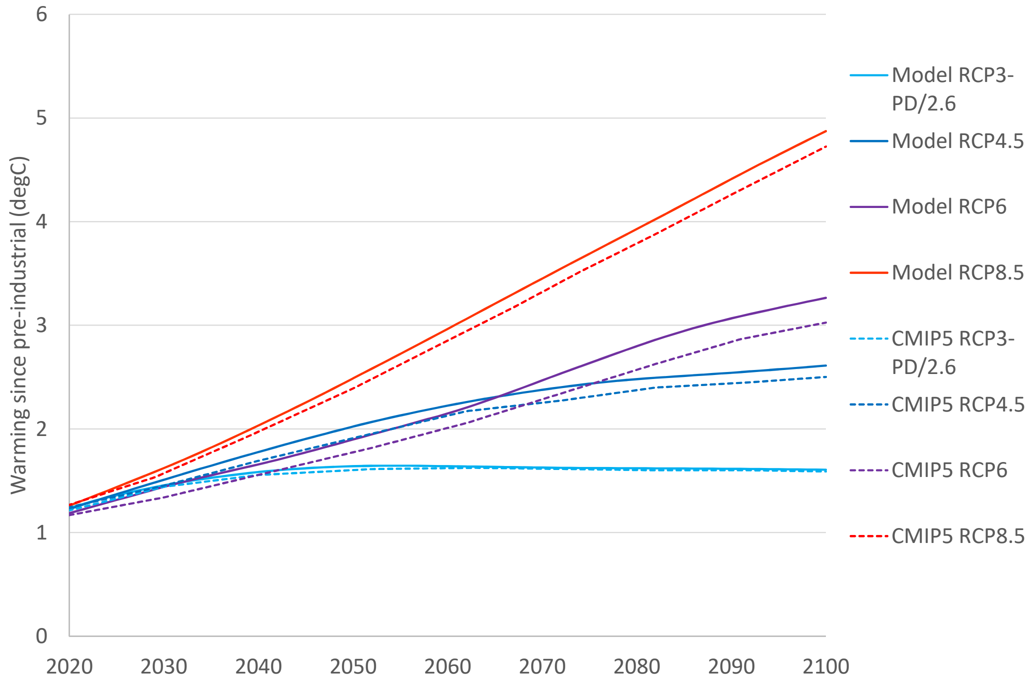

Figure 12 compares 21st century SLR projected by our model with the corresponding multi-model median projection from the IPCC *Fifth Assessment Report* (93). The IPCC projection is based on the set of process-based models. For a like-for-like comparison, our projection is based on the median of a 5,000 run Monte Carlo simulation. The forcing scenario is RCP4.5.

The projections of total SLR from all sources – including thermal expansion, melting of glaciers and small ice sheets, and melting of the GIS and WAIS – are similar. Our model projects 0.009m less SLR in 2020, but 0.06m more in 2100. The difference is well within the 67% confidence interval (93, Figure 13.11). Both the GIS model of (3) and the WAIS

model of (61) project lower SLR contributions than their median IPCC counterparts. The difference is larger for the GIS. The extra SLR projected towards the end of the century in our model compared with the median IPCC model is therefore due to thermal expansion and melting of glaciers and small ice sheets.

Figure 12: Comparison between SLR projections of our model and the median process-based model as reported in (93) for RCP4.5.

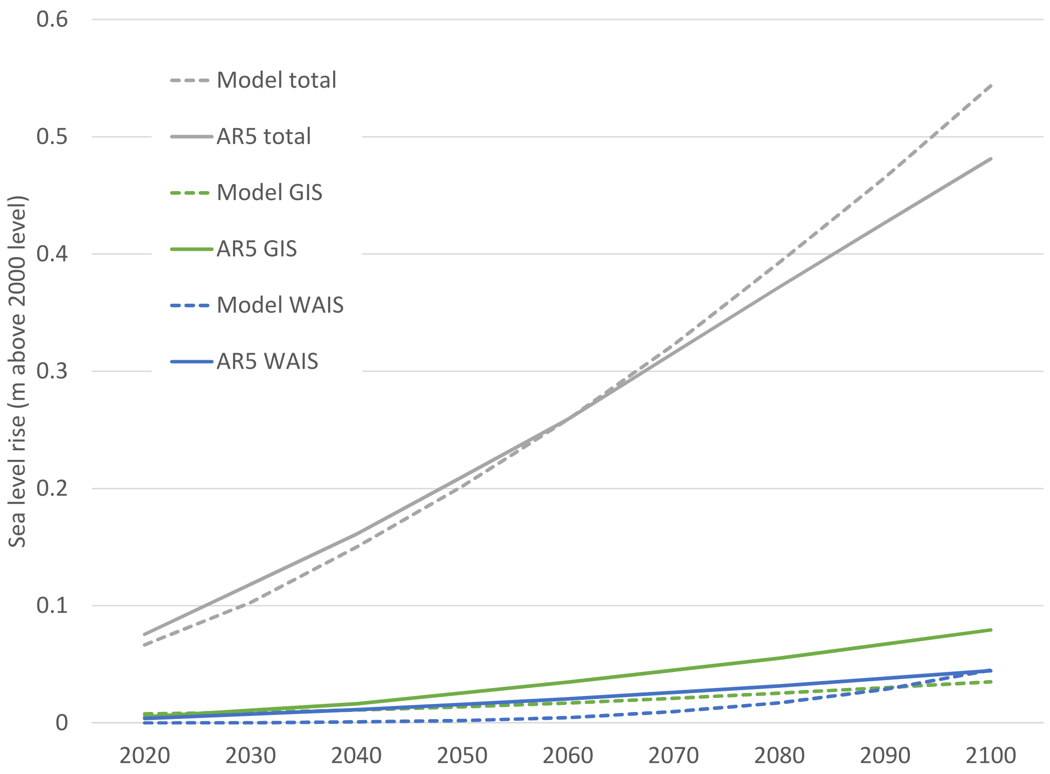

3.2 Sensitivity analysis on SCC

3.2.1 Permafrost carbon feedback

Table 6 and Figure 13 report the change in the SCC due to the permafrost carbon feedback across different scenarios reported in the literature. The central estimates of the three published studies are in relatively close agreement about the percentage increase in the expected SCC, ranging from 6.3% in (57) to 12.4% in (30). In the stochastic implementation of Kessler's (30) model, the distribution of percentage increases in the SCC spreads, but the change in the expected SCC is not much higher than in Kessler's deterministic implementation, at 13.5%.

Table 6: The expected SCC (2020 US$) and the percentage change in the expected SCC due to the permafrost carbon feedback. The expected SCC is computed over 10,000 Monte Carlo draws with 0.1% trimmed. Emissions and GDP/population growth are from RCP4.5-SSP2.

PCF scenario	Without PCF	With PCF	% increase
None	52.03	-	-
Hope and Schaefer (2016)	-	56.01	8.4
Kessler (2017) central value	-	58.48	12.4
Kessler (2017) stochastic	-	59.06	13.5
Yumashev et al. (2019)	-	55.31	6.3

Figure 13: The percentage change in the SCC due to the permafrost carbon feedback. Boxes show median and interquartile range; whiskers show 95% confidence interval; crosses mark the average change (0.1% trimmed); triangles mark the 0.5 percentile; squares mark the 99.5 percentile. Y-axis is truncated. Emissions and GDP/population growth are from RCP4.5-SSP2. Monte Carlo sample size is 10,000.

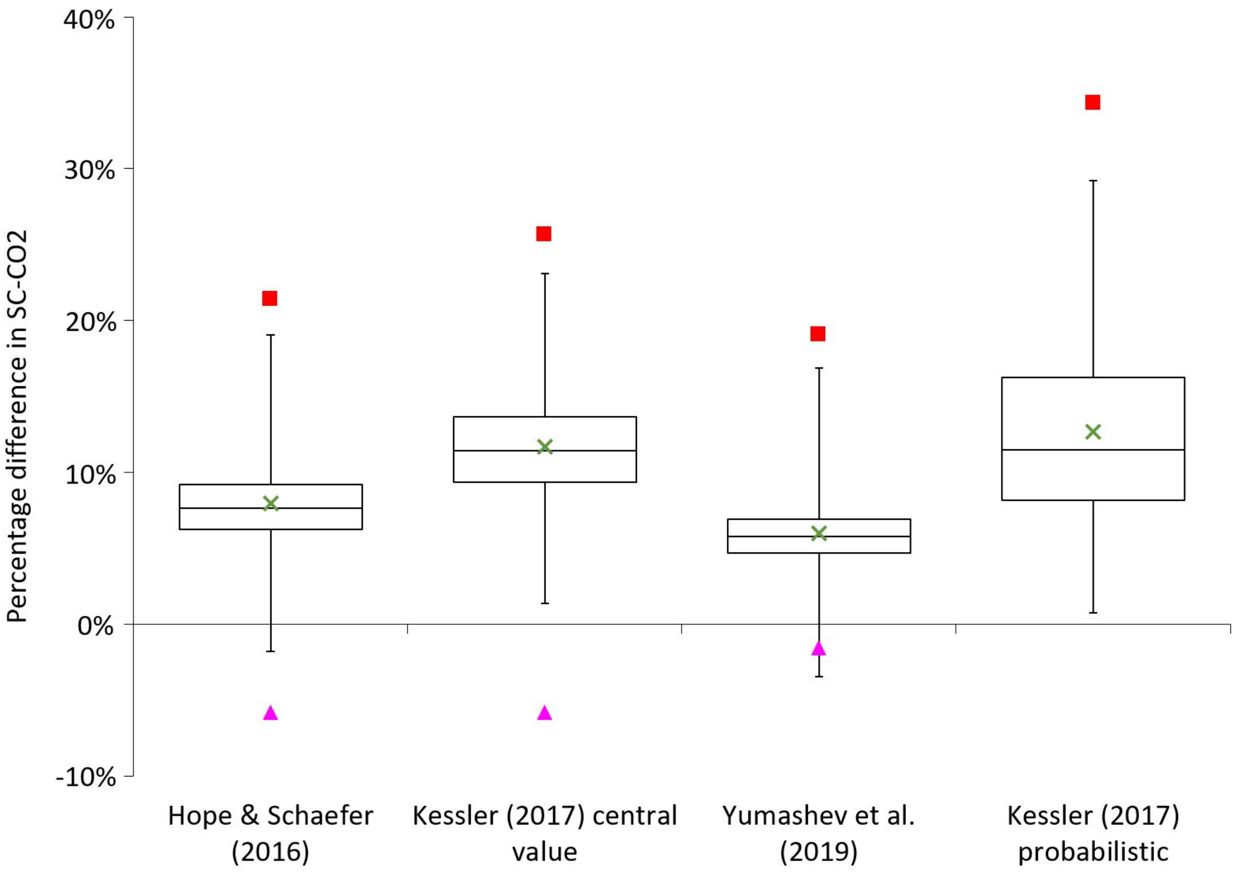

3.2.2 Ocean methane hydrates

Table 7 and Figure 14 report the change in the SCC due to dissociation of ocean methane hydrates across different scenarios reported in the literature. The main scenario explored in (53) and the three scenarios explored in (59) involve very different cumulative CH_4 releases, which is reflected in a wide range of values of the expected SCC, ranging from 4.1% higher than without the tipping point, to 49.2% higher. Conversely the increase in the SCC in the (53) scenario (50GtCH_4 cumulative release) appears very robust to the specification of the hazard rate, as well as the duration of the 50GtCH_4 release (see Section 2.1.2). In

particular, although the hazard rate is much higher under the uniform distribution, the percentage increase in the SCC is only fractionally higher. This reflects the fact that the 50GtCH_4 release is relatively likely before 2200 under all specifications and the difference in the expected timing of the release due to differences in the hazard rate has a relatively small effect on the SCC, as do differences in the duration of the release between 10 and 30 years (default specification is 20 years). This is consistent with the sensitivity analysis performed by (53) using the PAGE2002 IAM.

Table 7: The expected SCC (2020 US$) and the percentage change in the expected SCC due to dissociation of ocean methane hydrates. The expected SCC is computed over 10,000 Monte Carlo draws with 0.1% trimmed. Emissions and GDP/population growth are from RCP4.5-SSP2. The hazard rate b_{OMH} is beta distributed unless otherwise specified.

OMH scenario	Without OMH	With OMH	% increase
None	52.03	-	-
Whiteman et al. (2013)	-	58.85	13.1
Ceronsky et al. (2011) 0.2Gt/yr	-	54.14	4.1
Ceronsky et al. (2011) 1.8Gt/yr	-	63.40	21.9
Ceronsky et al. (2011) 7.8Gt/yr	-	77.62	49.2
Whiteman et al. (2013) uniform	-	59.57	14.5
Whiteman et al. (2013) triangular	-	59.57	14.5
Whiteman et al. (2013) 10 years	-	58.68	12.8
Whiteman et al. (2013) 30 years	-	58.61	12.6

Figure 14: The percentage change in the SCC due to dissociation of ocean methane hydrates. Boxes show median and interquartile range; whiskers show 95% confidence interval; crosses mark the average change (0.1% trimmed); triangles mark the 0.5 percentile; squares mark the 99.5 percentile. Y-axis is truncated. Emissions and GDP/population growth are from RCP4.5-SSP2. The hazard rate b_{OMH} is beta distributed unless otherwise specified. Monte Carlo sample size is 10,000.

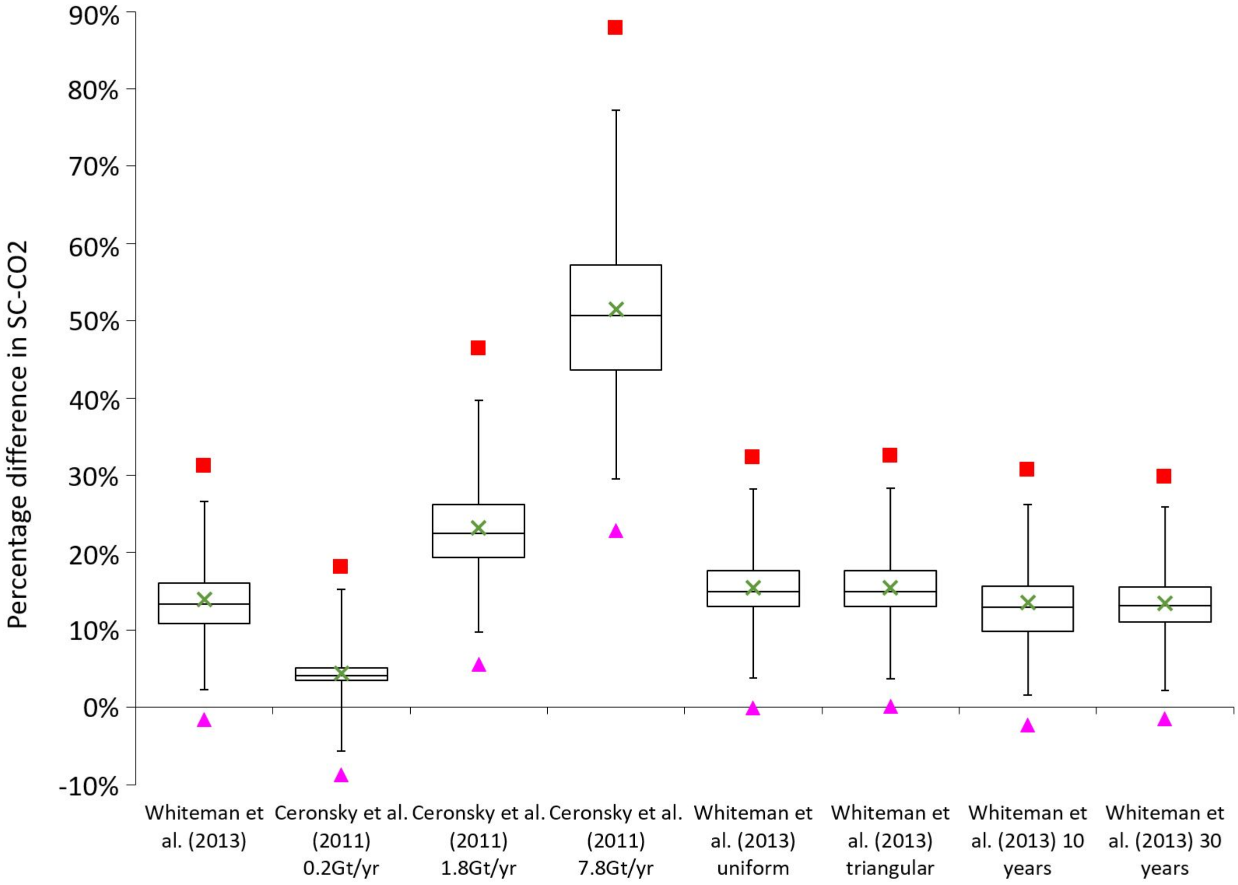

3.2.3 Slowdown of the Atlantic Meridional Overturning Circulation

Table 8 and Figure 15 report the change in the SCC due to slowdown of the AMOC across the four hosing scenarios explored in (6). The SCC is lower across all quantiles in all four scenarios. (6) similarly found an increase in welfare in all four scenarios, using both damage functions fitted on the literature, and the FUND IAM. The decrease in the expected SCC is much larger in the 'Hadley 67%' scenario than in the other scenarios, at -5.4%. This reflects the much larger, two-thirds slowdown of the AMOC in this scenario. Notice that the decrease in the expected SCC is greater in the 'HADCM 7% scenario' than the 'BCM 24%' scenario,

despite a smaller slowdown of the circulation. In general, the ordering of temperature effects of the three milder hosing scenarios varies across countries, so while the slowdown is smallest overall under 'HADCM 7%', the cooling effect is larger in some countries (see 6, Fig. 1).

Table 8: The expected SCC (2020 US$) and the percentage change in the expected SCC due to slowdown of the Atlantic Meridional Overturning Circulation. The expected SCC is computed over 10,000 Monte Carlo draws with 0.1% trimmed. Emissions and GDP/population growth are from RCP4.5-SSP2.

Hosing scenario	Without AMOC	With AMOC	% increase
None	52.03	-	-
BCM 24%	-	51.65	-0.7
HADCM 7%	-	51.59	-0.8
IPSL 27%	-	51.28	-1.4
Hadley 67%	-	49.06	-5.7

Figure 15: The percentage change in the SCC due to slowdown of the Atlantic Meridional Overturning Circulation. Boxes show median and interquartile range; whiskers show 95% confidence interval; crosses mark the average change (0.1% trimmed); triangles mark the 0.5 percentile; squares mark the 99.5 percentile. Emissions and GDP/population growth are from RCP4.5-SSP2. Monte Carlo sample size is 10,000.

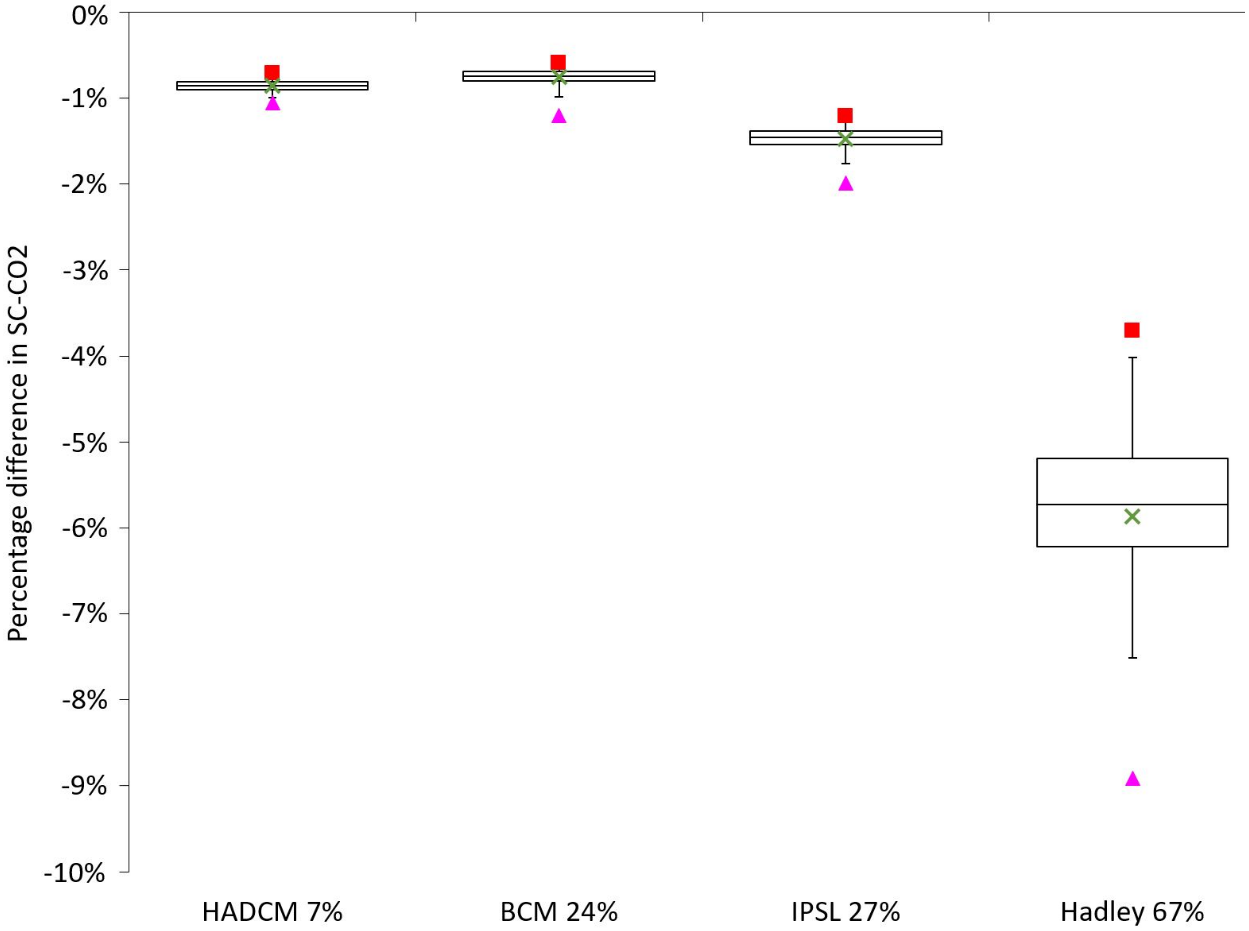

3.2.4 Emissions/socio-economic scenario

Table 9 and Figure 16 report the change in the SCC due to tipping points in five different paired RCP/SSP emissions/socio-economic scenarios. Overall, the increase in the expected SCC ranges from 15.4% to 33.8%. The expected SCC is highest in the RCP6-SSP4 scenario, which has the second highest GHG emissions from the middle of the 21st century onwards, and is characterised by rapid population growth and low income per capita in currently poor regions, amplifying the welfare costs of climate change. The effect of tipping points on the expected SCC is highest in the RCP3-PD/2.6-SSP1 scenario, which is the lowest emissions

scenario, consistent with limiting global warming to below 2°C in the absence of tipping points (see Section 3.1). This is paired with SSP1, which is characterised by generally low population growth and rapid growth in incomes.

The role of income and population projections is evident when comparing RCP4.5-SSP2 with RCP4.5-SSP5. This comparison holds emissions constant while varying income and population. SSP5 has higher income per capita than SSP2, and lower population. The expected SCC is hence lower on RCP4.5-SSP5, in fact it is lowest of all. The effect of tipping points on the SCC is about the same, however. Comparing RCP4.5-SSP5 with RCP8.5-SSP5 holds income and population constant while varying emissions. The expected SCC is hence higher on the high emissions RCP8.5 scenario.[23]

Table 9: The expected SCC (2020 US$) and the percentage change in the expected SCC due to all tipping points combined in four different paired RCP/SSP emissions/socio-economic scenarios. The expected SCC is computed over 10,000 Monte Carlo draws with 0.1% trimmed. Specification comprises: Hope and Schaefer PCF; Whiteman et al. beta OMH; IPSL AMOC hosing.

RCP-SSP	Without TPs	With all TPs	% increase
RCP3-PD/2.6, SSP1	33.96	45.42	33.8
RCP4.5, SSP2	52.03	64.80	24.5
RCP6, SSP4	80.55	92.91	15.4
RCP8.5, SSP5	32.85	39.23	19.4
RCP4.5, SSP5	23.12	28.90	25.0

[23]Not all combinations of RCP and SSP are considered possible. In particular, RCP8.5 is only considered compatible with SSP5 (94).

Figure 16: The percentage change in the SCC due to all tipping points combined in four different paired RCP/SSP emissions/socio-economic scenarios. Boxes show median and interquartile range; whiskers show 95% confidence interval; crosses mark the average change (0.1% trimmed); triangles mark the 0.5 percentile; squares mark the 99.5 percentile. Y-axis is truncated. Specification comprises: Hope and Schaefer PCF; Whiteman et al. beta OMH; IPSL AMOC hosing. Monte Carlo sample size is 10,000.

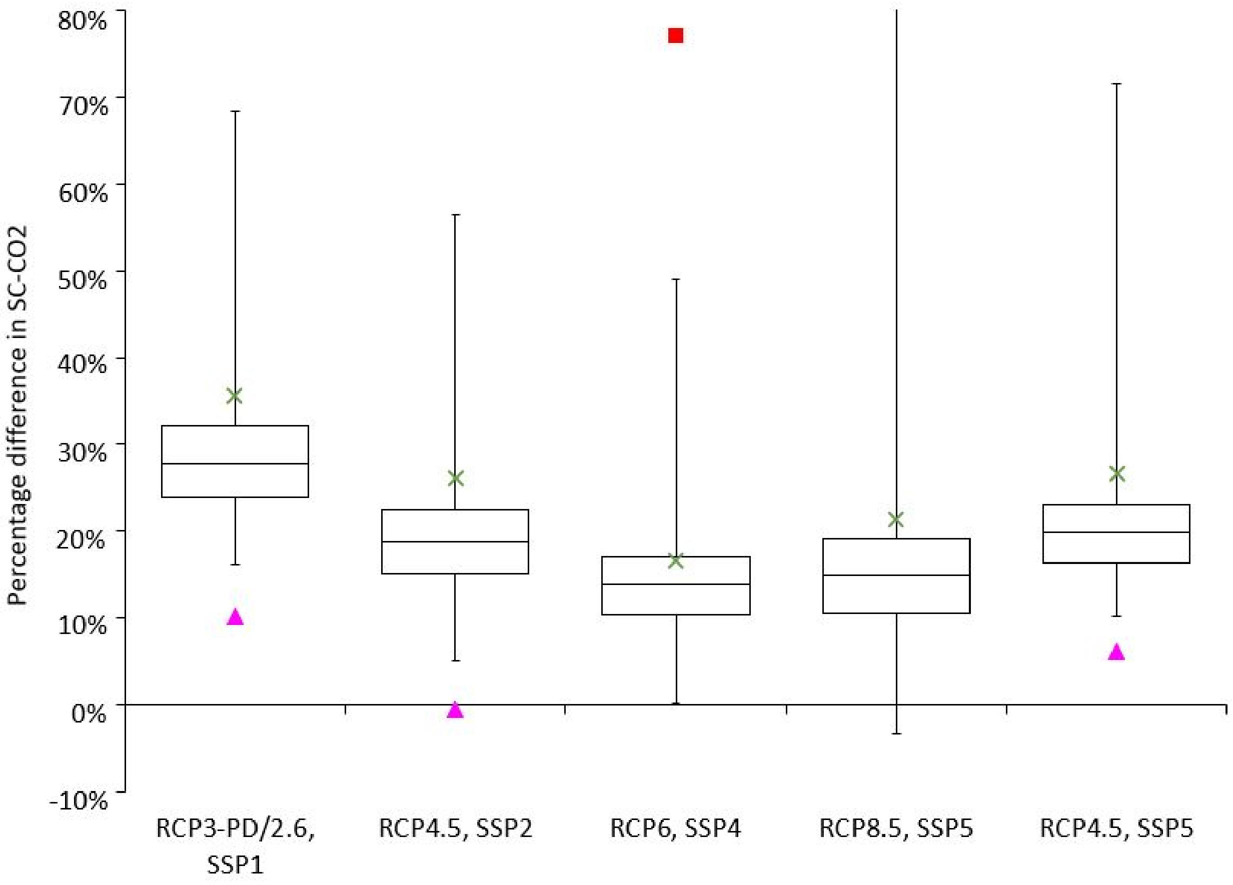

3.2.5 Levels versus growth damages

Table 10 and Figure 17 report the change in the SCC due to tipping points for different values of the parameter φ that determines whether climate damages work on the level or growth rate of income. Our main specification is an intermediate value of $\varphi = 0.5$. When $\varphi = 1$, representing pure levels damages, the expected SCC is lower at \$26.94/tCO_2, while the percentage increase in the expected SCC is slightly higher at 26.0%. As φ is increased across its range, the SCC increases, but the percentage increase in the expected SCC due to

tipping points exhibits a small decrease. However, with a pure growth damages specification ($\varphi = 0$), the expected SCC is nearly a factor of 100 higher and the percentage increase in the expected SCC is also much higher, at 87.0%. Thus both the expected SCC and the effect of tipping points on it are highly sensitive to φ as it approaches its lower limit value of zero. The high value of the expected SCC when $\varphi = 0$ is in the ballpark of those values reported by (95), who also used a pure growth damages specification.

Table 10: The expected SCC (2020 US$) and the percentage change in the expected SCC due to all tipping points combined for different values of the parameter φ. The expected SCC is computed over 10,000 Monte Carlo draws with 0.1% trimmed. Specification comprises: RCP4.5-SSP2 emissions and GDP/population growth; Hope and Schaefer PCF; Whiteman et al. beta OMH; IPSL AMOC hosing.

φ	Without TPs	With all TPs	% increase
0, pure growth damages	3468.74	6488.19	87.0
0.1	219.29	267.36	21.9
0.2	121.16	149.00	23.0
0.3	83.84	103.67	23.7
0.4	64.17	79.67	24.1
0.5, main spec.	52.03	64.80	24.5
0.6	43.79	54.69	24.9
0.7	37.83	47.36	25.2
0.8	33.32	41.81	25.5
0.9	29.78	37.45	25.7
1, pure levels damages	26.94	33.94	26.0

Figure 17: The percentage change in the SCC due to all tipping points combined for different values of the parameter φ. Boxes show median and interquartile range; whiskers show 95% confidence interval; crosses mark the average change (0.1% trimmed); triangles mark the 0.5 percentile; squares mark the 99.5 percentile. Y-axis is truncated. Specification comprises: RCP4.5-SSP2 emissions and GDP/population growth; Hope and Schaefer PCF; Whiteman et al. beta OMH; IPSL AMOC hosing. Monte Carlo sample size is 10,000.

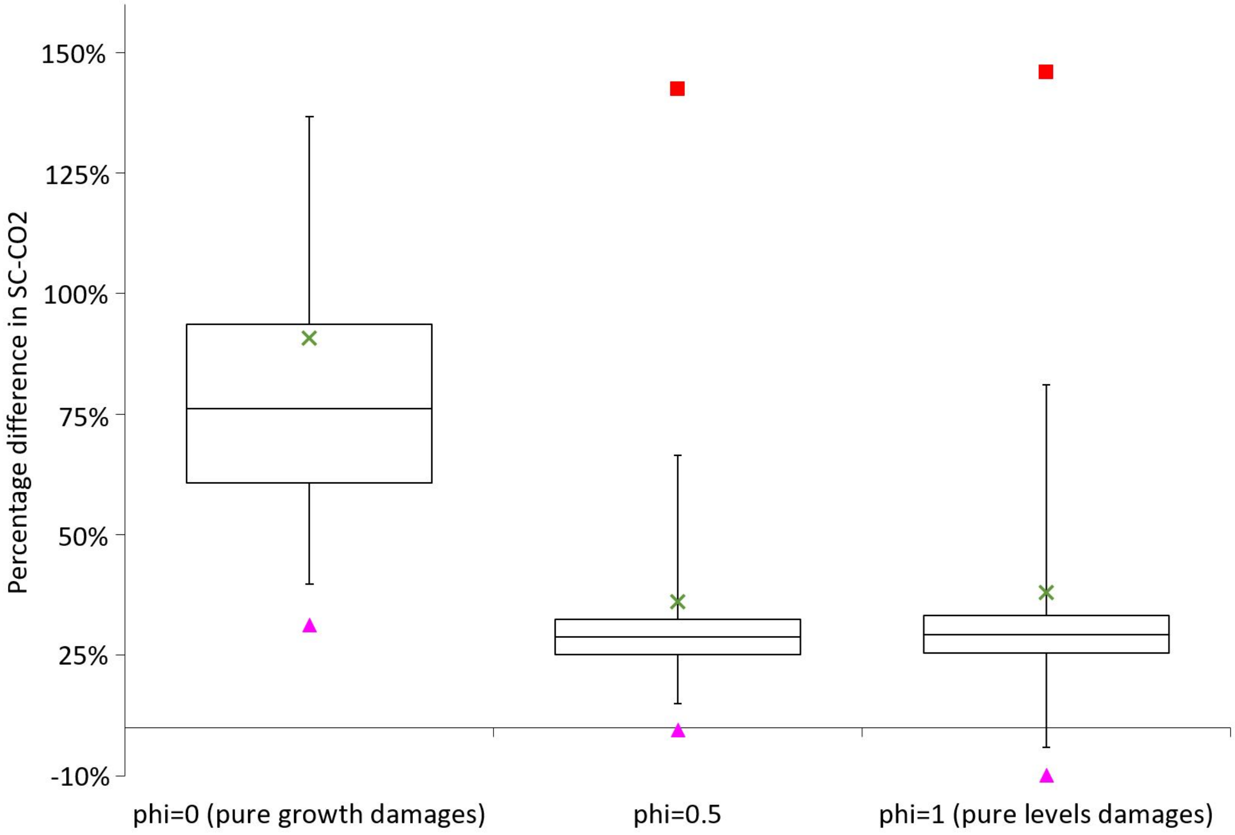

3.2.6 Pure rate of time preference

Table 11 and Figure 18 report the change in the SCC due to tipping points for different values of the pure rate of time preference. A low pure rate of time preference of 0.1%, assumed by (96) for instance, results in a significantly higher expected SCC, but tipping points have a similar effect on the expected SCC compared with our main specification. A high pure rate of time preference of 2% results in a smaller expected SCC, but tipping points again have a similar effect on the expected SCC.

Table 11: The expected SCC (2020 US$) and the percentage change in the expected SCC due to all tipping points combined for different values of the pure rate of time preference. The expected SCC is computed over 10,000 Monte Carlo draws with 0.1% trimmed. Specification comprises: RCP4.5-SSP2 emissions and GDP/population growth; Hope and Schaefer PCF; Whiteman et al. beta OMH; IPSL AMOC hosing.

Pure rate of time preference (%)	Without TPs	With all TPs	% increase
0.1	92.388	113.02	22.3
1, main spec.	52.03	70.20	24.5
2	32.67	41.26	26.3

Figure 18: The percentage change in the SCC due to all tipping points combined for different values of the pure rate of time preference. Boxes show median and interquartile range; whiskers show 95% confidence interval; crosses mark the average change (0.1% trimmed); triangles mark the 0.5 percentile; squares mark the 99.5 percentile. Y-axis is truncated. Specification comprises: RCP4.5-SSP2 emissions and GDP/population growth; Hope and Schaefer PCF; Whiteman et al. beta OMH; IPSL AMOC hosing. Monte Carlo sample size is 10,000.

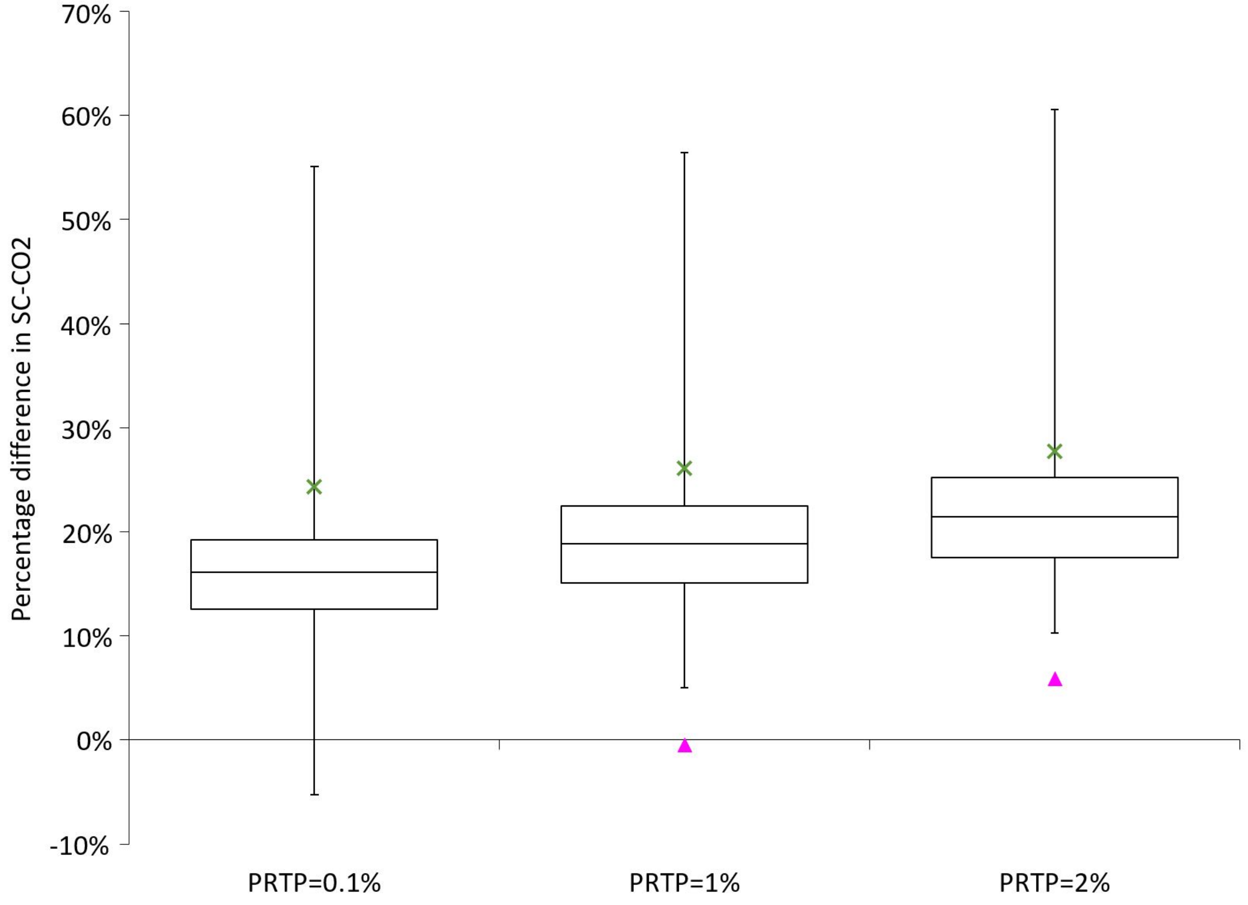

3.2.7 Elasticity of marginal utility of consumption

Table 12 and Figure 19 report the change in the SCC due to tipping points for different values of the elasticity of marginal utility of consumption. Varying the elasticity of marginal utility has three separate effects in models like ours that have not only (i) a time dimension, but (ii) are also spatially disaggregated and (iii) incorporate risk. By determining the value of consumption per capita in each country i at time t in different states of nature, the elasticity of marginal utility modulates (i) temporal inequality aversion, (ii) spatial inequality aversion and (iii) risk aversion. This explains why the elasticity of marginal utility has non-monotonic effects on the expected SCC, as Table 12 shows. That is, the expected SCC is higher both when the elasticity parameter is lower than our main specification and when it is higher. The effect of tipping points on the expected SCC is similar when the elasticity parameter is set to a very low value of 0.5, however when it is set to 2 the effect of tipping points is much larger, at 55.8%. Further inspection of Figure 19 shows that this large percentage difference in the expected SCC when the elasticity of marginal utility is 2 is driven by a few runs in the tail of the distribution, since the median percentage difference in the SCC due to tipping points is just 22.0%. The sensitivity of welfare estimates to the elasticity of marginal utility in cases where consumption per capita can be driven to near-subsistence levels is well known, having been identified by Weitzman as part of his 'Dismal Theorem' (97; 98; 99).

Table 12: The expected SCC (2020 US$) and the percentage change in the expected SCC due to all tipping points combined for different values of the elasticity of marginal utility of consumption. The expected SCC is computed over 10,000 Monte Carlo draws with 0.1% trimmed. Specification comprises: RCP4.5-SSP2 emissions and GDP/population growth; Hope and Schaefer PCF; Whiteman et al. beta OMH; IPSL AMOC hosing.

Elasticity of marginal utility of consumption	Without TPs	With all TPs	% increase
0.5	105.14	128.27	22.0
1.5, main spec.	52.03	64.80	24.5
2	98.98	156.59	58.2

Figure 19: The percentage change in the SCC due to all tipping points combined for different values of the elasticity of marginal utility of consumption. Boxes show median and interquartile range; whiskers show 95% confidence interval; crosses mark the average change (0.1% trimmed); triangles mark the 0.5 percentile; squares mark the 99.5 percentile. Y-axis is truncated. Specification comprises: RCP4.5-SSP2 emissions and GDP/population growth; Hope and Schaefer PCF; Whiteman et al. beta OMH; IPSL AMOC hosing. Monte Carlo sample size is 10,000.

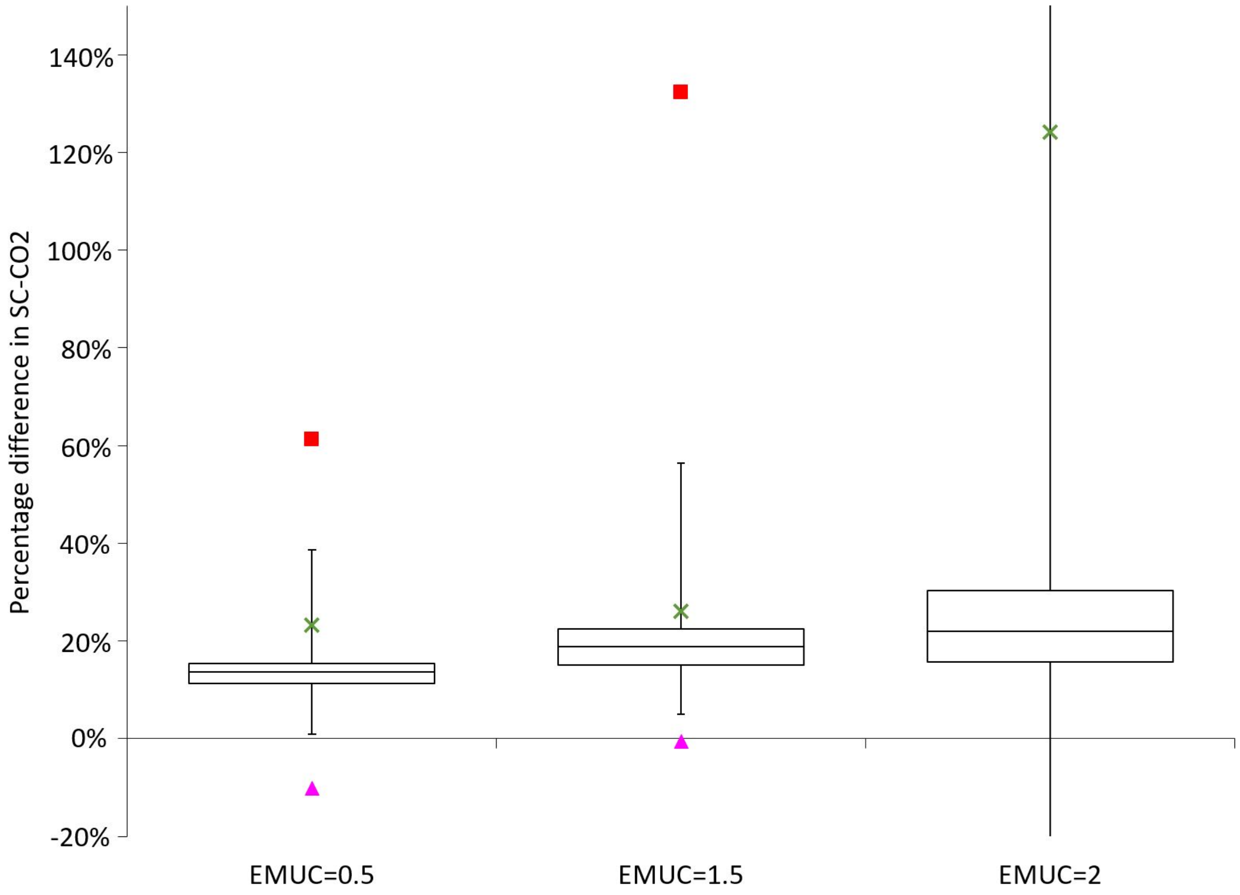

3.2.8 Non-market damages

Table 13 and Figure 20 report the change in the SCC due to tipping points when a globally aggregated, non-market damage function is added to the model, and compares it with the main specification that excludes non-market damages. The expected SCC increases to \$72.76/tCO_2 in the absence of tipping points. The effect of switching on the tipping points is to increase the expected SCC by 26.9%, which constitutes a slight increase compared with market damages only. The figure illustrates that adding non-market damages condenses the

distribution of percentage increases, although both distributions have a positive skew.

Table 13: The expected SCC (2020 US$) and the percentage change in the expected SCC due to all tipping points combined, with and without non-market damages. The expected SCC is computed over 10,000 Monte Carlo draws with 0.1% trimmed. Specification comprises: RCP4.5-SSP2 emissions and GDP/population growth; Hope and Schaefer PCF; Whiteman et al. beta OMH; IPSL AMOC hosing.

Damages	Without TPs	With all TPs	% increase
Without non-market	52.03	64.80	24.5
With non-market	72.76	92.35	26.9

Figure 20: The percentage change in the SCC due to all tipping points combined, with and without non-market damages. Boxes show median and interquartile range; whiskers show 95% confidence interval; crosses mark the average change (0.1% trimmed); triangles mark the 0.5 percentile; squares mark the 99.5 percentile. Y-axis is truncated. Specification comprises: RCP4.5-SSP2 emissions and GDP/population growth; Hope and Schaefer PCF; Whiteman et al. beta OMH; IPSL AMOC hosing. Monte Carlo sample size is 10,000.

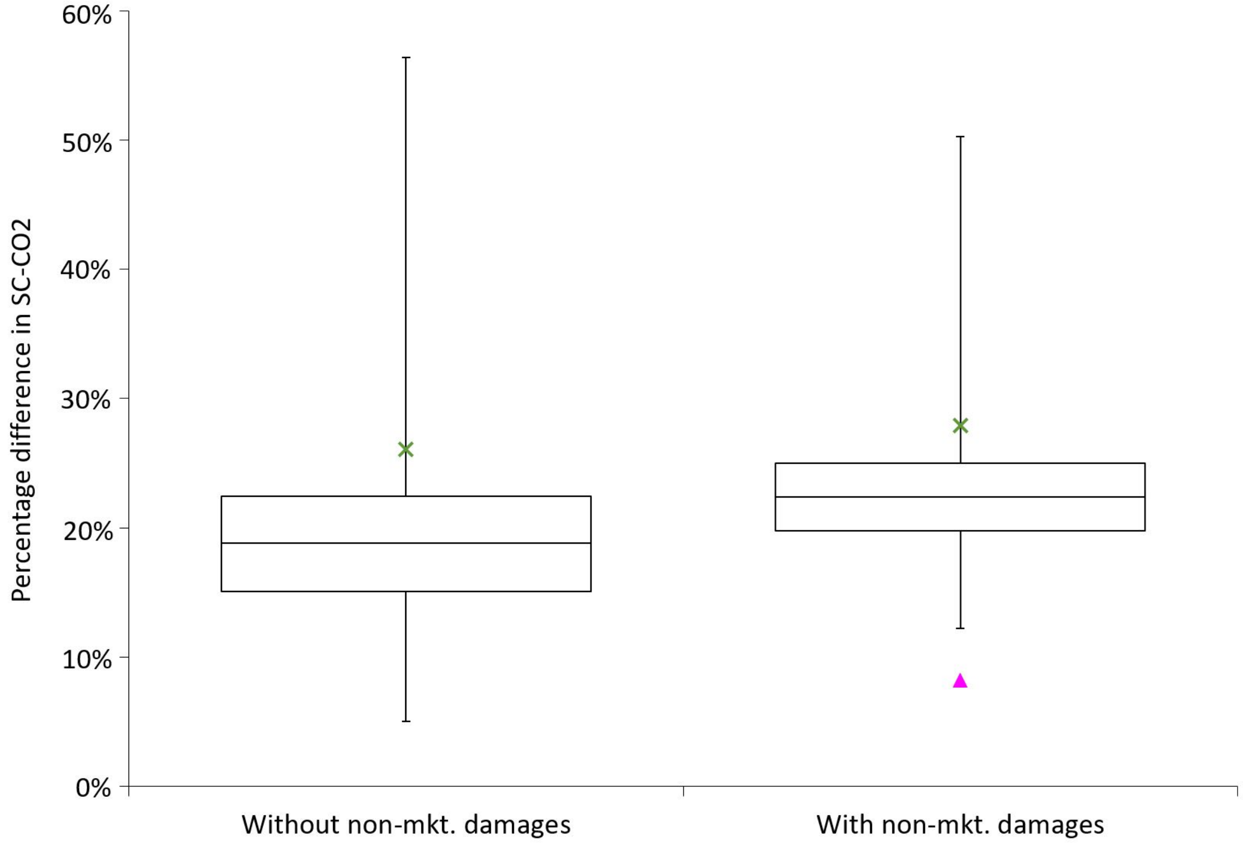

3.2.9 Estimating a confidence interval for the effect of tipping points on the SCC

We would like to estimate the expected change in the SCC and an accompanying confidence interval, which encompass all the different parameter and scenario variations discussed in this section. In order to do so, we take two steps. First, we specify probability distributions for the scalar parameters φ, the pure rate of time preference and the elasticity of marginal utility of consumption.[24] Similarly, to capture uncertainty about the PCF, we adopt Kessler's (30) stochastic settings. Second, we pool Monte Carlo simulations undertaken under each of a large number of combinations of the remaining scenarios that cannot be modelled probabilistically due to the model structure. However, there are too many scenarios for us to be able to explore all scenario combinations. The full factorial combination of all four main OMH scenarios (i.e. the first four rows of Table 7), four AMOC scenarios, four emissions/socio-economic scenarios and two non-market damages scenarios (off/on) comprises 128 Monte Carlo simulations. In order to reduce the number of combinations to a manageable level, while obtaining unbiased estimates of the expected change in the SCC and the confidence interval, we make use of a fractional factorial design. In particular, we use the software package SPSS to draw an orthogonal fraction of resolution IV, which reduces the full factorial problem to 16 scenario combinations, while still being able to capture not only the main effects of each uncertainty dimension but also all two-way interactions between them. For each combination, we run a Monte Carlo simulation with 2000 draws, resulting in a pooled sample of 32,000 draws.

Figure 21 plots the frequency distribution of percentage changes in the SCC across the pooled sample and reports summary statistics including the average, i.e. the expected change. The distribution has a large positive skew. Most draws return a percentage change of between 0% and +50%, but a long tail of draws returns increases of 200% or more. The 95% confidence

[24] φ is uniformly distributed between 0 and 1, the pure rate of time preference is triangular distributed with a minimum of 0.1%, a mode of 1% and a maximum of 2%, and the elasticity of marginal utility is also triangular distributed with a minimum of 0.5, mode of 1.5 and maximum of 2.

interval is -0.3-186.0%. Eleven per cent of draws result in the SCC at least doubling. The expected change in the SCC is 42.8%.

Figure 21: Frequency distribution of percentage changes in the SCC due to all tipping points combined, based on pooled sample of 32,000 Monte Carlo draws from a fractional factorial design.

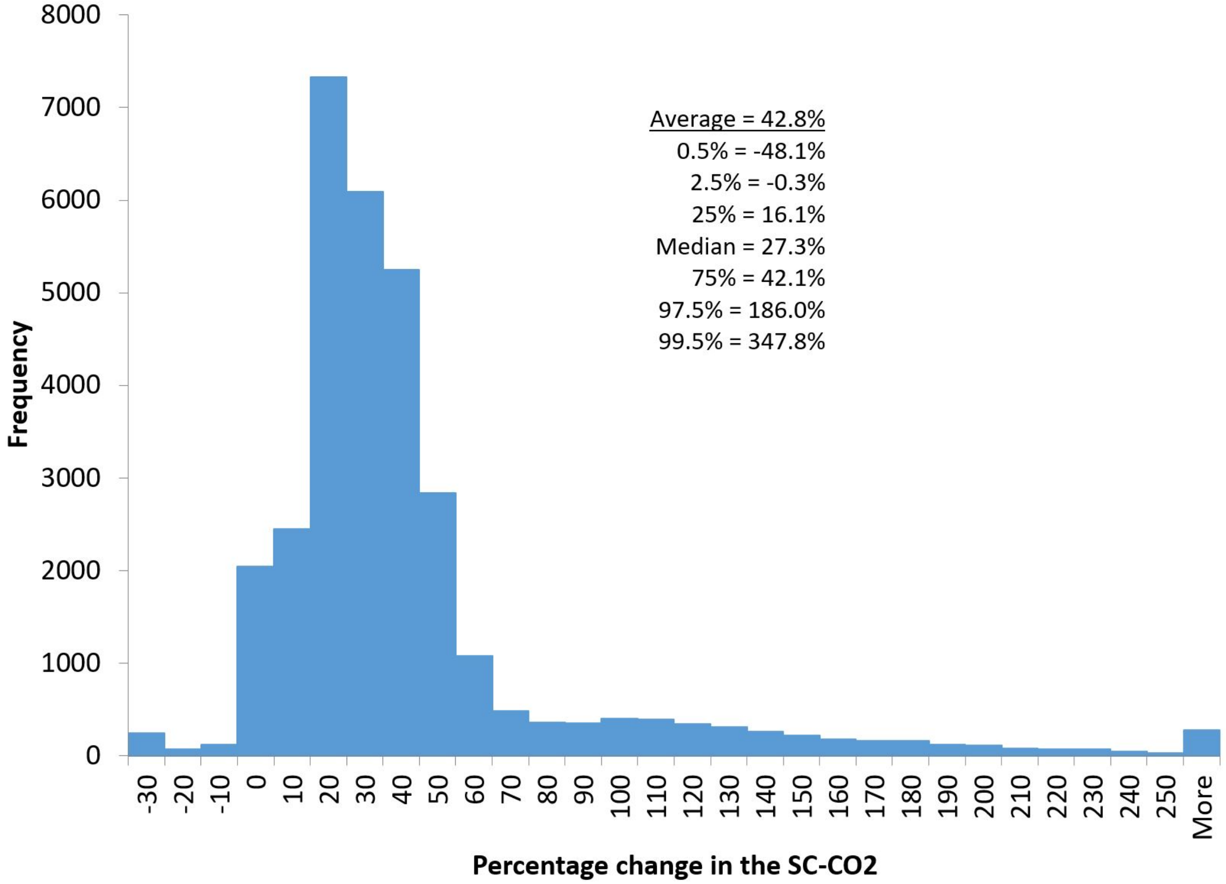

3.2.10 Savings rates

Table 14 and Figure 22 report the change in the SCC due to tipping points for different country savings rates. Recall that savings rates are exogenous and time-invariant, and in our main specification they are calibrated on mean country-specific savings rates between 2005 and 2015 according to World Bank data. As a simple sensitivity analysis, we shift each country's (time-invariant) savings rate up and down by two percentage points. Two percentage points represents roughly two standard deviations of the global average savings

rate between 2005 and 2015. The expected SCC is lower in both cases. The effect of tipping points on the expected SCC is close to our main specification.

Table 14: The expected SCC (2020 US$) and the percentage change in the expected SCC due to all tipping points combined for different country savings rates. The expected SCC is computed over 10,000 Monte Carlo draws with 0.1% trimmed. Specification comprises: RCP4.5-SSP2 emissions and GDP/population growth; Hope and Schaefer PCF; Whiteman et al. beta OMH; IPSL AMOC hosing.

Country savings rates	Without TPs	With all TPs	% increase
-2 percentage points	38.27	46.92	22.6
main spec.	52.03	64.80	24.5
+2 percentage points	36.14	44.31	22.6

Figure 22: The percentage change in the SCC due to all tipping points combined for different country savings rates. Boxes show median and interquartile range; whiskers show 95% confidence interval; crosses mark the average change (0.1% trimmed); triangles mark the 0.5 percentile; squares mark the 99.5 percentile. Y-axis is truncated. Specification comprises: RCP4.5-SSP2 emissions and GDP/population growth; Hope and Schaefer PCF; Whiteman et al. beta OMH; IPSL AMOC hosing. Monte Carlo sample size is 10,000.

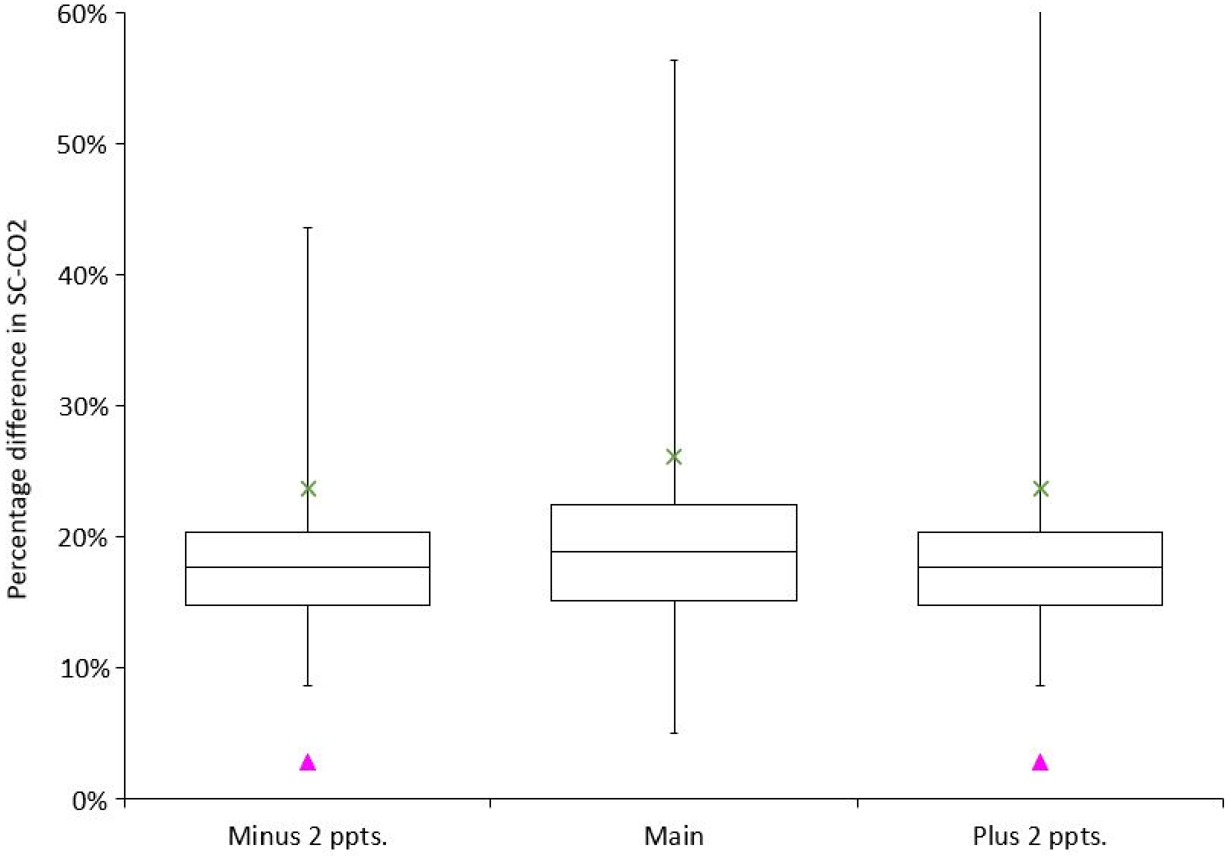

3.3 Spatial impacts of tipping points

This section maps how each individual tipping point affects the country-level SCC. For each figure, we normalise welfare changes to global mean consumption per capita to enable comparison with other SCC estimates in the literature. The specification further comprises RCP4.5-SSP2 emissions and GDP/population growth, and relies on a Monte Carlo sample size of 10,000. As before, we trim 0.1% of runs to ensure consistency.

There is a broad North-South divide in the effect of the PCF (Figure 23), reflecting whether the country experiencing an increase in temperature due to this tipping point is below or above the inflection point in its quadratic temperature-damages relationship (83). Some countries in the North do experience a large increase in their SCC, however, due to the effect of the PCF on SLR (e.g. France and the UK). Dissociation of OMH gives rise to a larger and more immediate increase in temperature, which pushes almost all countries beyond their inflection point resulting in a more uniform pattern (Figure 24). The effects of disintegration of the GIS and WAIS are similar (Figures 26 and 27 respectively), reflecting patterns of country exposure to SLR. These relative effects are not associated with latitude. The SAF has almost the inverse effect on country SCCs to the PCF (Figure 28), reflecting its negative effect on warming relative to constant equilibrium climate sensitivity (see below, Section 3.4). AMOC slowdown reduces the SCC in most, but not all, countries. The negative effect is strongest in Europe (Figure 29). The effects of the variability of the Indian Summer Monsoon are felt in India by construction of the model.

Figure 23: Percentage change in the expected country-level SCC due to the permafrost carbon feedback (specification: Hope and Schaefer).

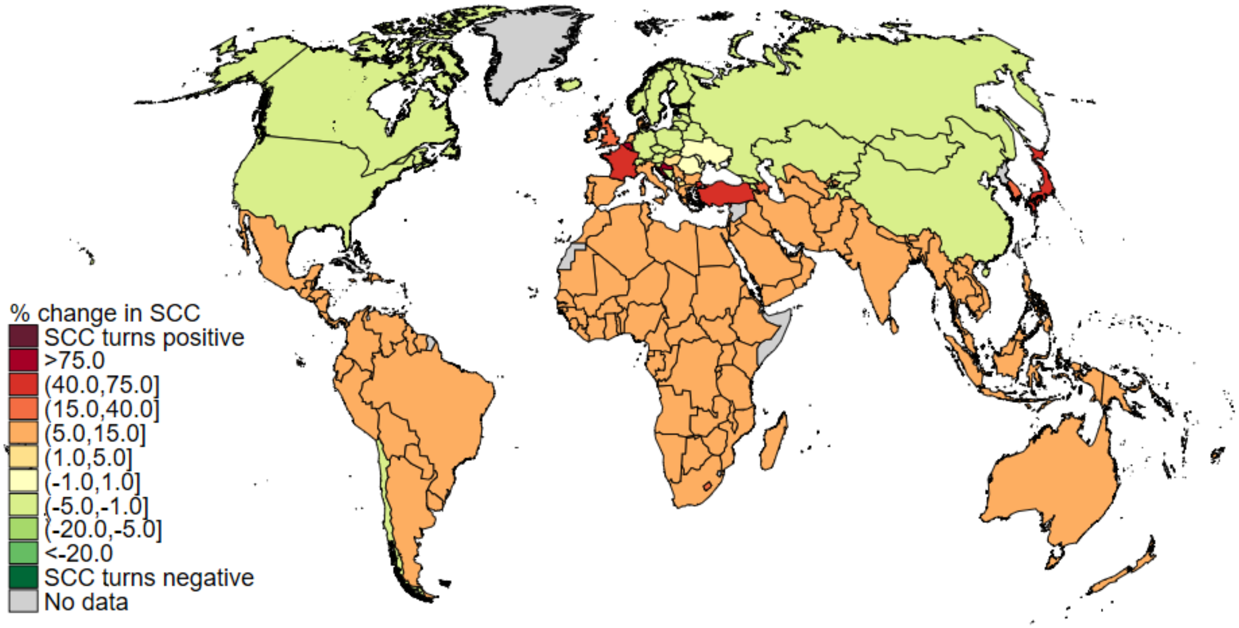

Figure 24: Percentage change in the expected country-level SCC due to the dissociation of ocean methane hydrates (specification: Whiteman et al. beta).

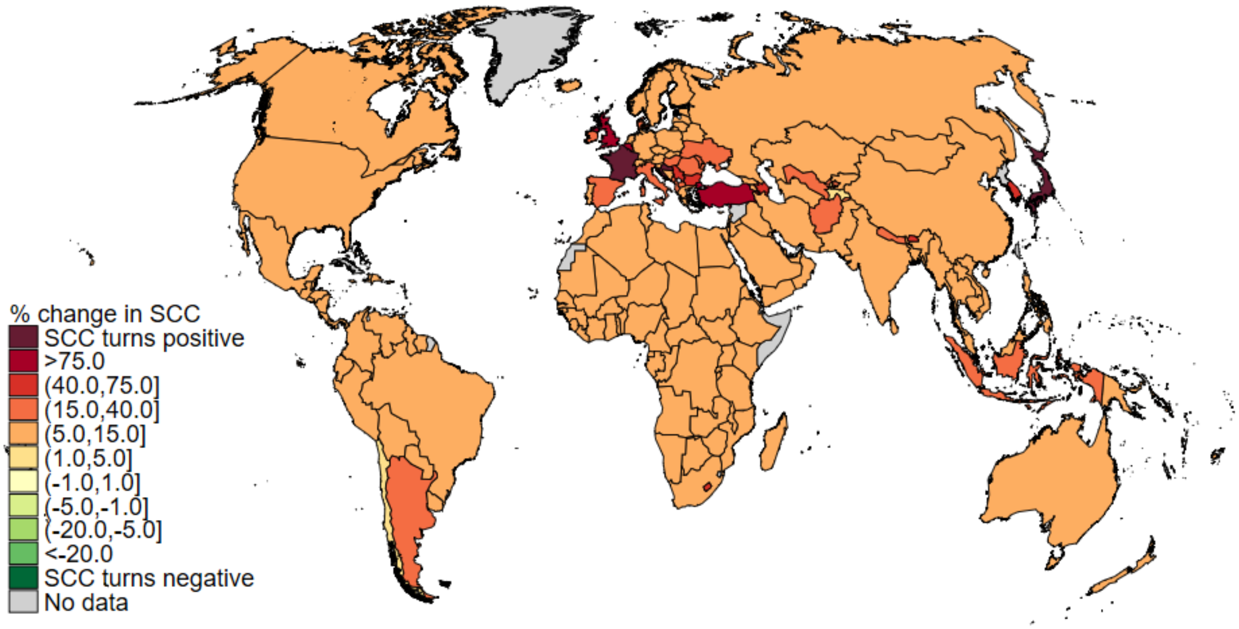

Figure 25: Percentage change in the expected country-level SCC due to Amazon dieback.

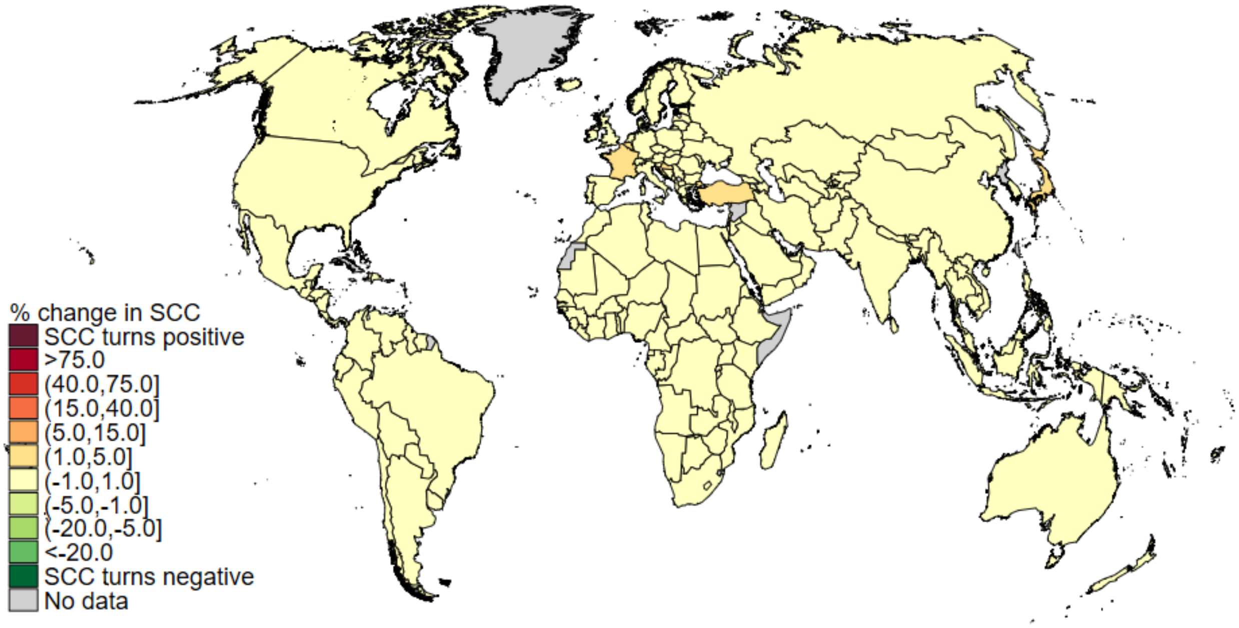

Figure 26: Percentage change in the expected country-level SCC due to disintegration of the Greenland Ice Sheet.

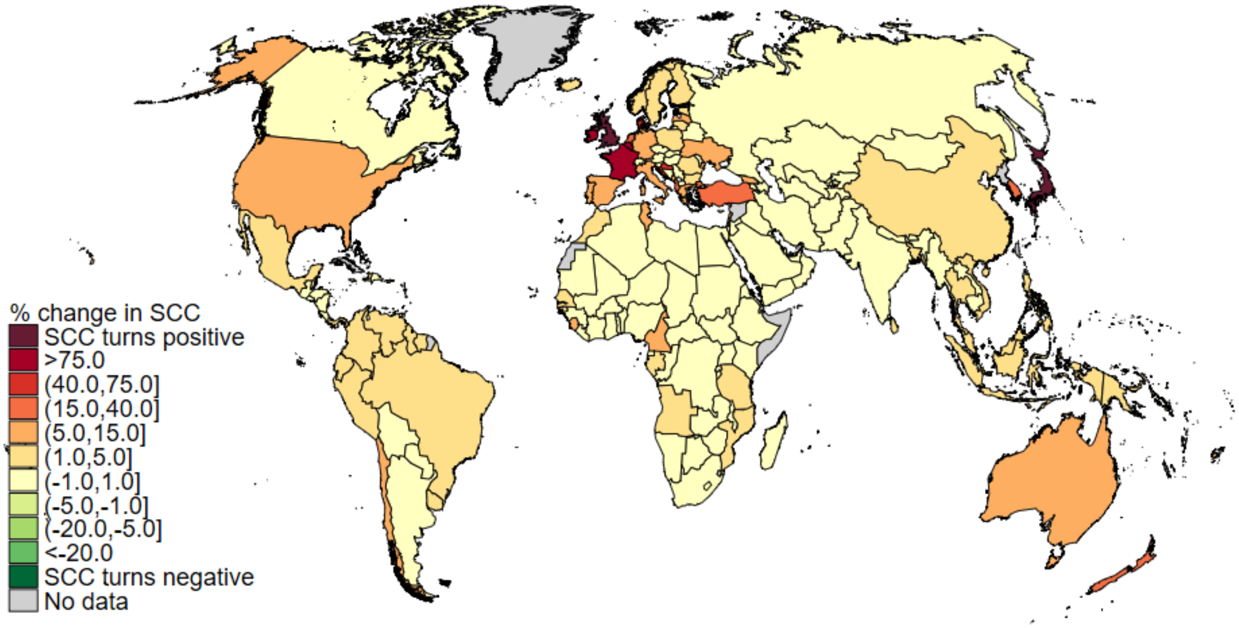

Figure 27: Percentage change in the expected country-level SCC due to disintegration of the West Antarctic Ice Sheet.

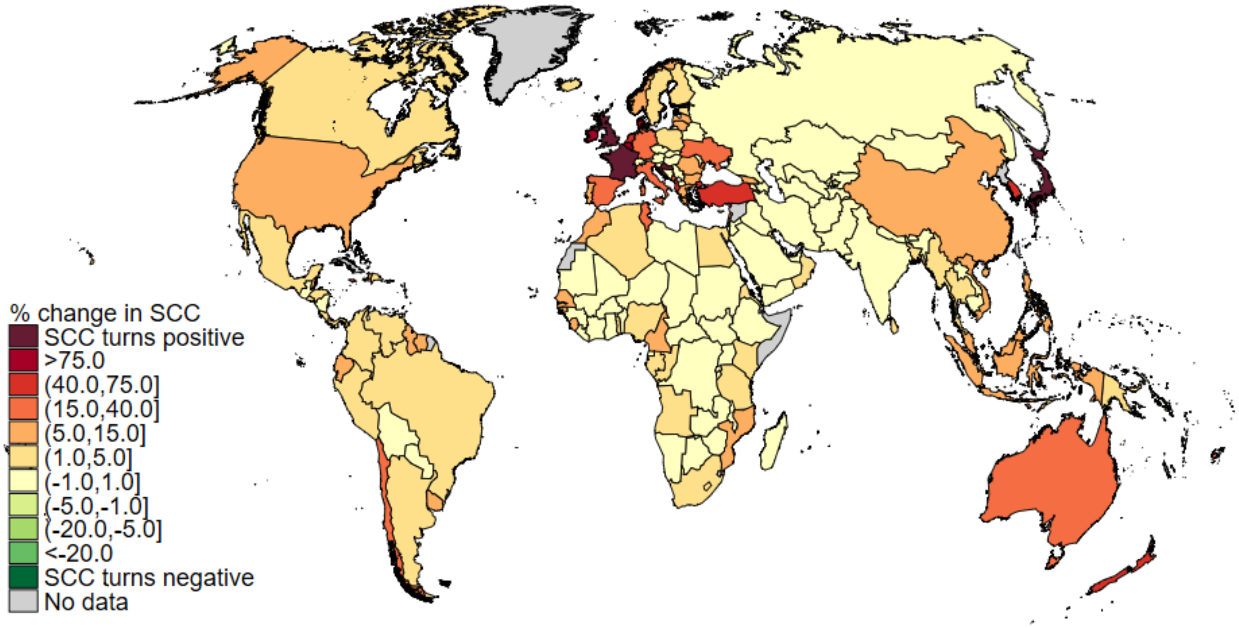

Figure 28: Percentage change in the expected country-level SCC due to the Surface Albedo Feedback.

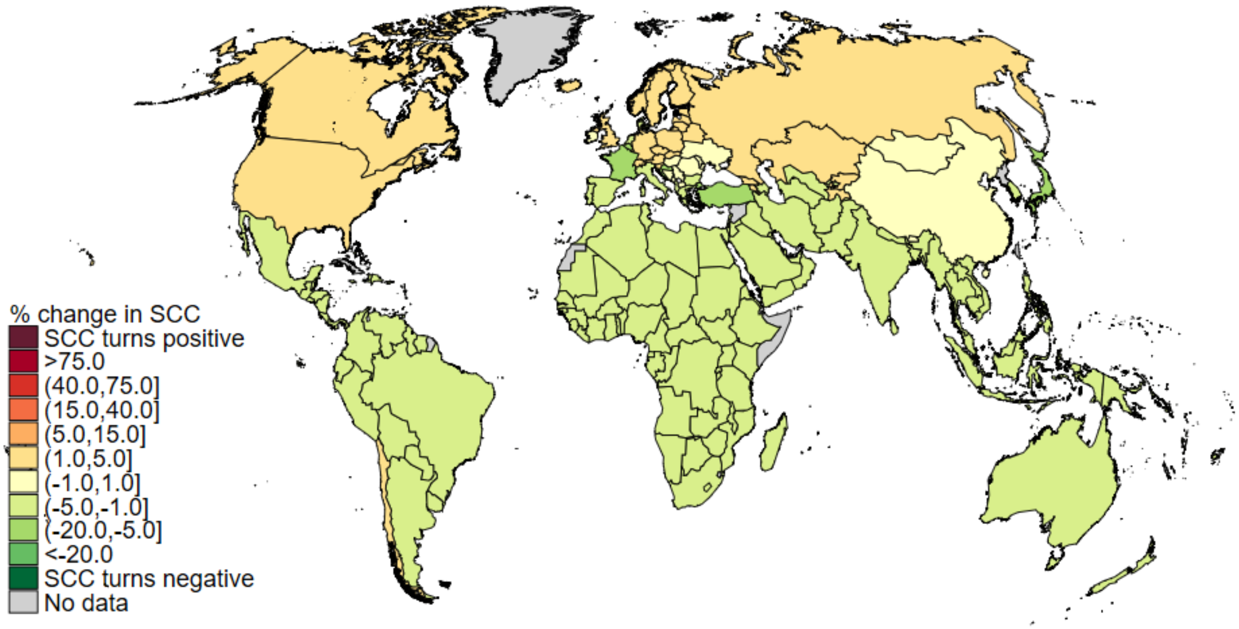

Figure 29: Percentage change in the expected country-level SCC due to weakening of the Atlantic Meridional Overturning Circulation (specification: IPSL hosing).

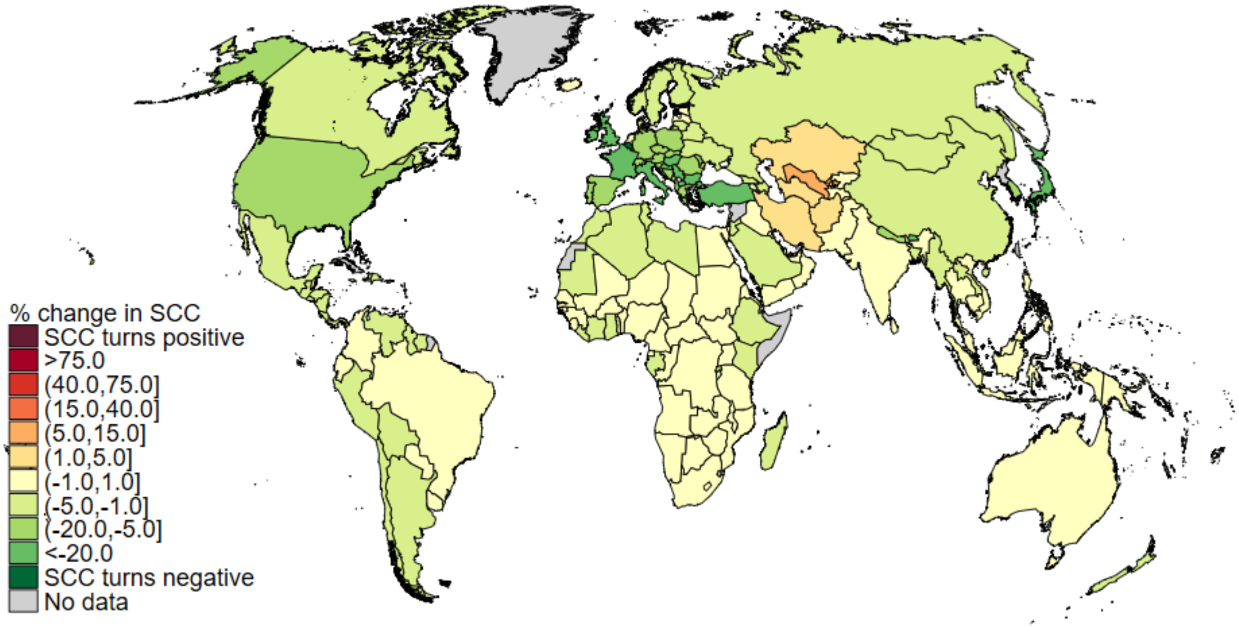

Figure 30: Percentage change in the expected country-level SCC due to variability in the Indian Summer Monsoon.

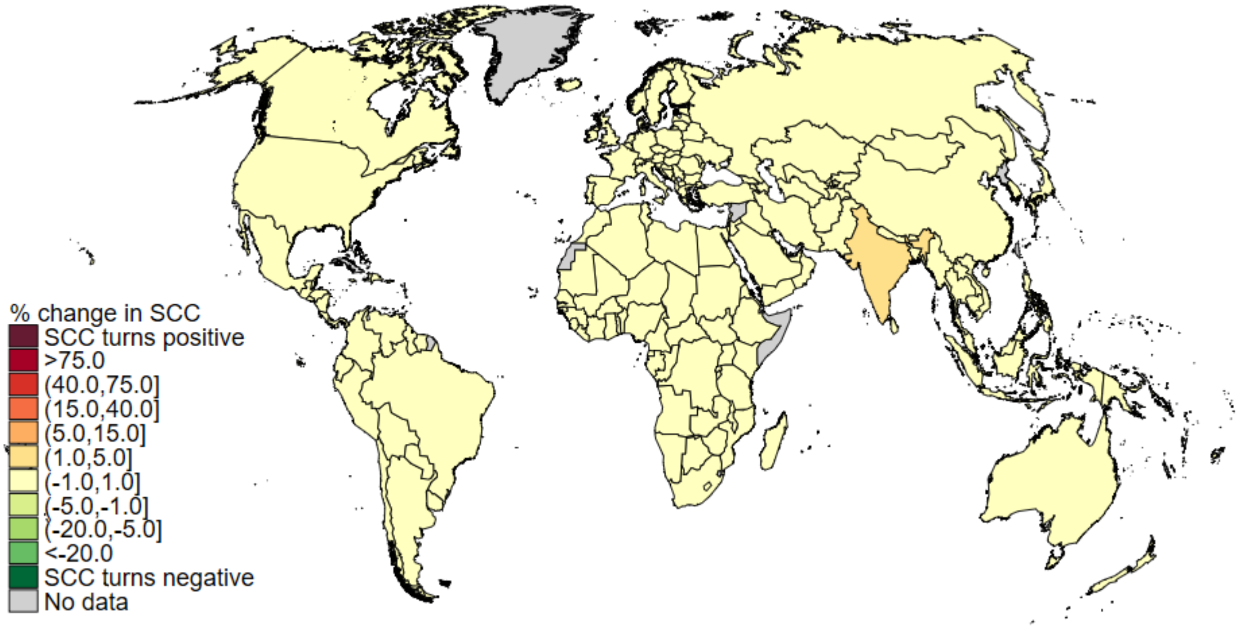

3.4 Effect of tipping points on inequality

As discussed in the main text, tipping points affect inequality only marginally. Figure 31 plots the Lorenz curve with and without all tipping points, normalised to the global SCC for comparability. As can be seen, there is little change to how unequally climate impacts are distributed across the world population.

Figure 31: Lorenz curve illustrating inequality in country-level SCC with and without tipping points, normalised to the respective cumulative global SCC for comparability. Specification comprises: RCP4.5-SSP2 emissions and GDP/population growth; Hope and Schaefer PCF; Whiteman et al. beta OMH; IPSL AMOC hosing. Monte Carlo sample size is 10,000. Welfare changes are normalised to global mean consumption per capita to enable comparison with other SCC estimates in the literature.

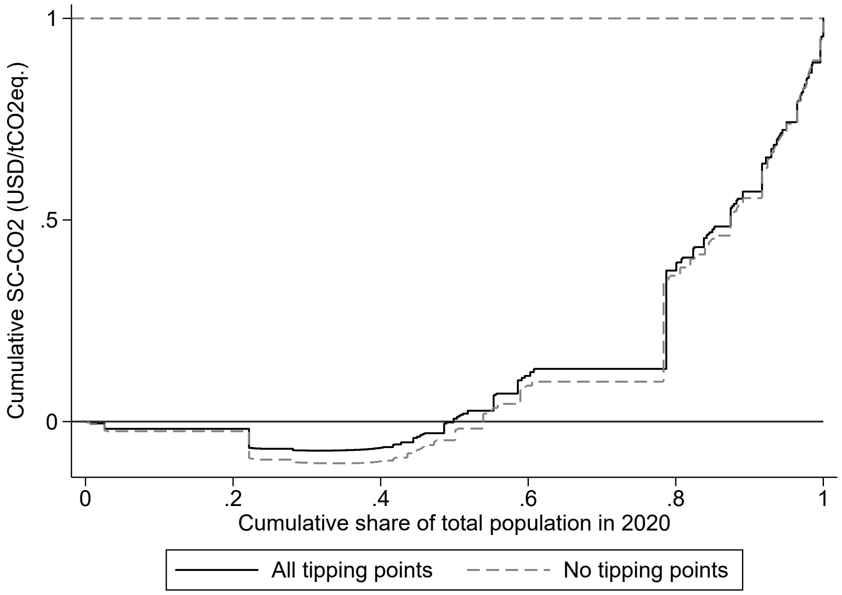

3.5 Effect of tipping points on warming and sea level rise

Figures 32-39 plot the effect of each tipping point individually on warming and SLR. Each scatter plot shows the relationship between warming/SLR in the absence of tipping points and when each tipping point is activated. We fit the data with polynomials, including second- and third-order terms when significant.

Figure 32: Scatter plots of warming (top panel) and SLR (bottom panel) with and without the permafrost carbon feedback (calibrated on 27). Data are sampled on a decadal interval (2020, 2030,...,2200), using 1000 Monte Carlo simulations under each of the RCP4.5 and RCP8.5 emissions scenarios. Both relationships are linear.

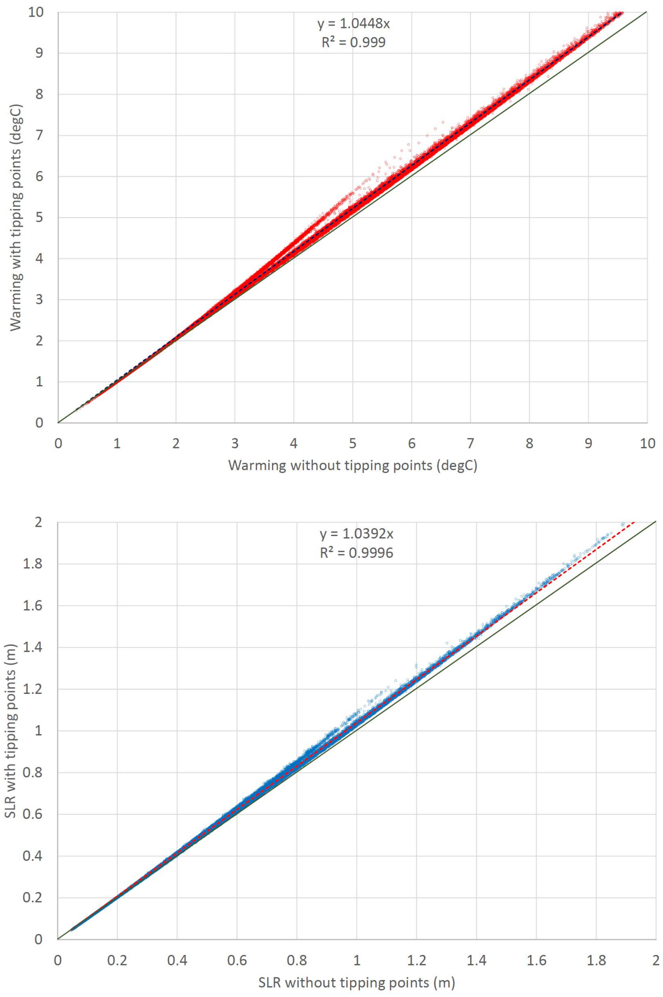

Figure 33: Scatter plots of warming (top panel) and SLR (bottom panel) with and without the dissociation of ocean methane hydrates (calibrated on 53). Data are sampled on a decadal interval (2020, 2030,...,2200), using 1000 Monte Carlo simulations under each of the RCP4.5 and RCP8.5 emissions scenarios. The temperature relationship is well approximated by a third-order polynomial. The SLR relationship is linear.

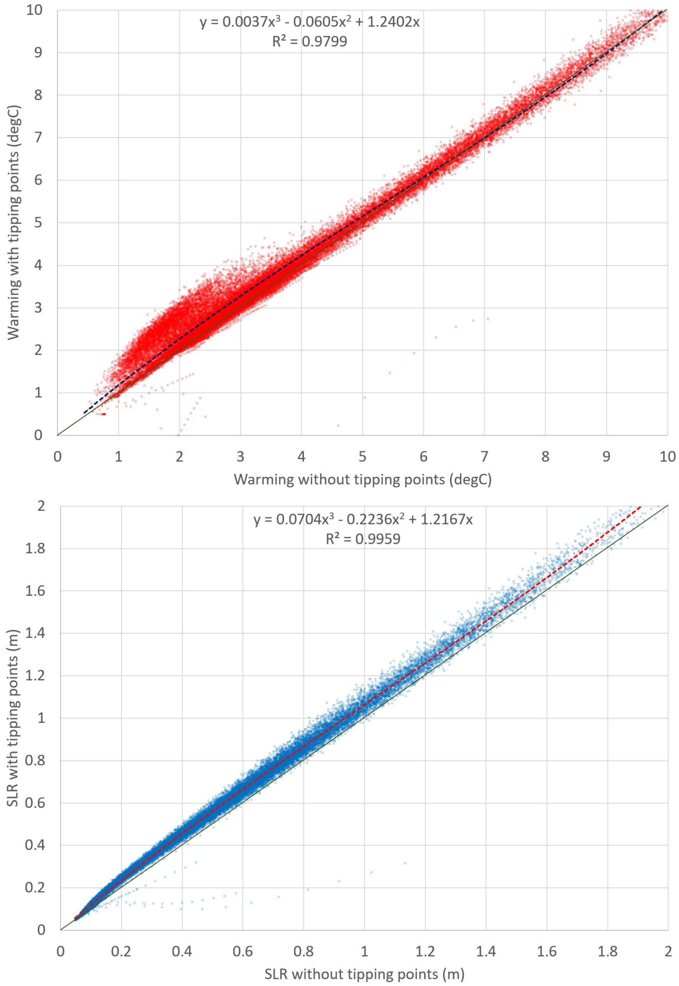

Figure 34: Scatter plots of warming (top panel) and SLR (bottom panel) with and without Amazon rainforest dieback. Data are sampled on a decadal interval (2020, 2030,...,2200), using 1000 Monte Carlo simulations under each of the RCP4.5 and RCP8.5 emissions scenarios. Both relationships are linear.

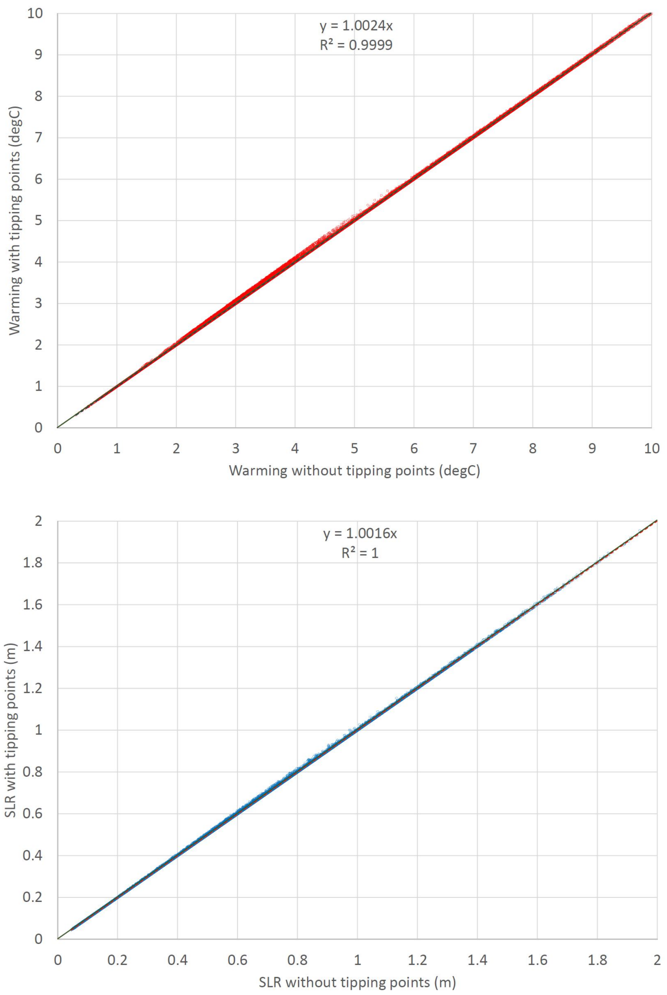

Figure 35: Scatter plots of warming (top panel) and SLR (bottom panel) with and without disintegration of the Greenland Ice Sheet. Data are sampled on a decadal interval (2020, 2030,...,2200), using 1000 Monte Carlo simulations under each of the RCP4.5 and RCP8.5 emissions scenarios. GIS disintegration has no effect on temperature in our model. The SLR relationship is non-linear and well approximated by a second-order polynomial.

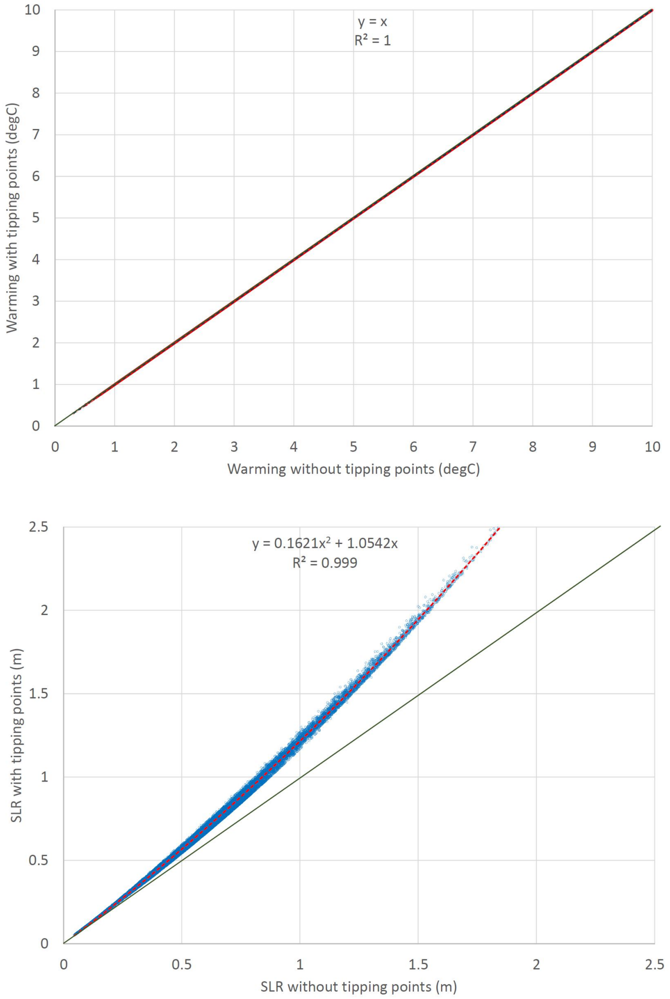

Figure 36: Scatter plots of warming (top panel) and SLR (bottom panel) with and without disintegration of the West Antarctic Ice Sheet. Data are sampled on a decadal interval (2020, 2030,...,2200), using 1000 Monte Carlo simulations under each of the RCP4.5 and RCP8.5 emissions scenarios. WAIS disintegration has no effect on temperature in our model. The SLR relationship is linear with a comparatively large scatter.

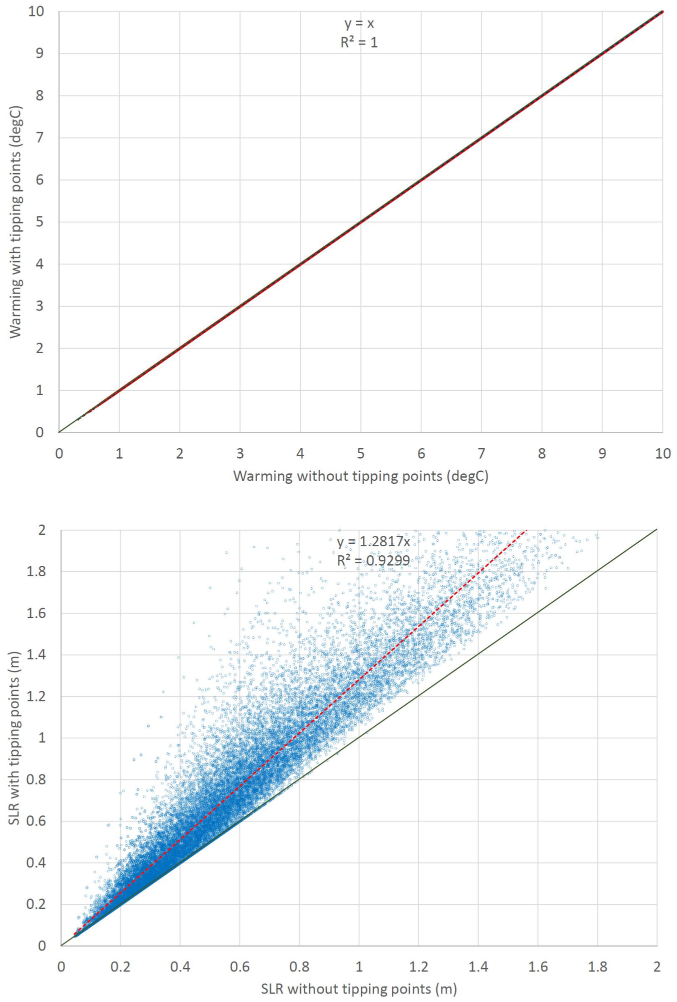

Figure 37: Scatter plots of warming (top panel) and SLR (bottom panel) with and without slowdown of the Atlantic Meridional Overturning Circulation. Data are sampled on a decadal interval (2020, 2030,...,2200), using 1000 Monte Carlo simulations under each of the RCP4.5 and RCP8.5 emissions scenarios. AMOC slowdown has no effect on temperature or SLR in our model.

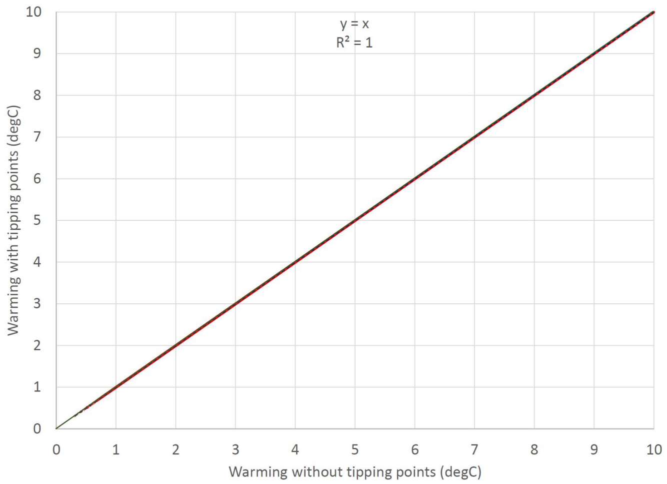

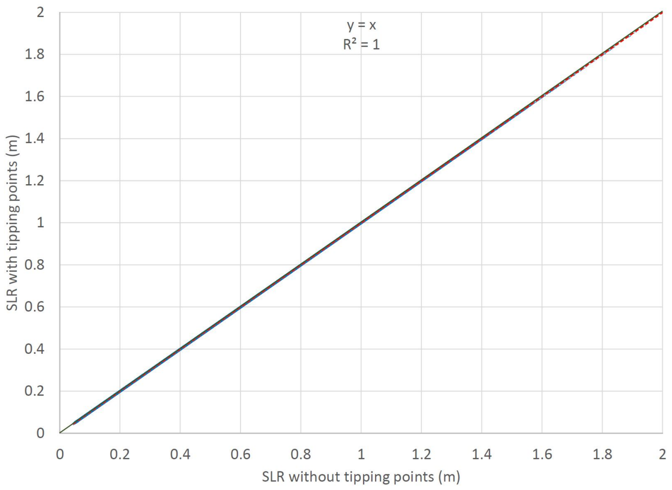

Figure 38: Scatter plots of warming (top panel) and SLR (bottom panel) with and without weakening of the Indian Summer Monsoon. Data are sampled on a decadal interval (2020, 2030,...,2200), using 1000 Monte Carlo simulations under each of the RCP4.5 and RCP8.5 emissions scenarios. ISM weakening has no effect on temperature or SLR in our model.

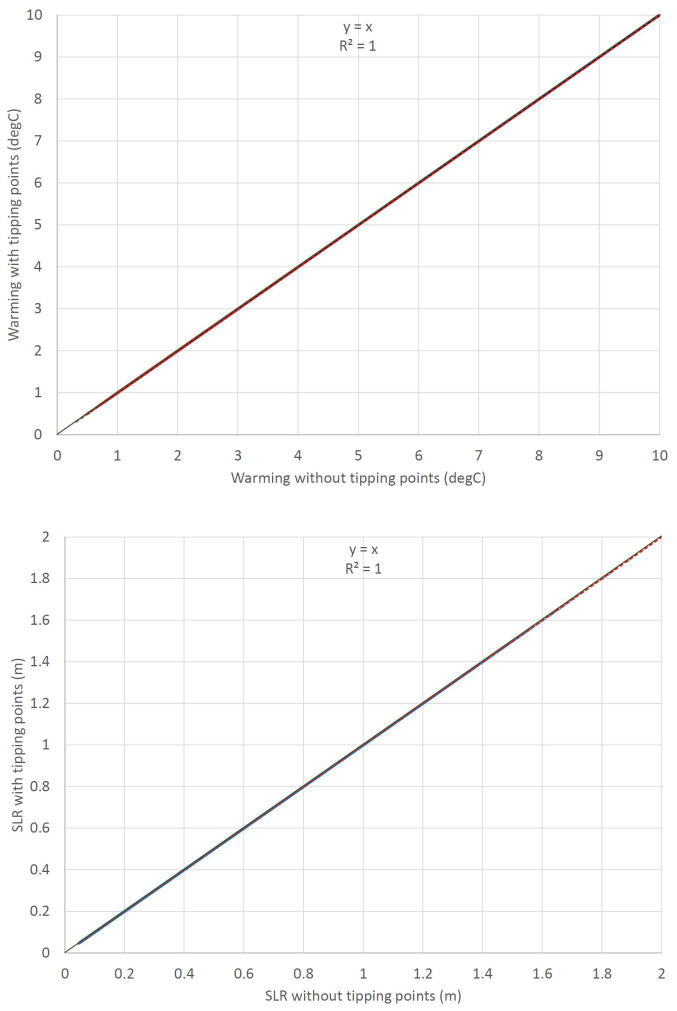

Figure 39: Scatter plots of warming (top panel) and SLR (bottom panel) with and without the surface albedo feedback. Data are sampled on a decadal interval (2020, 2030,...,2200), using 1000 Monte Carlo simulations under each of the RCP4.5 and RCP8.5 emissions scenarios. The temperature relationship is non-linear and well approximated by a second-order polynomial. The SLR relationship is linear.

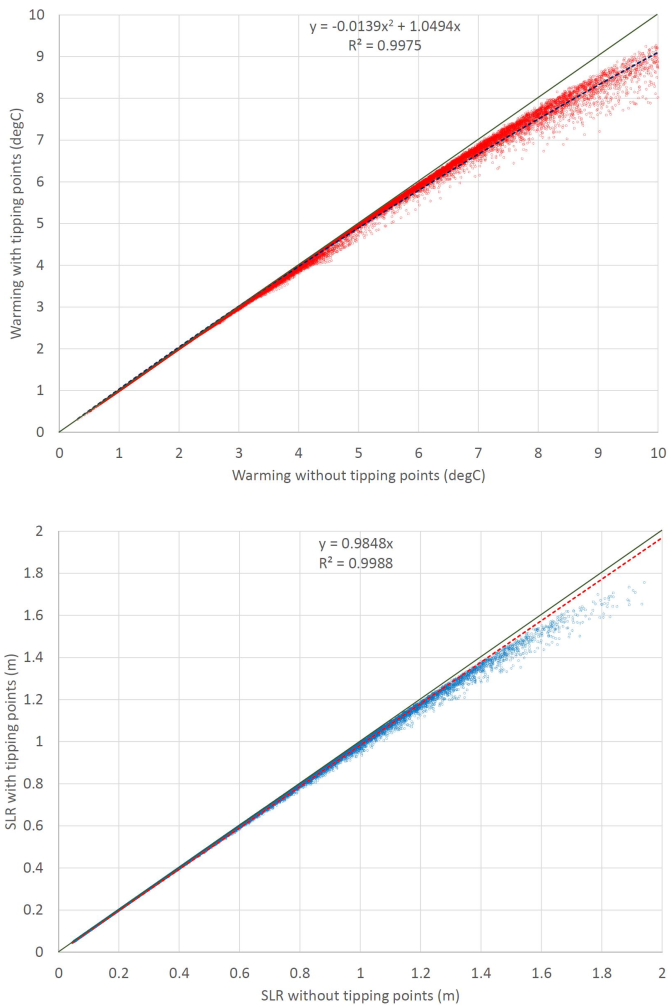

3.6 Analysis of Monte Carlo sample size and trimming the SCC distribution

Most of our results are obtained from a sample of size 10,000. Table 15 shows variation in some of our main output variables – the SCC, consumption/capita (mean and standard deviation), temperature and SLR – over 10 x 10,000 samples. By combining samples, we also show results for samples of size 20,000, 50,000 and 100,000.

The results exhibit a high degree of consistency across samples. Mean consumption per capita in 2100 has a standard deviation of 42 cents without tipping points and 83 cents with tipping points (i.e. this is the standard deviation of the sample means, rather than the within-sample standard deviation, which is also reported). The mean effect of tipping points on 2100 temperature varies by much less than 0.001°C across samples and the mean effect of tipping points on 2100 SLR varies by much less than 1cm. The standard deviation of the expected SCC (0.1% trimmed) without tipping points is two cents across samples; with tipping points it is 27 cents. The difference in the expected SCC due to tipping points has a standard deviation of 0.8 percentage points across samples.

Lastly, Table 16 reports the difference in the expected SCC for different degrees of trimming/truncation of the SCC distribution. We trim the distribution of SCCs as a pragmatic way to discard implausible values, which can be the result of implausible combinations of input variables. Ideally one would truncate the input distributions, however there are so many that it is too difficult to establish which combinations are implausible *a priori*. As Table 16 shows, the change in the expected SCC from the inclusion of tipping points rises the less the distribution is trimmed, which reflects the skew in the SCC distribution, albeit the effect is not large. In our judgement, all but a handful of runs are legitimate, therefore we seek to trim the distribution as little as possible. From trial and error, we established that trimming 0.1% of the distribution (i.e. 10 runs out of 10,000) excluded large negative values of the SCC and some extremely large positive values.

Table 15: Key results for different Monte Carlo sample sizes. Specification comprises: RCP4.5-SSP2 emissions and GDP/population growth; Hope and Schaefer PCF; Whiteman et al. beta OMH; IPSL AMOC hosing.

Sample number	Sample size	2100 global mean consumption per capita without TPs Mean	without TPs Std dev.	with TPs Mean	with TPs Std. dev.	2100 GMST increase due to TPs	2100 SLR increase due to TPs	SCC w/o TPs	SCC with TPs	SCC % increase
1	10,000	71191.77	422.22	70794.97	560.63	0.2867	0.6175	52.02	63.40	24.5
2	10,000	71191.97	427.80	70795.74	565.26	0.2862	0.6173	52.01	62.90	23.5
3	10,000	71191.53	425.02	70797.09	561.24	0.2860	0.6137	52.00	62.96	23.6
4	10,000	71192.38	426.59	70796.88	562.44	0.2864	0.6159	51.95	62.71	22.8
5	10,000	71192.38	420.17	70795.46	559.33	0.2867	0.6171	52.01	62.92	23.7
6	10,000	71191.86	424.97	70795.67	558.91	0.2868	0.6162	52.00	62.75	23.0
7	10,000	71192.04	422.93	70794.64	560.52	0.2860	0.6186	52.01	62.83	23.5
8	10,000	71193.05	420.98	70796.86	559.19	0.2860	0.6159	51.97	63.37	24.6
9	10,000	71192.21	424.99	70796.86	562.70	0.2866	0.6155	51.99	62.47	21.6
10	10,000	71192.58	421.79	70795.82	559.41	0.2858	0.6180	51.97	62.75	23.3
1+2	20,000	71191.87	425.01	70795.36	562.95	0.2865	0.6174	52.01	63.12	23.9
1+2+3+4+5	50,000	71192.00	424.36	70796.03	561.78	0.2864	0.6163	52.01	63.12	23.4
All	100,000	71192.18	423.75	70796.00	560.96	0.2863	0.6166	51.99	62.84	23.2

Table 16: The percentage change in the expected SCC due to all tipping points combined, for different degrees of trimming the SCC distribution. Specification comprises: RCP4.5-SSP2 emissions and GDP/population growth; Hope and Schaefer PCF; Whiteman et al. beta OMH; IPSL AMOC hosing.

	% increase due to TPs
1% trimmed	20.3
0.5% trimmed	21.9
0.1% trimmed	24.5
untrimmed	25.6

References

[1] T. M. Lenton, *et al.*, *Proceedings of the National Academy of Sciences* **105**, 1786 (2008).

[2] W. D. Nordhaus, *The climate casino* (Yale University Press, New Haven, 2013). OCLC: 935005305.

[3] W. Nordhaus, *Proceedings of the National Academy of Sciences* **116**, 12261 (2019).

[4] A. Robinson, R. Calov, A. Ganopolski, *Nature Climate Change* **2**, 429 (2012).

[5] D. B. Diaz, *Climatic Change* **137**, 143 (2016).

[6] D. Anthoff, F. Estrada, R. S. Tol, *American Economic Review: Papers and Proceedings* **106**, 602 (2016).

[7] C. Azar, K. Lindgren, *Climatic Change* **56**, 245 (2003). Publisher: Springer Science & Business Media.

[8] O. Bahn, N. R. Edwards, R. Knutti, T. F. Stocker, *Energy Policy* **39**, 334 (2011).

[9] A. Baranzini, M. Chesney, J. Morisset, *Energy Policy* **31**, 691 (2003).

[10] M. Belaia, Integrated assessment of climate tipping points, Ph.D. thesis, Universität Hamburg Hamburg (2017).

[11] M. Belaia, M. Funke, N. Glanemann, *Environmental and Resource Economics* **67**, 93 (2017).

[12] L. Berger, J. Emmerling, M. Tavoni, *Management Science* **63**, 749 (2017).

[13] J. E. Bickel, *Environment Systems & Decisions* **33**, 152 (2013).

[14] Y. Cai, K. L. Judd, T. M. Lenton, T. S. Lontzek, D. Narita, *Proceedings of the National Academy of Sciences* **112**, 4606 (2015).

[15] Y. Cai, T. M. Lenton, T. S. Lontzek, *Nature Climate Change* **6**, 520 (2016).

[16] Y. Cai, T. S. Lontzek, *Journal of Political Economy* **127**, 2684 (2019). Publisher: The University of Chicago Press.

[17] Y. Cai, W. Brock, A. Xepapadeas, Climate Change Economics and Heat Transport across the Globe: Spatial-DSICE, *Tech. rep.* (2016).

[18] M. Ceronsky, D. Anthoff, C. Hepburn, R. S. J. Tol, Checking the price tag on catastrophe: The social cost of carbon under non-linear climate response, *Working Paper 392*, ESRI working paper (2011).

[19] H.-P. Chao, *Risk Analysis* **15**, 69 (1995). _eprint: https://onlinelibrary.wiley.com/doi/pdf/10.1111/j.1539-6924.1995.tb00094.x.

[20] D. Diaz, K. Keller, *American Economic Review Papers & Proceedings* **106**, 607 (2016).

[21] P. Dumas, M. Hu-Duong, *The Coupling of Climate and Economic Dynamics: Essays on Integrated Assessment*, A. Haurie, L. Viguier, eds. (2005), pp. 97–112.

[22] G. Engström, J. Gars, *Environmental and Resource Economics* **65**, 541 (2016).

[23] J. Gjerde, S. Grepperud, S. Kverndokk, *Resource and Energy Economics* **21**, 289 (1999).

[24] M. González-Eguino, M. B. Neumann, I. Arto, I. Capellán-Perez, S. H. Faria, *Earth's Future* **5**, 59 (2017).

[25] M.-L. Guillerminet, R. S. J. Tol, *Climatic Change* **91**, 193 (2008).

[26] G. Heutel, J. Moreno-Cruz, S. Shayegh, *Journal of Economic Behavior & Organization* **132**, 19 (2016).

[27] C. Hope, K. Schaefer, *Nature Climate Change* **6**, 56 (2016).

[28] K. Keller, K. Tan, F. M. Morel, D. F. Bradford, *Climatic Change* **47**, 14 (2000).

[29] K. Keller, B. M. Bolker, D. F. Bradford, *Journal of Environmental Economics and Management* **48**, 723 (2004).

[30] L. Kessler, *Climate Change Economics* **8**, 1750008 (2017).

[31] F. Lamperti, G. Dosi, M. Napoletano, A. Roventini, A. Sapio, *SSRN Electronic Journal* (2017).

[32] R. J. Lempert, A. H. Sanstad, M. E. Schlesinger, *Energy Economics* **28**, 677 (2006).

[33] D. Lemoine, C. Traeger, *American Economic Journal: Economic Policy* **6**, 137 (2014).

[34] D. Lemoine, C. P. Traeger, *Nature Climate Change* **6**, 514 (2016).

[35] D. Lemoine, C. P. Traeger, *Journal of Economic Behavior & Organization* **132**, 5 (2016).

[36] P. M. Link, R. S. Tol, *Portuguese Economic Journal* **3**, 99 (2004).

[37] P. M. Link, R. S. J. Tol, *Climatic Change* **104**, 287 (2011).

[38] T. S. Lontzek, D. Narita, O. Wilms, *Environmental and Resource Economics* **65**, 573 (2016).

[39] T. S. Lontzek, Y. Cai, K. L. Judd, T. M. Lenton, *Nature Climate Change* **5**, 441 (2015).

[40] D. McInerney, R. Lempert, K. Keller, *Climatic Change* **112**, 547 (2012).

[41] E. Naevdal, *Journal of Economic Dynamics and Control* **30**, 1131 (2006).

[42] E. Naevdal, M. Oppenheimer, *Resource and Energy Economics* **29**, 262 (2007).

[43] R. J. Nicholls, R. S. J. Tol, A. T. Vafeidis, *Climatic Change* **91**, 171 (2008).

[44] W. D. Nordhaus, *Managing the Global Commons: the Economics of Climate Change* (MIT Press, Cambridge, MA, 1994).

[45] I. Nordin, Multiple tipping points in the climate system, Master's thesis, Umea Universitet (2014).

[46] S. C. Peck, T. J. Teisberg, *Energy Policy* **23**, 297 (1995).

[47] J. Pycroft, L. Vergano, C. Hope, *Global Environmental Change* **24**, 99 (2014).

[48] M. E. Schlesinger, *et al.*, *Avoiding Dangerous Climate Change* (Cambridge University Press, Cambridge, UK, 2006), pp. 37–47.

[49] S. Shayegh, V. M. Thomas, *Climatic Change* **128**, 1 (2015).

[50] C. Sims, D. Finnoff, *Journal of the Association of Environmental and Resource Economists* **3**, 985 (2016).

[51] F. van der Ploeg, *European Economic Review* **67**, 28 (2014).

[52] F. van der Ploeg, A. de Zeeuw, *Journal of the European Economic Association* (2017).

[53] G. Whiteman, C. Hope, P. Wadhams, *Nature* **499**, 401 (2013).

[54] H. Wirths, J. Rathmann, P. Michaelis, *Environmental Economics and Policy Studies* **20**, 109 (2018).

[55] G. Yohe, *Global Environmental Change* **6**, 87 (1996).

[56] G. W. Yohe, M. E. Schlesinger, N. G. Andronova, *Integrated Assessment* **6** (2006). Number: 1.

[57] D. Yumashev, *et al.*, *Nature Communications* **10**, 1900 (2019).

[58] N. Shakhova, V. Alekseev, I. Semiletov, *Doklady Earth Sciences* **430**, 190 (2010).

[59] M. Ceronsky, D. Anthoff, C. Hepburn, R. S. Tol, Checking the price tag on catastrophe: the social cost of carbon under non-linear climate response, *Tech. rep.*, ESRI working paper (2011).

[60] Y. Cai, T. M. Lenton, T. S. Lontzek, *Nature Climate Change* **6**, 520 (2016).

[61] D. Diaz, K. Keller, *American Economic Review: Papers and Proceedings* **106**, 607 (2016).

[62] D. Archer, B. Buffett, V. Brovkin, *Proceedings of the National Academy of Sciences* **106**, 20596 (2009).

[63] D. Archer, *Biogeosciences* **12**, 2953 (2015).

[64] E. Kriegler, J. W. Hall, H. Held, R. Dawson, H. J. Schellnhuber, *Proceedings of the National Academy of Sciences* **106**, 5041 (2009).

[65] R. B. Alley, *et al.*, *Quaternary Science Reviews* **29**, 1728 (2010).

[66] O. Bahn, N. R. Edwards, R. Knutti, T. F. Stocker, *Energy Policy* **39**, 334 (2011).

[67] P. M. Link, R. S. Tol, *Climatic Change* **104**, 287 (2011).

[68] T. M. Lenton, J.-C. Ciscar, *Climatic Change* **117**, 585 (2013).

[69] T. Stocker, *et al.*, *Climate Change 2013: The Physical Science Basis. Contribution of Working Group I to the Fifth Assessment Report of the Intergovernmental Panel on Climate Change*, T. Stocker, *et al.*, eds. (Cambridge University Press, Cambridge, United Kingdom and New York, NY, USA, 2013), pp. 33–115.

[70] O. Hoegh-Guldberg, *et al.*, *Global Warming of 1.5° C. An IPCC Special Report on the impacts of global warming of 1.5° C above pre-industrial levels and related global greenhouse gas emission pathways, in the context of strengthening the global response to the threat of climate change, sustainable development, and efforts to eradicate poverty*, V. Masson-Delmotte, *et al.*, eds. (in press, 2018).

[71] S. N. Gosling, *Environmental Science & Policy* **27**, S15 (2013).

[72] M. Ikefuji, J. R. Magnus, H. Sakamoto, *Climatic Change* **126**, 229 (2014).

[73] J. Schewe, A. Levermann, *Environmental Research Letters* **7**, 044023 (2012).

[74] R. H. Moss, *et al.*, *Nature* **463**, 747 (2010).

[75] C. Li, *et al.*, *Scientific Reports* **7**, 14304 (2017).

[76] M. Meinshausen, *et al.*, *Climatic Change* **109**, 213 (2011).

[77] R. J. Millar, Z. R. Nicholls, P. Friedlingstein, M. R. Allen, *Atmospheric Chemistry and Physics* **17**, 7213 (2017).

[78] F. Joos, *et al.*, *Atmospheric Chemistry and Physics* **13**, 2793 (2013).

[79] G. Myhre, *et al.*, *Climate Change 2013: The Physical Science Basis. Contribution of Working Group I to the Fifth Assessment Report of the Intergovernmental Panel on Climate Change*, T. Stocker, *et al.*, eds. (IPCC, 2013), pp. 8SM–1–8SM–44.

[80] K. Shine, R. Derwent, D. Wuebbles, J.-J. Morcrette, *et al.*, *Climate Change: the IPCC Scientific Assessment. Report Prepared for IPCC by Working Group 1* ., J. Houghton, G. Jenkins, J. Ephraums, eds. (Cambridge University Press, Cambridge, 1990), pp. 41–68.

[81] O. Geoffroy, *et al.*, *Journal of Climate* **26**, 1841 (2013).

[82] J. A. Church, N. J. White, *Surveys in Geophysics* **32**, 585 (2011).

[83] M. Burke, S. M. Hsiang, E. Miguel, *Nature* **527**, 235 (2015).

[84] B. C. O'Neill, *et al.*, *Climatic Change* **122**, 387 (2014).

[85] S. Dietz, N. Stern, *Economic Journal* **125**, 574 (2015).

[86] M. Golosov, J. Hassler, P. Krusell, A. Tsyvinski, *Econometrica* **82**, 41 (2014).

[87] World Bank, World development indicators (2020).

[88] W. D. Nordhaus, J. Boyer, *Warming the World: Economic Models of Global Warming* (MIT Press (MA), 2000).

[89] A. S. Manne, R. G. Richels, *Energy and environment* (Springer, 2005), pp. 175–189.

[90] T. Carleton, *et al.* (2018).

[91] E. A. Bright, P. R. Coleman, A. N. Rose, M. L. Urban, LandScan 2011 (2012). Digital dataset: `web.ornl.gov/sci/landscan/index.shtml`.

[92] B. Thrasher, E. Maurer, C. McKellar, P. Duffy, *Hydrol. Earth Syst. Sci* **16**, 3309 (2012).

[93] J. A. Church, *et al.*, *Climate Change 2013: the Physical Science Basis. Contribution of Working Group I to the Fifth Assessment Report of the Intergovernmental Panel on Climate Change*, T. F. Stocker, *et al.*, eds. (Cambridge University Press, Cambridge, UK and New York, NY, USA, 2013), pp. 1137–1216.

[94] K. Riahi, *et al.*, *Global Environmental Change* **42**, 153 (2017).

[95] K. Ricke, L. Drouet, K. Caldeira, M. Tavoni, *Nature Climate Change* **8**, 895 (2018).

[96] N. Stern, *The Economics of Climate Change: the Stern Review* (Cambridge University Press, 2007).

[97] M. L. Weitzman, *Review of Economics and Statistics* **91**, 1 (2009).

[98] S. Dietz, *Climatic Change* **103**, 519 (2011).

[99] A. Millner, *Journal of Environmental Economics and Management* **65**, 310 (2013).
